# Copper-Catalyzed Azide–Alkyne Cycloaddition (CuAAC) by Functionalized NHC-Based Polynuclear Catalysts: Scope and Mechanistic Insights

**DOI:** 10.1021/acs.organomet.2c00246

**Published:** 2022-07-15

**Authors:** Miguel González-Lainez, Miguel Gallegos, Julen Munarriz, Ramón Azpiroz, Vincenzo Passarelli, M. Victoria Jiménez, Jesús J. Pérez-Torrente

**Affiliations:** † Departamento de Química Inorgánica, Instituto de Síntesis Química y Catálisis Homogénea-ISQCH, Universidad de Zaragoza-C.S.I.C., 50009 Zaragoza, Spain; ‡ Departamento de Química Física y Analítica, Universidad de Oviedo, 33006 Oviedo, Spain

## Abstract

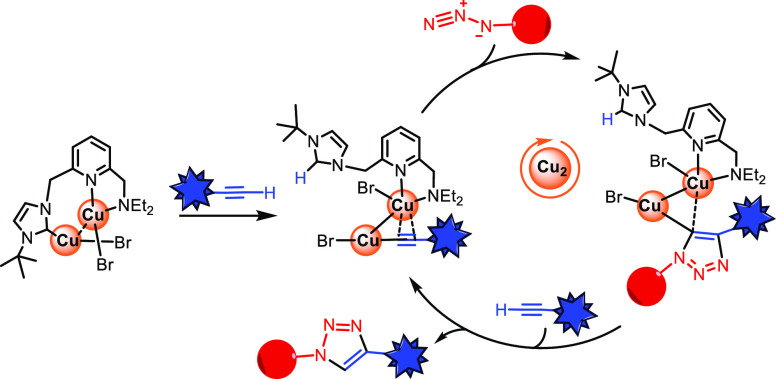

Copper(I) [Cu_2_(μ-Br)_2_(^t^BuImCH_2_pyCH_2_L)]_*n*_ (L = OMe, NEt_2_, NH^t^Bu) compounds supported by flexible functionalized NHC-based polydentate ligands have been prepared in a one-pot procedure by reacting the corresponding imidazolium salt with an excess of copper powder and Ag_2_O. An X-ray diffraction analysis has revealed that [Cu_2_(μ-Br)_2_(^t^BuImCH_2_pyCH_2_NEt_2_)]_*n*_ is a linear coordination polymer formed by bimetallic [Cu(μ-Br)]_2_ units linked by the lutidine-based NHC-py-NEt_2_ ligand, which acts as a heteroditopic ligand with a 1κ*C*-2κ^2^*N,N′* coordination mode. We propose that the polymeric compounds break down in the solution into more compact tetranuclear [Cu_2_(μ-Br)_2_(^t^BuImCH_2_pyCH_2_L)]_2_ compounds with a coordination mode identical to the functionalized NHC ligands. These compounds have been found to exhibit high catalytic activity in the Cu-catalyzed azide–alkyne cycloaddition (CuAAC) reaction. In particular, [Cu_2_(μ-Br)_2_(^t^BuImCH_2_pyCH_2_NEt_2_)]_2_ efficiently catalyzes the click reaction of a range of azides and alkynes, under an inert atmosphere at room temperature in neat conditions at a very low catalyst loading, to quantitatively afford the corresponding 1,4-disubstituted 1,2,3-triazole derivatives in a few minutes. The cycloaddition reaction of benzyl azide to phenylacetylene can be performed at 25–50 ppm catalyst loading by increasing the reaction time and/or temperature. Reactivity studies have shown that the activation of the polynuclear catalyst precursor involves the alkyne deprotonation by the NHC moiety of the polydentate ligand to afford a copper(I)-alkynyl species bearing a functionalized imidazolium ligand. DFT calculations support the participation of the dinuclear species [(CuBr)_2_(μ-^t^BuImCH_2_pyCH_2_NEt_2_)], resulting from the fragmentation of the tetranuclear compound, as the catalytically active species. The proposed reaction pathway proceeds through zwitterionic dinuclear intermediates and entails the active participation of both copper atoms, as well as the NHC moiety as an internal base, which activates the reacting alkyne via deprotonation.

## Introduction

The copper-catalyzed azide–alkyne cycloaddition (CuAAC) reaction has greatly expanded the toolbox of synthetic organic chemistry.^1^ This process selectively transforms organic azides and terminal alkynes into the corresponding 1,4-disubstituted 1,2,3-triazoles under mild reaction conditions, thus fulfilling the criteria of ″click chemistry″ defined by Sharpless et al.^2^ In contrast, the noncatalyzed version, i.e., the Huisgen thermal reaction, proceeds at much higher temperatures and affords mixtures of 1,4- and 1,5-disubstituted triazole regioisomers.^3^ This synthetic protocol, which provides access to complex organic architectures from readily available building blocks, has found numerous applications in such important disciplines as modern organic chemistry, nanotechnology, chemical biology, medicinal chemistry, drug discovery, and materials science.^4^

It is well established that the CuAAC reaction proceeds through copper(I) active species that have a limited stability. Thus, the addition of ligands with the ability to stabilize the copper(I) intermediates or, alternatively, the use of well-defined copper(I) catalysts generally results in the improvement of the catalytic efficiency. In fact, a number of N-, P-, and S-based mono- and polydentate ligands with distinct stereoelectronic properties have been thoroughly investigated.^5^ Research in CuAAC has experienced a spectacular growth in recent years as a consequence of (i) the preparation of well-defined copper(I) catalysts, (ii) the availability and broad applicability of straightforward protocols to access 1,2,3-triazole derivatives, and (iii) experimental and theoretical studies to unravel the reaction mechanism.^6^

N-Heterocyclic carbenes (NHCs) have become essential ligands for transition-metal catalysis owing to their strong coordination ability as well as electronic and steric modularity.^7^ In fact, Cu(NHC)-mediated catalysis has experienced an intense growth with an increase in the number of applications.^8^ In this regard, N-heterocyclic carbene-based copper complexes of type [(NHC)CuX]^9^ and [(NHC)_2_Cu]X^10^ have been proven to be very efficient for the click cycloaddition reaction between azides and alkynes under very mild conditions. Remarkably, NHC ligands significantly increase the stability of copper(I) intermediates, allowing for the reduction of the catalyst loading while maintaining a notable catalytic activity. In addition, copper(I) complexes bearing mesoionic NHC carbenes^11^ and related dinuclear counterparts^12^ have also been found to be excellent catalysts for the CuAAC reaction. In sharp contrast, copper catalyst precursors based on functionalized NHC ligands are, to our knowledge, much more scarce and limited to NHC ligands having thioether, sulfoxide, or sulfone wingtips^13^ or polar groups to impart water solubility.^14^

**Figure 1:**
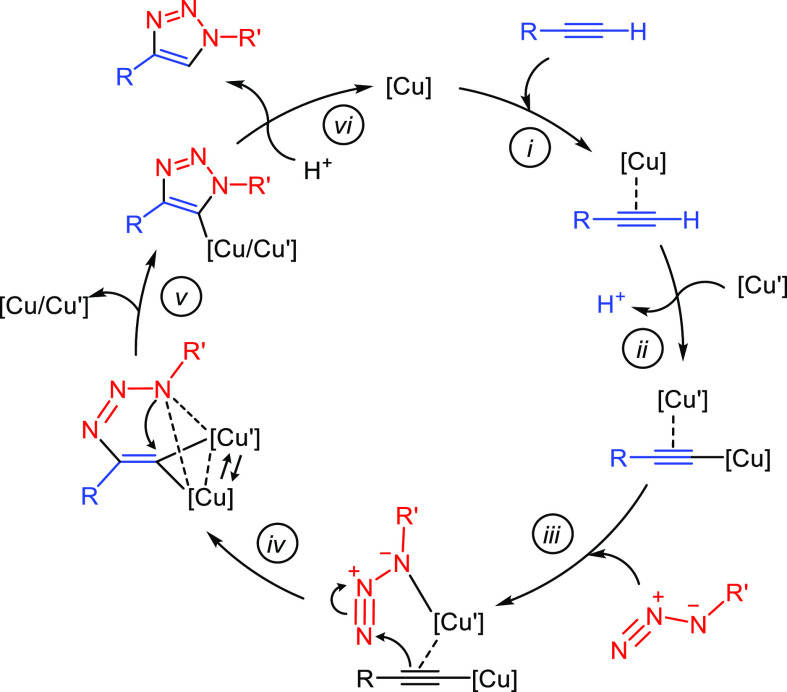
Mechanism of the CuAAC reaction proposed by Fokin *et al*.^19^

The first mechanistic studies of such processes were performed by Sharpless and co-workers in 2002^15^ and were complemented later by several theoretical and experimental studies.^16^ Based on DFT studies, Fokin *et al.* proposed that the presence of a second copper atom favors the reaction,^17^ which was further supported from an experimental point of view.^18,19^ This information led Fokin to propose the reaction mechanism depicted in Figure 1, which highlights the active participation of both copper atoms. The isolation of several dinuclear σ,π-bis(copper)alkynyl species that had been proposed as reaction intermediates further supported the mechanism.^19,20^

These findings have increased the interest in mechanistic studies of CuAAC processes.^21^ As a result, several groups have reported very significant experimental^22^ and theoretical studies,^23^ which mainly focus on identifying, isolating, and characterizing resting states^24^ with special interest in the nuclearity and the explicit role of the copper atoms involved in the process.^22a,25^ This has led to a relatively well-established Fokin-like catalytic cycle, although some authors have proposed some modifications, for example, the coordination of the azide to the Cu atom σ-bound to the alkynyl group instead of to the ligand-bound Cu center (Cu′ in Figure 1).^23f^

In this context, herein we report the synthesis of copper(I) coordination polymers supported by tridentate NHC-py-L (L = OMe, NEt_2_, NH^t^Bu) ligands and their application in the base-free CuAAC reaction. In addition, we have performed DFT and reactivity studies to determine the possible reaction mechanism as a further step to the complete understanding of the CuAAC processes.

## Results and Discussion

### Synthesis of Imidazolium Salt Precursors for Tridentante NHC-py-L (L = OMe, NEt_2_, NH^t^Bu) Ligands

**Scheme 1:**
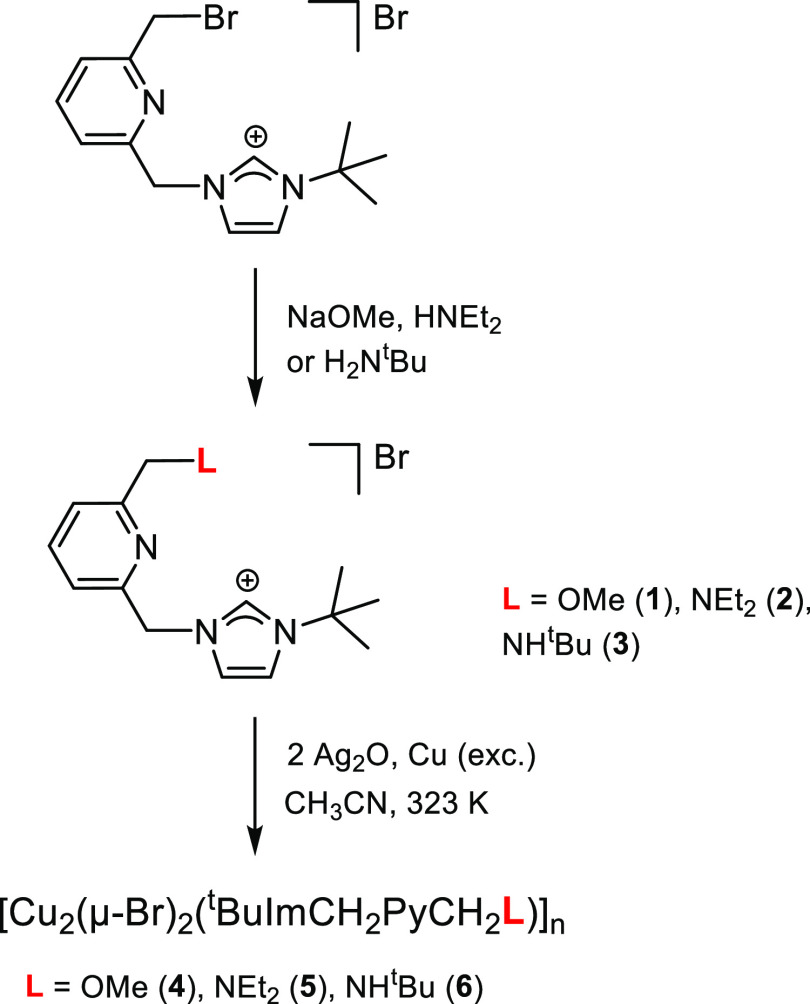
Synthetic Pathway for the Preparation of Copper(I) Complexes Supported by Lutidine-Based NHC/L Functionalized Ligands

The functionalized imidazolium salt precursors of flexible lutidine-derived polydentate ligands NHC-py-L (L = OMe, NEt_2_, NH^t^Bu) have been prepared from the imidazolium salt 1-((6-(bromomethyl)pyridin-2-yl)methyl)-3-(*tert*-butyl)-1*H*-imidazol-3-ium bromide.^26^ Thus, nucleophilic substitution at the bromomethyl group by sodium methoxide, dimethylamine, or *tert*-butylamine afforded the corresponding methoxy (**1**), diethylamino (**2**), and *tert*-butylamino (**3**) functionalized imidazolium salts, which were isolated as pale yellow solids in 80–95% yield (Scheme 1). Stoichiometric amounts of sodium methoxide and diethylamine were used in the synthesis of **1** and **2**, which were carried out at room temperature in methanol and acetonitrile, respectively. However, an excess of *tert*-butylamine was required in the synthesis of **3**, which was carried out in acetonitrile at 373 K.

The functionalized imidazolium salts **1**–**3** have been characterized by elemental analysis, mass spectrometry (HRMS-ESI), and ^1^H and ^13^C{^1^H} NMR spectroscopy. Particularly, the mass spectra show a peak that corresponds to the molecular ion. The ^1^H NMR spectra of imidazolium salts exhibit a series of characteristic resonances. Namely, a low-field signal at δ ≈ 11.0 ppm corresponding to the acidic proton of the imidazolium moiety, the =CH resonances of the pyridine and imidazole rings in the aromatic region, a signal at δ ≈ 1.8 ppm for the −^t^Bu substituent, and two singlets for the bridging >CH_2_ protons at δ ≈ 5.8 ppm (NHC-CH_2_-py) and 4.5–3.5 ppm (py-CH_2_-L). Furthermore, the spectra show the characteristic signals of the corresponding functional groups OMe, δ = 3.45 ppm (s); NEt_2_, δ = 2.54 (q), 1.03 ppm (t); and NH^t^Bu, δ = 1.69 (s, NH), 1.38 ppm (s, ^t^Bu).

### Synthesis of Copper(I) Complexes [Cu_2_(μ-Br)_2_(^t^BuImCH_2_pyCH_2_L)]_*n*_ (L = OMe, NEt_2_, NH^t^Bu)

The synthesis of copper(I) compounds based on the potentially tridentate ligands ^t^BuImCH_2_pyCH_2_L (L = OMe, NEt_2_, NH^t^Bu) has been approached following the synthetic strategy described by Chen *et al*.^27^ This methodology allows one to directly obtain organometallic compounds bearing NHC ligands from the metal powder of interest. This way, the one-pot reaction of the imidazolium salt with an excess of copper powder and Ag_2_O in acetonitrile for 30 h at 323 K afforded yellow solutions of complexes [Cu_2_(μ-Br)_2_(^t^BuImCH_2_pyCH_2_L)]_*n*_ (L = OMe, **4**; NEt_2_, **5**; NH^t^Bu, **6**) after removing any amount of unreacted copper powder and the newly formed metallic silver (Scheme 1). The compounds were isolated as pale brown (**4**) or pale green (**5** and **6**) air sensitive microcrystalline solids with yields close to 50% with respect to the imidazolium salt precursors. In this regard, it should be noted that compounds **4**–**6** incorporate two bromido ligands for each lutidine-based NHC/L scaffold, showing a mismatch in the stoichiometry of the reactions. We believe that this is partially responsible for the moderate yield achieved in the synthesis of the complexes. To improve this, the synthesis of **5** was carried out by adding 2.2 equiv of KBr to the reaction mixture, which allowed it to be increased to 68%.

**Figure 2:**
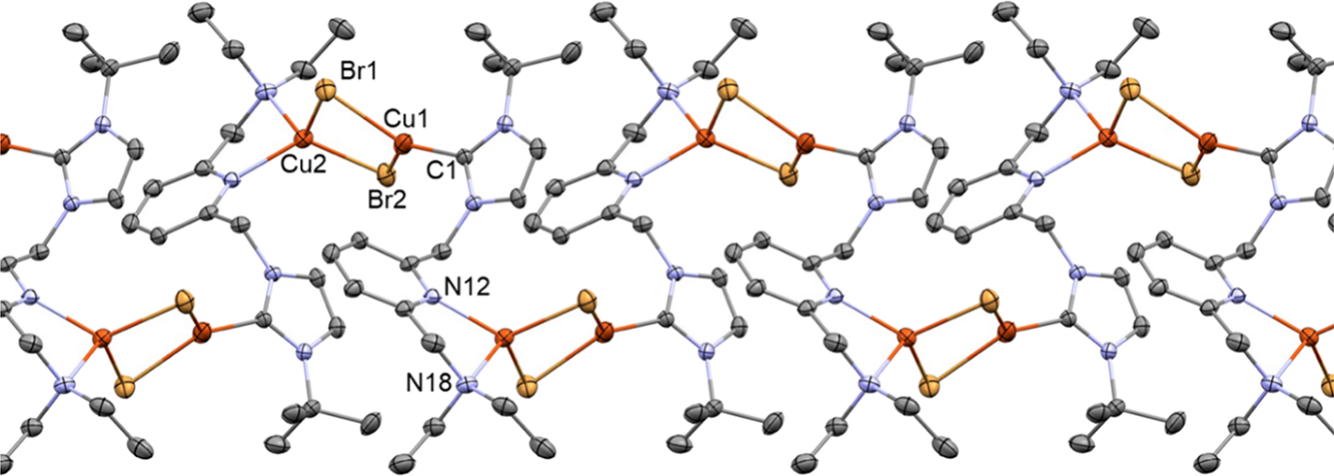
ORTEP views of the polymeric array of **5**. Thermal ellipsoids are at 50% probability. Hydrogen atoms are omitted for clarity.

**Figure 3:**
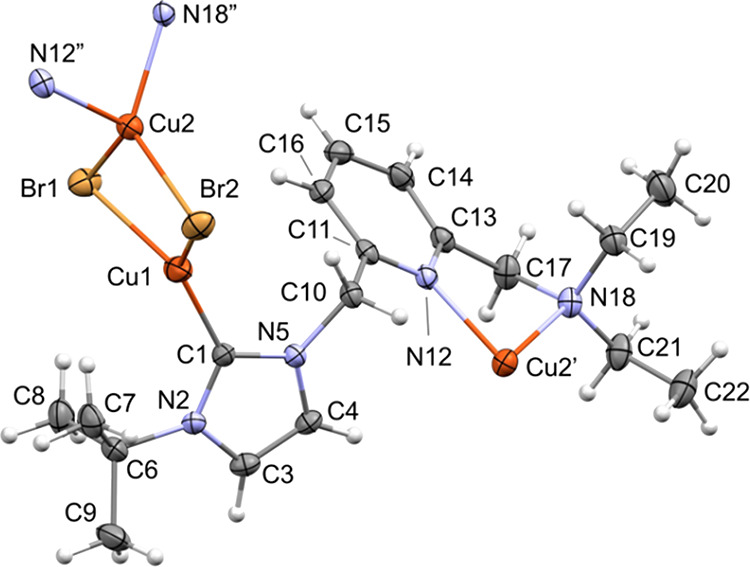
ORTEP view of the repeating unit of **5**. Thermal ellipsoids are at 50% probability. Selected bond lengths (Å) and angles (°) are as follows: Cu1-Br2 2.4226(6), Cu1-Br1 2.4603(6), Cu2-N12″ 2.064(3), Cu2-N18″ 2.174(3), Cu2-Br1 2.4133(5), Cu2-Br2 2.4430(6), Cu1···Cu2 2.8185(6), C1-Cu1-Br2 129.27(10), C1-Cu1-Br1 123.28(10), Br2-Cu1-Br1 107.45(2), N12″-Cu2-N18″ 82.44(11), N12″-Cu2-Br1 118.78(8), N18″-Cu2-Br1 115.65(8), N12″-Cu2-Br2 122.22(7), N18″-Cu2-Br2 106.25(8), and Br1-Cu2-Br2 108.31(2). Symmetry transformations used to generate equivalent atoms Cu2′, N12″, and N18″: ′ −*x* + 1/2, *y* + 1/2, −*z* + 1/2; ″ −*x* + 1/2, *y* – 1/2, −*z* + 1/2.

Single-crystal X-ray diffraction analysis has revealed that **5** is a linear coordination polymer of formula [Cu_2_(μ-Br)_2_(^t^BuImCH_2_pyCH_2_NMe_2_)]_*n*_ (Figures 2 and 3). Bimetallic units [Cu(μ-Br)]_2_ are linked by the lutidine-based ligand NHC-py-NEt_2_, which acts as a heteroditopic ligand with a 1κ*C*-2κ^2^*N,N′* coordination. The copper centers exhibit different coordination geometries, one being tricoordinate (Cu1) and the other tetracoordinate (Cu2). The intermetallic distance [Cu1···Cu2 2.8185(6) Å] is larger than twice the van der Waals radius of copper (*r*_Cu_ = 1.40 Å), thus ruling out any bond between both metal centers. The [Cu(μ-Br)]_2_ core deviates from planarity, featuring a puckering angle of 21.0° between the Cu2-Br1-Br2 and the Cu1-Br1-Br2 planes. On the one hand, the coordination sphere of Cu1 is completed by the C1 carbon atom of the NHC moiety, rendering a distorted trigonal planar geometry [C1-Cu1-Br2 129.27(10)°, C1-Cu1-Br1 123.28(10)°, Br2-Cu1-Br1 107.45(2)°] with the NHC core slightly deviating from the perpendicular arrangement with respect to the coordination plane [N2-C1-Cu1-Br1–73.9(3)°]. On the other hand, the copper center Cu2 is bonded to the pyridine nitrogen atom N12 and the amine nitrogen atom N18, exhibiting a distorted tetrahedral geometry (Figure 3). Finally, it is worth mentioning that the five-member ring Cu2′-N12-C13-C17-N18 adopts an envelope configuration [Cremer–Pople^28^ parameters: *q* 0.450(3) Å, Φ 141.3(4)°] and the pyridine fragment deviates from the ideal arrangement with respect to the Cu2′-N12 bond (pitch, θ 2.4°; yaw, ψ 9.5°).^29^ This is surely a consequence of the small bite angle of the pyridine-amine ligating site [N12″-Cu2-N18″ 82.44(11)°].

The compounds have been fully characterized by elemental analysis, mass spectrometry, and multinuclear NMR spectroscopy. The most noticeable feature of the ^1^H NMR spectra is the absence of the characteristic low field resonance of the imidazolium fragment, which confirms the formation of the Cu-NHC bond. Also, the carbenic carbon atom (NCN) is observed at around δ 178 ppm in the ^13^C{^1^H} NMR spectra. In view of the similarities of the NMR spectra, the three complexes seem to be isostructural in solution. In this regard, their ^1^H and ^13^C NMR spectra show the same signals for the pyridine and imidazol-2-ylidene moieties, the only differences arising from the characteristic resonances of the functional group L (L = OMe, NEt_2_, or NH^t^Bu), which appear at the expected chemical shifts in each case. Surprisingly, the >CH_2_ protons joining the pyridine ring with the imidazol-2-ylidene fragment appear as singlets at around δ 5.4–5.7 ppm despite the coordination of both groups. Besides, the methylene protons of py-CH_2_-L fragment give rise to a singlet at δ 4.51 and 3.75 ppm for **4** and **5**, respectively, and a multiplet at δ 4.07 ppm for **6**.

**Scheme 2:**
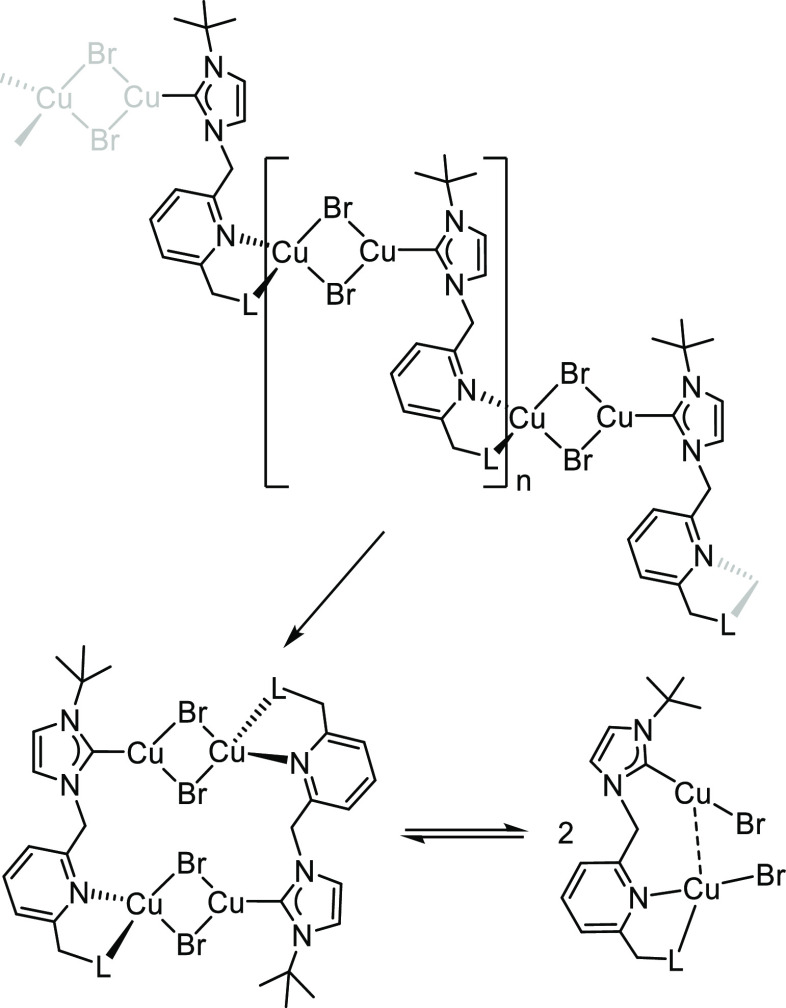
Fragmentation of the Coordination Polymers and Reversible Formation of the Tetranuclear Species in the Solution (L = OMe, NEt_2_, NH^t^Bu)

The high solubility of the aforementioned coordination polymers in conjunction with the simplicity of the NMR spectra as well as the sharp profile of the signals suggests that polymers are prone to fragment in solutions, yielding smaller subunits that likely recombine in a dynamic equilibrium. Fragmentation of the polymer [Cu_2_(μ-Br)_2_(^t^BuImCH_2_pyCH_2_L)]_*n*_ into its constituents should give rise to dinuclear species of composition [(CuBr)_2_(μ-^t^BuImCH_2_pyCH_2_L)] having di- and tri-coordinated Cu(I) centers (Scheme 2).^30^ The high-resolution ESI+ mass spectra of the compounds show both the peak of the metal fragment [L + Cu]^+^ and that of the imidazolium salt (LH^+^). In addition, the fragments [L + CuBr+H]^+^ and [L + Cu_2_ + Br]^+^ were observed in the mass spectrum of **6**. Interestingly, a peak at *m*/*z* 1012.0384 [M-2Br-2H]^+^ derived from a copper tetranuclear species was also observed in the HRMS of **5**.

In this regard, DFT calculations have shown that the related tetranuclear species [Cu_2_(μ-Br)_2_(^t^BuImCH_2_pyCH_2_L)]_2_ are more stable than the dinuclear ones [(CuBr)_2_(μ-^t^BuImCH_2_pyCH_2_L)]. However, the fragmentation process of the tetranuclear species [Cu_2_(μ-Br)_2_(^t^BuImCH_2_pyCH_2_NEt_2_)]_2_ to yield two dinuclear species is affordable, the dinuclear fragments being 6.6 kcal·mol^–1^ higher in energy than the original tetramer. In addition, this process is less unfavorable for **4** (L = OMe) and **6** (L = NH^t^Bu), with Δ*G* being 0.1 and 0.9 kcal·mol^–1^, respectively. Although the coordination mode of each functionalized NHC ligand in the proposed tetranuclear species is identical to that shown in the polymer structure, only two Cu_2_(μ-Br)_2_ moieties are involved, thereby resulting in a compact tetranuclear structure of *C*_2_ symmetry compatible with the NMR spectroscopic data. At this point, it is worth mentioning that Cu(I) complexes having 2-pyridylmethyl-functionalized NHC ligands exhibit a considerable structural diversity ranging from mononuclear^31^ to dinuclear structures^32^ or coordination polymers.^33^ Taking this information into account, we propose that compounds **4**–**6** are coordination polymers in the solid state that fragment in the solution to afford the corresponding tetranuclear species, which are likely in equilibrium with the dinuclear analogs (Scheme 2).

**Figure 4:**
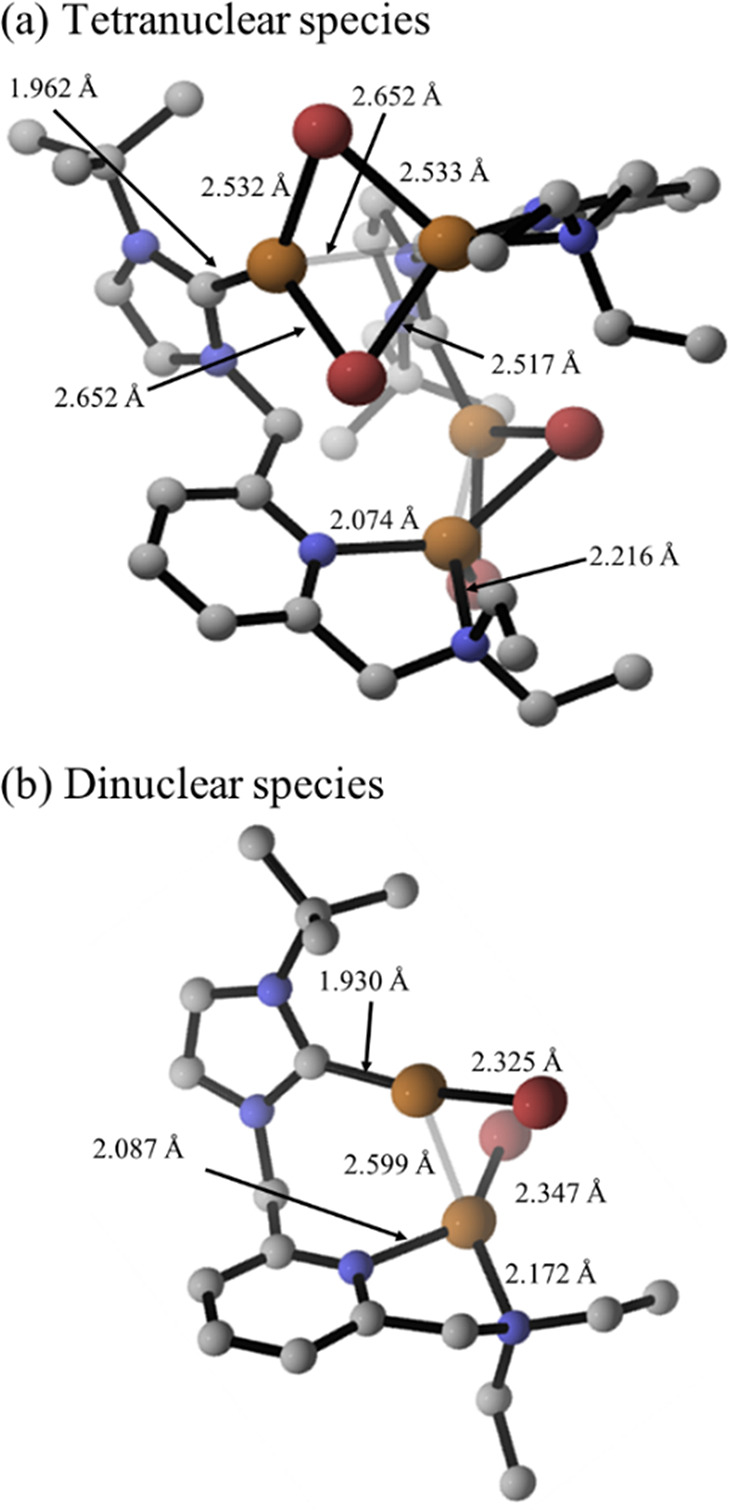
Optimized structure for (a) the tetranuclear compound [Cu_2_(μ-Br)_2_(^t^BuImCH_2_pyCH_2_NEt_2_)]_2_ (**5**) and (b) the dinuclear [(CuBr)_2_(μ-^t^BuImCH_2_pyCH_2_NEt_2_)]. Hydrogen atoms have been omitted for clarity.

In terms of structural parameters, the DFT-computed Cu–Cu distance of the Cu_2_(μ-Br)_2_ moiety in the tetranuclear species resulting from **5** is shorter than that determined crystallographically in the polymer structure of **5**, 2.652 Å vs 2.8185(6) Å (Figure 4a). This is a consequence of the compact structure of the tetranuclear species, which allows for a Cu–Cu bonding interaction. In addition, we found that the dinuclear species also exhibit a bonding interaction with a computed Cu–Cu distance of 2.599 Å (Figure 4b), which may contribute to the stabilization of the structure.

### Azide–Alkyne Cycloaddition Reactions Catalyzed by [Cu_2_(μ-Br)_2_(^t^BuImCH_2_pyCH_2_L)]_2_

[Cu_2_(μ-Br)_2_(^t^BuImCH_2_pyCH_2_L)]_2_ (L = OMe, NEt_2_, NH^t^Bu) are efficient catalysts for the [3 + 2] azide–alkyne cycloaddition reaction that selectively affords the 1,4-regioisomer even at low catalytic loadings. The catalytic activity of the copper(I) polynuclear complexes was first examined for the cycloaddition of benzyl azide to phenylacetylene as a benchmark click reaction. The catalysis was carried out at room temperature under an argon atmosphere, in the absence of a base and solvent, using a catalyst loading of 0.5 mol % (Table 1). Compounds **5** and **6**, featuring an amino functional group, were found to be more active than **4** (L = OMe), which achieves 79% conversion in 5 min (entries 6–8). Quantitative conversion to 1-benzyl-4-phenyl-1*H*-1,2,3-triazole was attained with catalyst **5** (L = NEt_2_) in 5 min (entry 7). Under these conditions, the reaction does not proceed without a catalyst, and when **5** is replaced by CuBr or an equimolecular CuBr/NEt_3_, the reaction efficiency decreases significantly even after prolonged reaction times (entries 1 and 2–5). We also considered acetonitrile as the solvent. However, the **5**-catalyzed cycloaddition reaction is slower, with roughly 70% conversion in 5 min, which increases to 98% after 30 min (entries 9–11). This activity decrease was also observed in dichloromethane and becomes more pronounced in polar solvents such as methanol and water (entries 12–14).

**Table 1:**
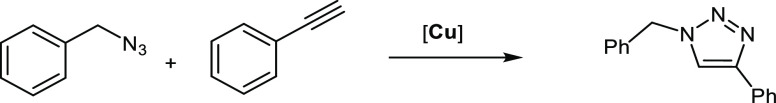
Catalyst Screening and Solvent Optimization for the Cycloaddition of Benzyl Azide and Phenylacetylene^a^

entry	catalyst	solvent^b^	*t* (min)	conversion (%)^c^
1	none	neat	5	0
2	CuBr	neat	5	9
3	CuBr	CH_3_CN	60	27
4	CuBr + NEt_3_ (1:1)	neat	5	21
5	CuBr + NEt_3_ (1:1)	CH_3_CN	60	41
6	**4**	neat	5	79
7	**5**	neat	5	100
8	**6**	neat	5	94
9	**5**	CH_3_CN	5	69
10	**5**	CH_3_CN	20	92
11	**5**	CH_3_CN	30	98
12	**5**	CH_2_Cl_2_	5	68
13	**5**	MeOH	5	51
14	**5**	H_2_O	5	36

a Reaction conditions: benzyl azide (0.5 mmol), phenylacetylene (0.5 mmol), and catalyst CuBr or [Cu_2_(μ-Br)_2_(^t^BuImCH_2_pyCH_2_L)]_2_ (0.0025 mmol, 0.5 mol %) at 298 K.

b Solvent (0.5 mL).

c Conversions, relative to benzyl azide, determined by GC using mesitylene as internal standard.

As for the scope of the cycloaddition reaction, the performance of **5** was investigated by using benzyl azide and phenyl azide, as representative azides, and a variety of aromatic and aliphatic alkynes under optimized catalytic conditions (Table 2). The reactions were carried out at room temperature under argon in the absence of solvent and a catalyst loading of 0.5 mol %. Ring-substituted phenylacetylene derivatives bearing electron-rich substituents at the *para* position (−Me, −OMe, −^*t*^Bu) proceeded efficiently to quantitatively afford the corresponding 1,2,3-triazoles **9b**–**9d** in 5 min. Electron-poor alkynes (−CF_3_) reacted slightly slower, requiring 10 min to achieve complete benzyl azide conversion. Sterically hindered alkynes, such as *o*-substituted phenylacetylene (−OMe), reacted at the same rate as the *m*- and *p*-substituted derivatives and reached full conversion in 5 min. Besides, the functionalized alkyne 2-ethynylpyridine was efficiently transformed into **9h** in 5 min. As expected, aliphatic alkynes are less reactive than aromatic ones. Namely, a 42% conversion was achieved in the cycloaddition reaction of benzyl azide to hex-1-yne in 5 min, although conversion increased to 63% in 30 min, and full conversion to the target 1,2,3-triazole **9i** took 3 h. Similarly, 1,7-octadiyne was efficiently transformed into the corresponding bis-triazole product **9j** in 3 h. On the other hand, internal alkynes such as but-2-yne or diphenylacetylene exhibited much slower reaction rates, affording around 25% conversion to the desired 1,2,3-triazoles **9k** and **9l** in 72 h at 343 K.

**Table 2:**
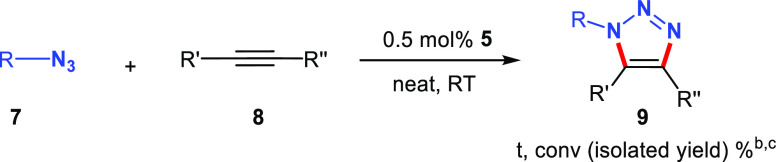

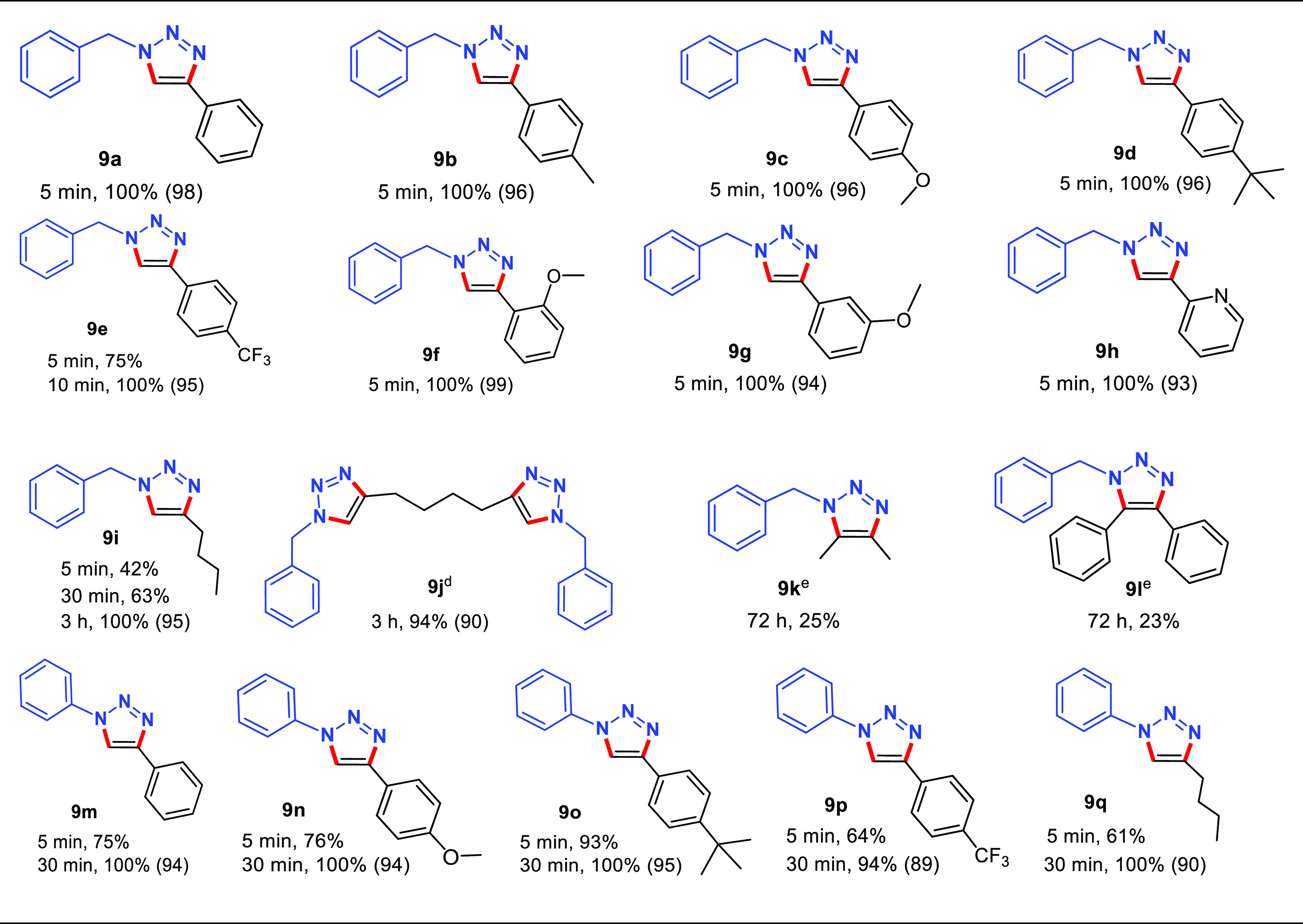
[3 + 2] Cycloaddition of Azides and Alkynes Catalyzed by [Cu_2_(μ-Br)_2_(^t^BuImCH_2_pyCH_2_NEt_2_)]_2_ (**5**)^i^

i Reaction conditions: azide (0.5 mmol), alkyne (0.5 mmol), and catalyst **5** (0.0025 mmol, 0.5 mol %) at 298 K.

b Conversion relative to azide determined by GC using mesitylene as an internal standard.

c Isolated yields in parenthesis.

d Azide (1.0 mmol).

e Temperature 343 K.

The cycloaddition reaction involving the sterically demanding phenyl azide proceeded more slowly than benzyl azide. Even so, ring-substituted phenylacetylene derivatives were efficiently transformed into the corresponding triazoles **9m**–**9p** in 30 min regardless of the electronic character of the substituent at the *para* position. Gratifyingly, hex-1-yne produced quantitatively **9q** in only 30 min. The increased reaction rate compared to benzyl azide can be tentatively attributed to the electron-delocalization enabled by the azide, which allows overcoming the steric penalty.

The 1,4-disubstituted-1,2,3-triazole derivatives **9** were isolated as solids in excellent yields for any azide–alkyne combination, typically 90–95%, by washing the crude product with pentane.

### Azide–Alkyne Cycloaddition Reactions Catalyzed by [Cu_2_(μ-Br)_2_(^t^BuImCH_2_pyCH_2_NEt_2_)]_2_ (**5**) at Low Catalyst Loading

The excellent catalytic activity of **5** at a relatively low catalyst loading (0.5 mol %) prompted us to explore the possibility of decreasing it even more. This way, the performance of **5** was investigated using benzyl azide and phenylacetylene as model substrates at lower catalyst loading under neat conditions (Table 3).

**Table 3:**
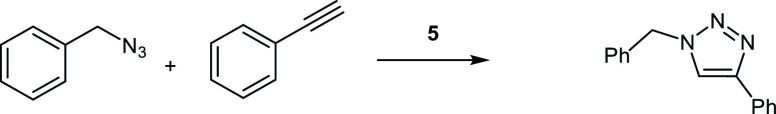
Cycloaddition of Benzyl Azide and Phenylacetylene Catalyzed by [Cu_2_(μ-Br)_2_(^t^BuImCH_2_pyCH_2_NEt_2_)]_2_ (**5**) at Low Catalyst Loading^a^

entry	*T* (K)	solvent	**5** (mol %)	*t* (h)	conversion (%)^b^	TON	TOF (h^–1^)
1	298	neat	0.25	0.08	56	224	2800
2	298	neat	0.05	0.5	50	1000	2000
3	298	neat	0.05	3	88	1760	600
4	298	neat	0.005	24	96	19,200	800
5	323	neat	0.005	24	99	19,800	820
6	298	CH_3_CN	0.005	24	0	0	0
7	323	CH_3_CN	0.005	48	80	16,000	300
8	298	neat	0.0025	24	66	26,400	1100
9	298	neat	0.0025	48	82	32,800	680
10	323	neat	0.0025	24	90	36,000	1500
11	323	neat	0.0025	48	98	39,200	820

a Reaction conditions: benzyl azide (0.5 mmol), phenylacetylene (0.5 mmol), solvent (0.5 mL).

b Conversion relative to benzyl azide and selectivities determined by GC using mesitylene as internal standard.

**Figure 5:**
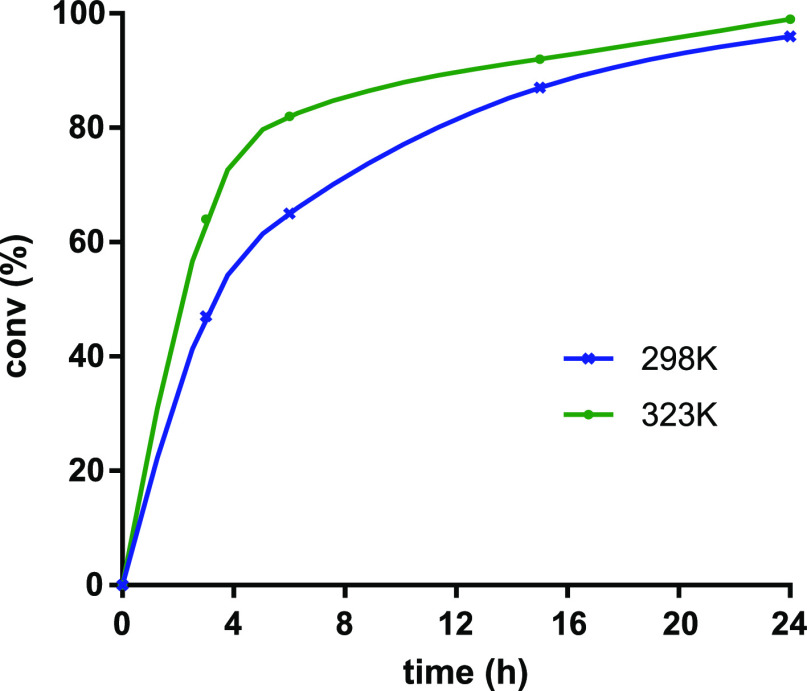
Reaction profile of conversion *vs* time for the cycloaddition of neat benzyl azide (0.5 mmol) and phenylacetylene (0.5 mmol) catalyzed by **5** (0.005 mol %) at 298 and 323 K.

Decreasing the catalyst loading to 0.25 mol % resulted in a conversion of phenyl azide of 56% at 298 K (entry 1). Similar conversion was attained after 0.5 h by decreasing the catalyst loading to 0.05 mol %, which increased to 88% after 3 h (entries 2 and 3). Further decreasing the catalyst loading to 0.005 mol % (50 ppm) gave 96% conversion in 24 h at 298 K. Increasing the temperature to 323 K allowed to quantitatively afford triazole **9a** in 24 h (TOF = 820 h^–1^) (entries 4 and 5). The reaction profile (conversion vs time) for the cycloaddition of benzyl azide to phenylacetylene catalyzed by **5** (0.005 mol %) at 298 and 323 K is shown in Figure 5. The process is faster at 323 K at the early reaction stage, although similar reaction times are required to achieve complete conversion of benzyl azide. Although no reaction was observed in acetonitrile at 298 K at the same catalyst loading, 80% conversion was attained at 323 K after 48 h (entries 6 and 7). Remarkably, it is possible to decrease the catalyst loading up to 0.0025 mol % (25 ppm) working under neat conditions at reasonable reaction times. At 298 K, a benzyl azide conversion of 82% was achieved in 48 h, increasing to 90% (24 h, TOF = 1500 h^–1^) and 98% (48 h, TOF = 820 h^–1^) at 323 K (entries 8–11).

Catalytic studies performed at low catalyst loading further highlight the efficiency of catalyst **5** (Table 4). CuAAC of benzyl azide with *para*-substituted phenylacetylene derivatives proceeded efficiently at 0.005 mol % (50 ppm) at room temperature regardless of the electronic character of the substituent (−OMe and −CF_3_), with conversions to **9c** and **9e** higher than 90% in 24 h. Similar conversion was attained when the reactions were performed at 0.0025 mol % (25 ppm) at 323 K. Interestingly, the formation of the 1,5-disubstituted-1,2,3-triazole regioisomers was not observed in any case under these conditions. Unfortunately, aliphatic alkynes such as hex-1-yne were not transformed under low catalyst loading conditions even at 323 K.

**Table 4:**
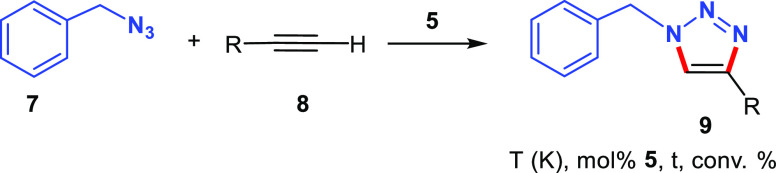

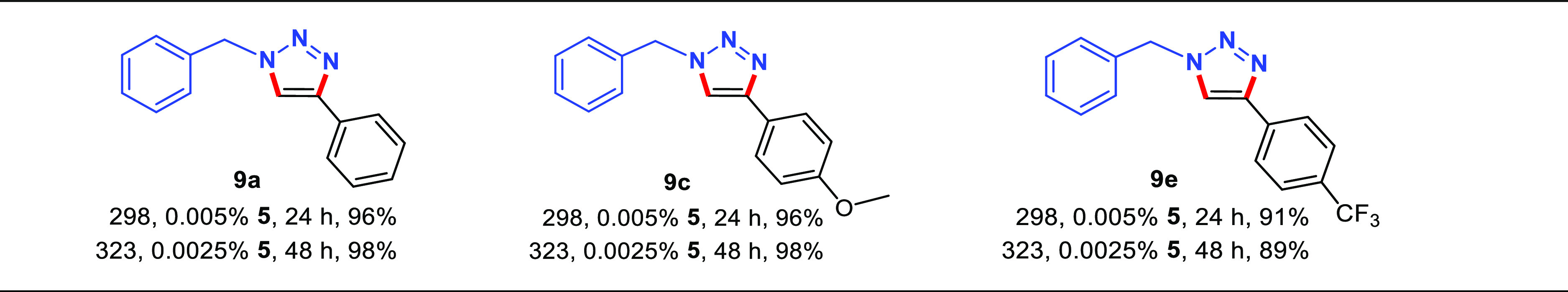
Cycloaddition of Benzyl Azide and Alkynes Catalyzed by [Cu_2_(μ-Br)_2_(^t^BuImCH_2_pyCH_2_NEt_2_)]_2_ (**5**) at Low Catalyst Loading^a^^,^^b^

a Reaction conditions: benzyl azide (0.5 mmol) and alkyne (0.5 mmol).

b Conversion relative to benzyl azide determined by GC using mesitylene as internal standard.

### Mechanistic Insights on the [3 + 2] Azide–Alkyne Cycloaddition Reaction Catalyzed by [Cu_2_(μ-Br)_2_(^t^BuImCH_2_pyCH_2_NEt_2_)]_2_ (**5**)

To ascertain the reaction mechanism of the azide–alkyne cycloaddition catalyzed by **5** and attain an in-depth understanding of the origin of its high activity and selectivity, we performed a series of reactivity experiments and a DFT-based theoretical study.

### Reactivity Studies

First, we studied the cycloaddition reaction by NMR using a high catalyst loading. Reaction of benzyl azide and phenylacetylene (20 equiv, 1:1 molar ratio) catalyzed by **5** (2.5 mol %) in CD_3_CN at 298 K resulted in the formation of the cycloaddition reaction product 1-benzyl-4-phenyl-1*H*-1,2,3-triazole in 5 min. In addition, the ^1^H NMR of the reaction mixture showed the disappearance of **5** and the formation of a new species, **10**. It shows a new set of resonances for the lutidine-derived polydentate ligand, including a downfield resonance at δ 9.19 ppm suggesting the presence of an imidazolium fragment in the compound. Remarkably, the addition of a new load of benzyl azide and phenylacetylene (20 equiv) to the solution afforded the 1,2,3-triazole reaction product within a few minutes, thus suggesting that **10** is catalytically competent for the cycloaddition reaction.

**Scheme 3:**
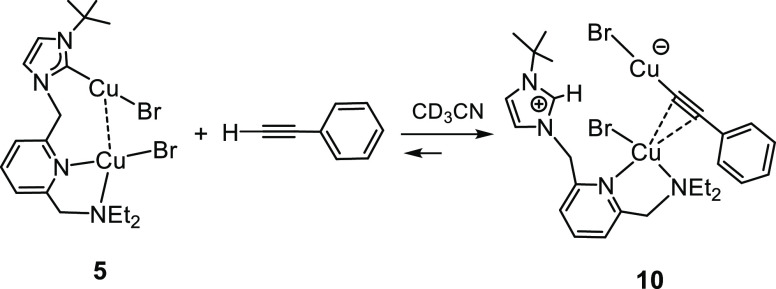
Reaction of **5** with Phenylacetylene: Formation of [(CuBr)_2_(C≡CPh)(^t^BuHImCH_2_pyCH_2_NEt_2_)] (**10**) (Structure Based on DFT Calculations)

Compound **10** was formed immediately by reaction of **5** with a moderate excess of phenylacetylene (1.2 equiv relative to the dinuclear derivative of **5**) in CD_3_CN. However, NMR spectra of the reaction mixture at 243 K evidenced the presence of unreacted **5** and phenylacetylene. The amount of **10** in the reaction mixture increased upon increasing the amount of phenylacetylene added (5 equiv), which eventually made it possible to record the ^13^C{^1^H}-APT NMR spectrum (see the Supporting Information). Compound **10** has been identified as a zwitterionic copper(I) dinuclear alkynyl species [(CuBr)_2_(C≡CPh)(^t^BuHImCH_2_pyCH_2_NEt_2_)] bearing the functionalized imidazolium ligand. The proposed structure for **10** (shown in Scheme 3) is based on DFT calculations (see below) and the experimental evidence. Namely, the existence of an imidazolium group in **10** was confirmed by bidimensional HSQC and HMBC spectra. The broad singlet at δ 9.19 ppm, corresponding to the acidic imidazolium proton NCHN, correlates with the CH signal at δ 139.2 ppm in the ^13^C{^1^H}-APT NMR spectra. Moreover, the long-range ^1^H–^13^C HMBC spectrum showed cross-peaks between the NCHN proton and the =CH carbons of imidazole at δ 124.1 and 121.0 ppm. In addition, the ^13^C NMR spectra showed two resonances at δ 126.7 and 98.8 ppm that are assigned to the C_α_ and C_β_ atoms of the alkynyl ligand, respectively.^19,20^

In short, intermediate **10** is the result of the deprotonation of phenylacetylene mediated by the imidazole-2-ylidene scaffold, which leads to the formation of an imidazolium-alkynyl species. Although it might seem somewhat counterintuitive, this kind of result is not unprecedented, as the formation of alkynyl intermediates by protonation of the NHC groups dissociated from the Cu atom has already been reported in several CuAAC processes.^10a,10,25d^

To shed more light on the reaction mechanism, we complemented the previous studies with deuterium labeling experiments. In this regard, treatment of benzylazide (0.5 mmol) with phenylacetylene-*d*_1_ (0.5 mmol) in the presence of a solution of catalyst **5** (0.025 mol %) in CD_3_CN (0.5 mL) resulted in the formation of the 1,2,3-triazole after 5 h at room temperature. Importantly, the position 5 of the N-heterocyclic ring was fully deuterated, with no deuteration observed at any other position.

### Theoretical Study on the [3 + 2] Azide–Alkyne Cycloaddition Reaction Catalyzed by **5**

We have found experimental and theoretical evidence that coordination polymer **5** likely breaks down into tetranuclear [Cu_2_(μ-Br)_2_(^t^BuImCH_2_pyCH_2_NEt_2_)]_2_ species. In addition, fragmentation of the tetranuclear structure into the dinuclear species is an affordable process, which is favored from an entropic point of view, as once the tetramer breaks apart, the feasibility of a molecular encounter that regenerates the original structure is very low under catalytic conditions. Thus, we hypothesized that the dinuclear derivative of complex **5** (see Scheme 2) is the active species for the catalytic cycle and computed the reaction profile on the basis of this structure. In addition, as previously explained, activation of the catalyst precursor **5** involves the reaction with phenylacetylene, leading to protonation of the NHC moiety. The resulting alkynyl compound **10** corresponds to a zwitterionic copper(I) dinuclear complex with a functionalized imidazolium ligand, as discussed in the previous subsection and shown in Scheme 3 (**10** is named as **C** in the reaction mechanism), which arises in a direct way in the proposed reaction mechanism. Notice that we selected phenylacetylene and benzyl azide as models for the alkyne and the azide, respectively. For the sake of clarity, the reaction intermediates and transition states are referred to by capital letters starting with **A** (which corresponds to the dinuclear derivative of **5**).

**Figure 6:**
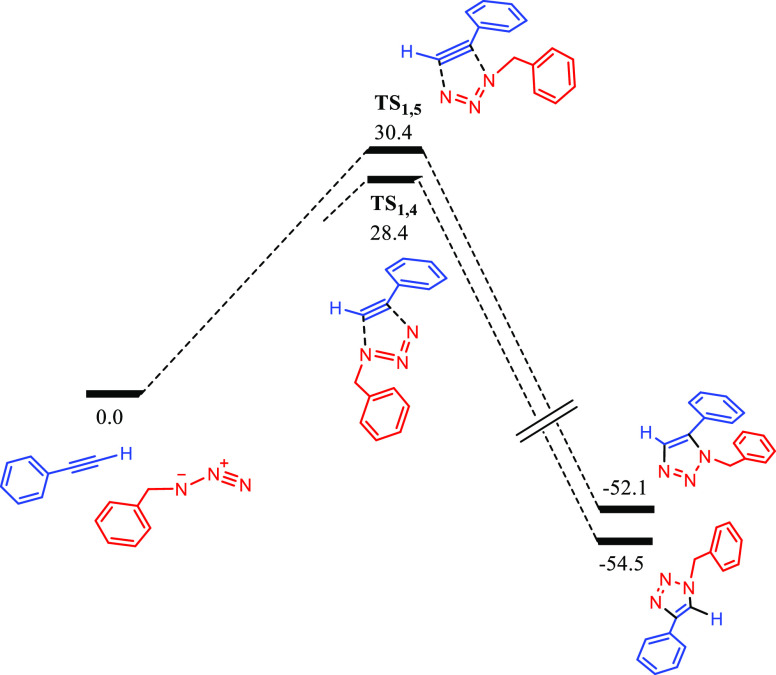
Gibbs energy profile (kcal·mol^–1^) for the noncatalyzed Huisgen 1,3-dipolar cycloaddition between phenylacetylene and benzyl azide.

As an initial step, we computed the reaction profile for the classical, noncatalyzed, Huisgen 1,3-dipolar cycloaddition reaction, which is known to yield a mixture of the corresponding 1,4- and 1,5-substituted 1,2,3-triazoles.^3^ This general result is consistent with our calculations, as the Gibbs energy difference between the transition structures that lead to the 1,4- and 1,5-regiosiomers is only 2.0 kcal·mol^–1^, thus explaining the lack of selectivity of the process (see Figure 6). In addition, the energy barrier would be 28.4 kcal·mol^–1^ (for the 1,4-disubstituted-1,2,3-triazole), which is too high for the reaction to take place at room temperature.

**Figure 7:**
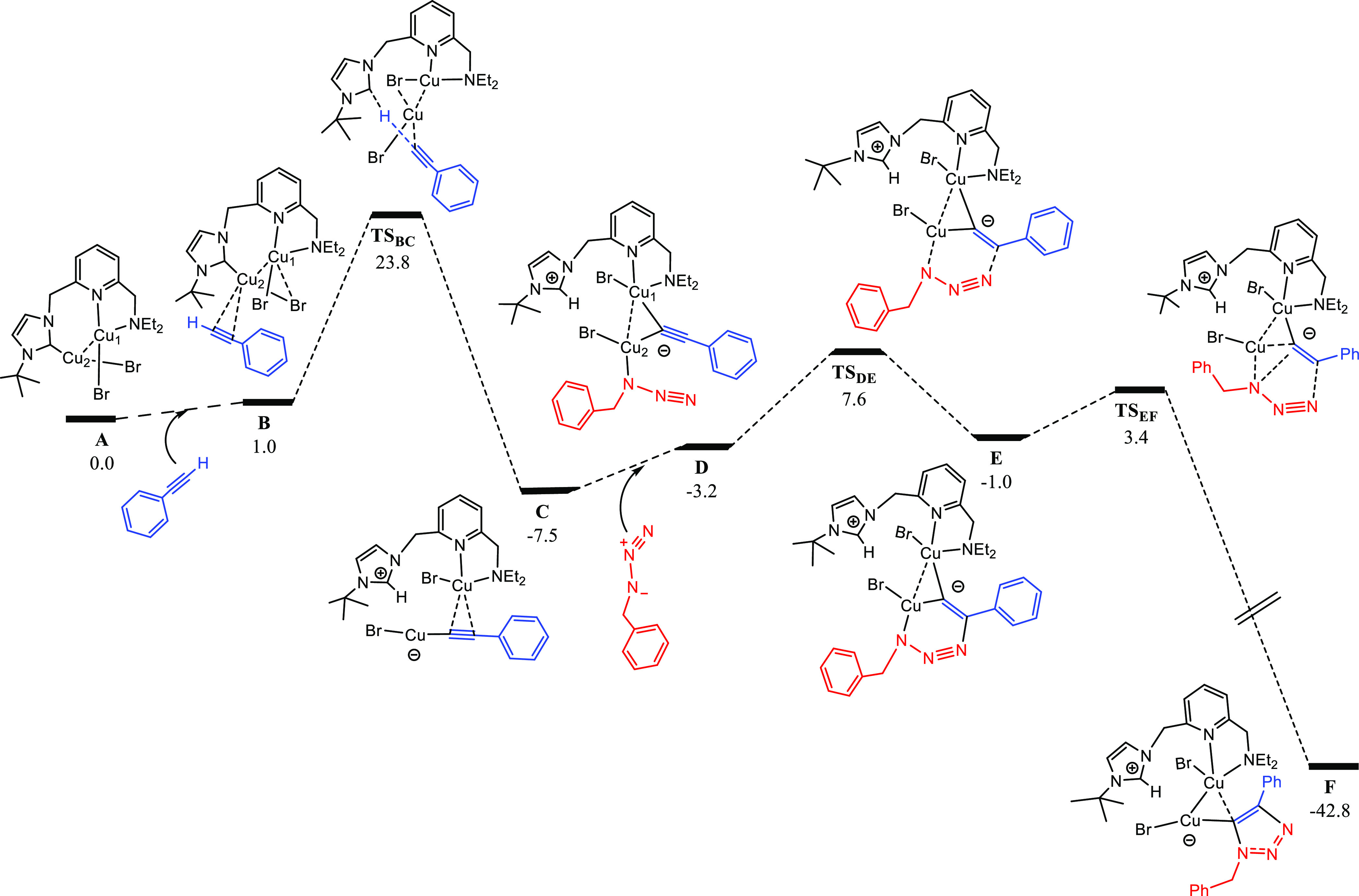
DFT calculated Gibbs energy reaction profile (in kcal·mol^–1^ relative to **A** and isolated molecules) for the alkyne activation and triazolyl scaffold formation in the CuAAC reaction catalyzed by **A**. The labeling used for Cu atoms in the main text is shown in structure **A**.

**Figure 8:**
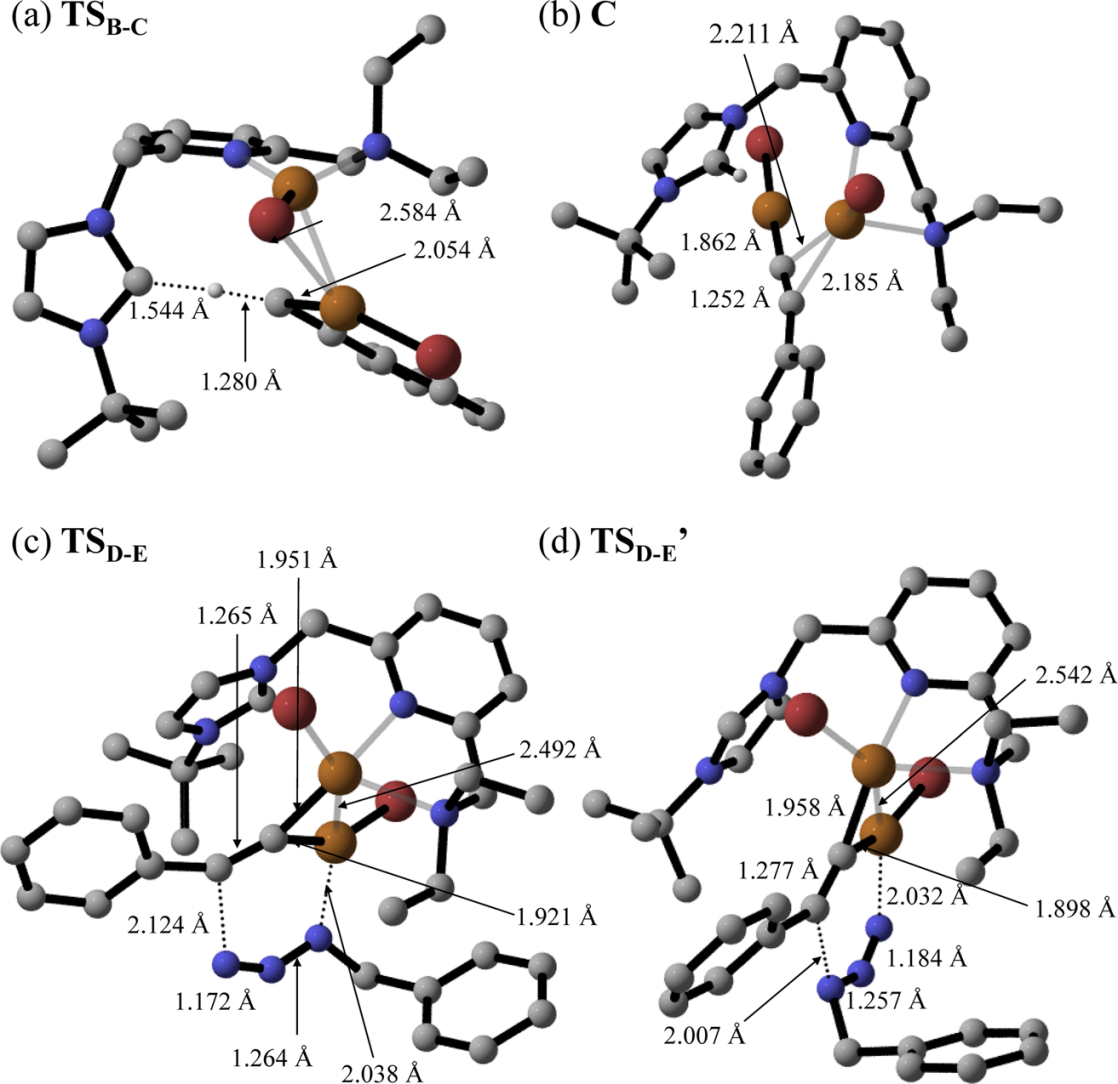
DFT-optimized structures and selected distances of (a) **TS_B-C_**, (b) **C**, (c) **TS_D-E_**, and (d) **TS_D-E_′**. Nonrelevant hydrogen atoms have been omitted for clarity.

**Figure 9:**
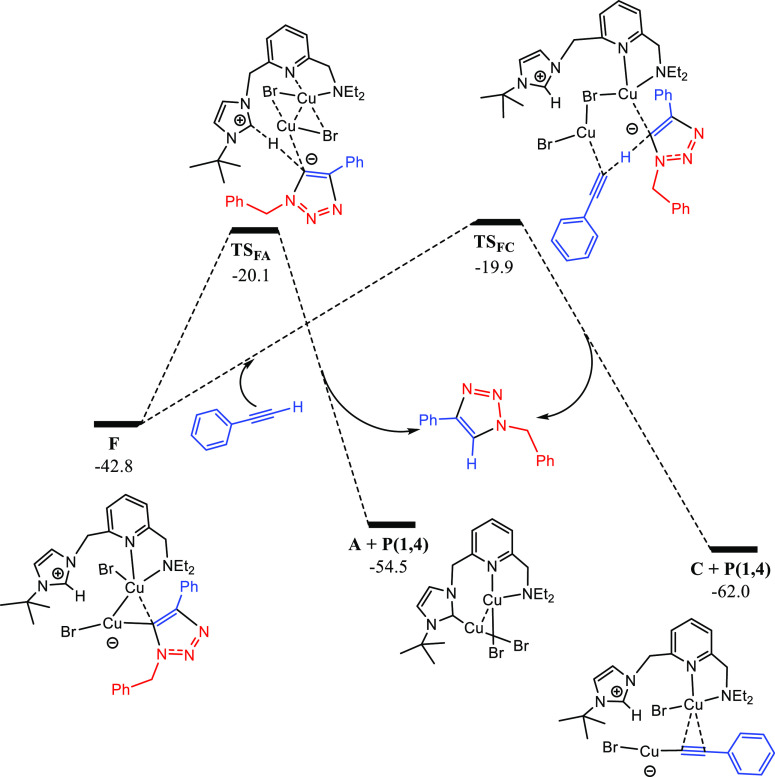
DFT calculated Gibbs energy reaction profile (in kcal·mol^–1^ relative to **A** and isolated molecules) for the 1,4-disubstituted 1,2,3-triazole product formation in the CuAAC reaction catalyzed by **A**.

The Gibbs energy landscape obtained for the CuAAC reaction catalyzed by **A** along with selected calculated transition state structures is shown in Figures 7–9. As depicted in Figure 7, the first step involves the π-coordination of the alkyne to the Cu2 center—notice that we have used the same labeling as for the crystal structure—leading to intermediate **B**, which is 1.0 kcal·mol^–1^ higher in energy. As expected, this coordination leads to a subtle increase in the Cu–Cu distance from 2.599 Å (in **A**) to 2.674 Å (in **B**). The next step consists of the alkyne deprotonation by the NHC moiety via **TS_B-C_**, yielding the zwitterionic intermediate **C** (see Figure 8a,b), which we find to be consistent with the experimentally characterized species **10**. Notice that the structure of **C** corresponds to a σ,π-bis(copper)acetylide complex, which has extensively been reported in CuAAC processes.^20^ The process has an effective energy barrier of 23.8 kcal·mol^–1^ (dictated by the energy difference between **TS_B-C_** and **A**), which is affordable under the reaction conditions, especially considering that the catalytic reaction takes place in neat phenylacetylene. It is also remarkable that **C** is 7.5 kcal·mol^–1^ more stable than **A**, which agrees with the experimental observation of the formation of **10** by the reaction of **5** with phenylacetylene.

Then, the azide coordinates to the Cu atom bonded to the alkynyl moiety,^23f^ yielding intermediate **D**, with a relative Gibbs energy of −3.2 kcal·mol^–1^. As a result, the alkynyl ligand changes its coordination mode to μ^2^-η^1^ and the Cu–Cu distance decreases from 3.016 Å (in **C**) to 2.508 Å (in **D**). The next step involves the cycloaddition between the azide and the alkyne, which is mediated by the Cu active center, forming the six-membered metallacycle **E**. This step proceeds via **TS_D-E_** and determines the regioselectivity of the process, as the relative orientation of the azide and the alkyne within the transition structure determines whether the 1,4- or 1,5-disubstituted 1,2,3-triazole product will form. In this regard, the transition structure leading to the 1,4-substituted triazole, **TS_D-E_**, is 8.9 kcal·mol^–1^ lower in energy than that leading to the 1,5-product, **TS_D-E_′**, and this result explains the complete process regioselectivity (see Figure 8c,d). Thereby, the first C–N bond is formed, overcoming an effective energy span of 15.1 kcal·mol^–1^ (with respect to **C**), thus being affordable at the reaction conditions. As expected, both Cu atoms actively participate in this step, as highlighted by Cu–C distances of 1.921 and 1.951 Å, as well as a Cu–Cu distance of 2.492 Å (Figure 8c), which are similar to those reported by other authors for related CuAAC processes.^23b^ As previously introduced, **TS_D-E_** leads to the formation of Cu-based metallacycle **E**, with a relative energy of −1.0 kcal·mol^–1^ and in which both Cu atoms are bonded to the C atom at position 5 (as shown by Cu–C distances of 1.868 and 1.925 Å).

The following reaction step involves the contraction of the six-membered metallacycle **E** through the formation of the second C–N bond to yield the Cu-triazolyl species **F**. This process takes place via **TS_E-F_**, bearing an effective energy barrier of 10.9 kcal·mol^–1^ (with respect to **C**), and is highly exergonic, as **F** is 41.8 kcal·mol^–1^ more stable than **E**, having a relative Gibbs energy of −42.8 kcal·mol^–1^. Also note that the Cu–Cu distance in **F** is 2.470 Å, indicative of a strong cuprophilic interaction between both metal centers. In addition, the triazolyl moiety is coordinated in a bridging fashion to both Cu atoms, with Cu–C distances of 1.959 and 2.115 Å, in line with DFT calculations reported by other authors.^25c^

As a final step, the 1,4-disubstituted-1,2,3-triazole product is released, which might proceed by two different, although related, reaction pathways (see Figure 9). The first possibility consists in the proton transfer from the protonated NHC scaffold to the triazolyl moiety in **F**, via **TS_F-A_**, which leads to the final product and recovers the initial dinuclear structure (**A**). This pathway requires to overcome an energy barrier of 22.7 kcal·mol^–1^, determined by the Gibbs energy difference between **TS_F-A_** and intermediate **F**. Alternatively, the triazolyl fragment could be protonated by an additional phenylacetylene molecule, also releasing the final product and recovering intermediate **C** (which has been detected experimentally). This second route bears an energy barrier of 22.9 kcal·mol^–1^ (Gibbs energy difference between **TS_F-C_** and **C**), which is only 0.2 kcal·mol^–1^ higher than that involving **TS_F-A_**. Hence, both possibilities are expected to be operative under the reaction conditions.

Overall, the calculated effective energy span (Δ*G*^⧧^) for the catalytic cycle in terms of the framework proposed by Kozuch and Shaik is 23.8 kcal·mol^–1^,^34^ the rate-determining step (RDS) being the alkyne deprotonation of the initial complex (A) to afford Cu(I) acetylide species **C** via **TS_B-C_**. At this point, it should be noted that if the final reaction step proceeds through **TS_F-C_** instead of **TS_F-A_** (whose relative energy difference is only 0.2 kcal·mol^–1^), the subsequent reaction cycle would start directly in **C**, avoiding the need for overcoming **TS_B-C_** again. In this situation, the RDS would be the final protonation of the triazolyl moiety (which also implies the alkyne activation). This corresponds to a very similar process, and the effective energy span would be 22.9 kcal·mol^–1^ (determined by **TS_F-C_**). It is remarkable that some authors have proposed the C–N formation as the RDS,^10b,16c,23b,24b,35^ while others propose that the RDS corresponds to alkyne deprotonation processes.^20,36,37^ In this regard, our results are in line with the latter observations, although it should be considered that the medium acidity will play a crucial role in this difference.^16d^ In this regard, it is worth mentioning that some reports indicate that substrate selection may switch the RDS step,^35,36a,38^ which provides additional evidence of the possibility of switching between both alternatives. At this point, it is also remarkable to note that we found the azide to coordinate to the σ-alkynyl bound Cu atom (Cu2 in Figure 7) instead of to the typically proposed ligand-bound Cu center (Cu1 in Figure 7), in agreement with reports from other authors.^23f,25d^

According to the reaction mechanism proposed on the basis of DFT calculations, the deprotonation of the alkyne in the RDS of both reaction pathways should lead to the observation of a primary deuterium kinetic isotope effect. However, the KIE (*k*_H_/*k*_D_) of 0.97 ± 0.01 determined in acetonitrile (see the Supporting Information) suggests that the solvent might be involved in the mechanism. In this regard, we note that the cycloaddition reaction is much slower in acetonitrile than under neat conditions (see Table 1), and a significant dependency of the reaction kinetics due to solvent effects for these kinds of processes has already been reported.^36a^

This way, we expect that solvent effects might be responsible for the lack of agreement between DFT and experimental results, without excluding other potential processes that may take place in the reaction medium and are likely to affect the reaction mechanism.

## Conclusions

Copper(I) [Cu_2_(μ-Br)_2_(^t^BuImCH_2_pyCH_2_L)]_*n*_ (L = OMe, NEt_2_, NH^t^Bu) compounds have been prepared from the corresponding functionalized imidazolium salt by reacting the in situ generated Ag-NHC complexes with copper powder. The compounds are coordination polymers in the solid state that very likely break down in solutions into tetranuclear [Cu_2_(μ-Br)_2_(^t^BuImCH_2_pyCH_2_L)]_2_ species in equilibrium with dinuclear ones. DFT calculations have shown that the fragmentation of the tetranuclear compounds into dinuclear [(CuBr)_2_(μ-^t^BuImCH_2_pyCH_2_L)] species is an affordable process. These compounds have been proven to be efficient catalysts in the base-free [3 + 2] cycloaddition reactions of azides and alkynes at room temperature under an inert atmosphere without solvent and a catalyst loading of 0.5 mol %, with compound [Cu_2_(μ-Br)_2_(^t^BuImCH_2_pyCH_2_NEt_2_)]_2_ being the most active. This system efficiently catalyzes the cycloaddition reaction of benzyl azide and phenyl azide for a wide range of terminal alkynes with different properties to quantitatively afford the corresponding 1,4-disubstituted 1,2,3 triazole derivatives in a few minutes. The cycloaddition reaction of benzyl azide to phenylacetylene can be performed with a catalyst loading of 25–50 ppm by increasing the reaction time and/or temperature.

Reactivity studies have shown that the activation of the catalyst precursor [Cu_2_(μ-Br)_2_(^t^BuImCH_2_pyCH_2_NEt_2_)]_2_ involves the alkyne deprotonation by the NHC moiety of the polydentate ligand to afford a copper(I)-alkynyl species bearing a functionalized imidazolium ligand. We have explored a possible reaction mechanism involving the dinuclear compound [Cu_2_(μ-Br)_2_(^t^BuImCH_2_pyCH_2_NEt_2_)] as the catalytic active species or the active catalyst. DFT calculations have shown that the reaction proceeds through zwitterionic dinuclear intermediates with participation of both copper atoms following, in general terms, a Fokin mechanism. Nonetheless, there is a notable difference with respect to the original proposal, as the azide coordinates to the copper atom bonded to the alkynyl moiety rather than to the ligand-coordinated Cu center. The DFT results point to the RDS being the alkyne activation, via deprotonation by the NHC moiety, which is a consequence of the lability of the Cu-NHC bond and the basicity of the carbene. In addition, two possibilities that are very close in energy were identified for the last reaction step (triazolyl moiety protonation). Namely, it can proceed by proton transfer from the imidazolium moiety or from a phenylacetylene molecule, both being operative in the reaction conditions.

## Experimental Section

### General Considerations

All the experimental procedures were performed under an argon atmosphere using Schlenk or glovebox techniques. Solvents were distilled immediately prior to use from the appropriate drying agents or obtained from a Solvent Purification System (Innovative Technologies). Oxygen-free solvents were employed throughout. CDCl_3_ and CD_3_CN were dried using activated molecular sieves and degassed by three freeze–pump–thaw cycles. The functionalized lutidine-derived imidazolium salts [^t^BuHImCH_2_pyCH_2_Br]Br and [^t^BuHImCH_2_pyCH_2_OMe]Br (**1**) were prepared following the procedure recently reported by us.^26^ Phenyl azide^39^ and benzyl azide^40^ were prepared according to methods described in the literature. The organic substrates were obtained from common commercial sources and used as received or distilled prior to use depending on their purity.

### Scientific Equipment

C, H, and N analyses were carried out in a PerkinElmer 2400 Series II CHNS/O analyzer. ^1^H and ^13^C{^1^H} NMR spectra were recorded on a Bruker Avance 300 (300.1276 and 75.4792 MHz). NMR chemical shifts are reported in ppm relative to tetramethylsilane and are referenced to partially deuterated solvent resonances. Coupling constants (*J*) are given in hertz. Spectral assignments were achieved by combination of ^1^H–^1^H COSY, ^13^C APT, ^1^H–^13^C HSQC, and ^1^H–^13^C HMBC experiments. High-resolution electrospray ionization mass spectra (HRMS-ESI) were recorded on a Bruker MicroToF-Q equipped with an API-ESI source and a Q-ToF mass analyzer, which leads to a maximum error in the measurement of 5 ppm, using sodium formate as reference. The catalytic reactions were analyzed on an Agilent 4890D system equipped with an HP-INNOWax capillary column (0.4 μm film thickness, 25 m × 0.2 mm i.d.) using mesitylene as internal standard. Organic compounds were identified by gas chromatography–mass spectrometry (GC/MS) using an Agilent 6890 GC system with an Agilent 5973 MS detector equipped with an HP-5MS polar capillary column (0.25 μm film thickness, 30 m × 0.25 mm i.d.).

### Synthesis of Functionalized Imidazolium Salts and Copper(I) Complexes: Synthesis of [^t^BuHImCH_2_pyCH_2_NEt_2_]Br (**2**)

HNEt_2_ (468 μL, ρ = 0.707 g mL^–1^, 4.523 mmol) and K_2_CO_3_ (2.814 g, 20.360 mmol) were added to a solution of [^t^BuHImCH_2_pyCH_2_Br]Br (1.600 g, 4.112 mmol) in acetonitrile (10 mL), and the mixture was stirred for 60 h at room temperature. The resulting suspension was brought to dryness under a vacuum, and the residue was extracted with dichloromethane (10 mL) to give a suspension that was filtered and washed with dichloromethane (2 × 5 mL). The solution was brought to dryness under a vacuum to afford a pale-yellow oil that was disaggregated by stirring with cold diethyl ether. The obtained yellow solid was washed with diethyl ether (2 × 5 mL) and dried under a vacuum. Yield: 1.241 g, 79%. Anal. calcd for C_18_H_29_BrN_4_: C, 56.69; H, 7.66; N, 14.69. Found: C, 56.08; H, 7.85; N, 13.74. HRMS (ESI+, MeOH, *m*/*z*): calcd for C_18_H_29_N_4_ [M]^+^: 301.2392, found: 301.2399. ^1^H NMR (298 K, 300 MHz, CDCl_3_): δ 11.03 (s, 1H, NCHN), 7.78 (d, *J*_H-H_ = 7.5 Hz, 1H, H_m_ py), 7.72–7.65 (m, 2H, H_p_ py, =CH Im), 7.45 (d, *J*_H-H_ = 7.7 Hz, 1H, H_m_ py), 7.23 (s, 1H, =CH Im), 5.84 (s, 2H, CH_2_Im), 3.67 (s, 2H, CH_2_NEt_2_), 2.54 (q, *J*_H-H_ = 7.1 Hz, 4H, CH_2_ Et), 1.72 (s, 9H, ^t^Bu), 1.03 (t, *J*_H-H_ = 7.1 Hz, 6H, CH_3_ Et). ^13^C{^1^H} NMR (298 K, 75 MHz, CDCl_3_): δ 161.1, 151.8 (C_q_ py), 137.9 (C_p_ py), 136.0 (N*C*HN), 123.3 (C_m_ py), 122.8 (=CH Im), 122.4 (C_m_ py), 118.9 (=CH Im), 60.5 (C ^t^Bu), 59.2 (CH_2_NEt_2_), 53.9 (CH_2_Im), 47.4 (CH_2_ Et), 30.2 (CH_3_^t^Bu), 12.0 (CH_3_ Et).

### Synthesis of [^t^BuHImCH_2_pyCH_2_NH^t^Bu]Br (**3**)

A thick glass reaction tube fitted with a greaseless high-vacuum stopcock was charged with [^t^BuHImCH_2_pyCH_2_Br]Br (1.000 g, 2.570 mmol), ^t^BuNH_2_ (6 mL), and acetonitrile (3 mL). The reaction mixture was stirred for 15 h at 373 K to give a light brown solution. The solution was transferred to a Schlenk tube and brought to dryness under a vacuum to give a solid residue that was dried under a vacuum at 373 K for 4 h. The pale-brown solid was washed with diethyl ether (3 × 10 mL) and dried under a vacuum. Yield: 931 mg, 95%. Anal. calcd for C_18_H_29_BrN_4_: C, 56.69; H, 7.66; N, 14.69. Found: C, 56.61; H, 7.43; N, 14.44. HRMS (ESI+, MeOH, *m*/*z*): calcd for C_18_H_29_N_4_ [M]^+^: 301.2392, found: 301.2380. ^1^H NMR (298 K, 300 MHz, CDCl_3_): δ 10.50 (s, 1H, NCHN), 7.78 (t, *J*_H-H_ = 1.7 Hz, 1H, =CH Im), 7.72–7.60 (m, 2H, H_p_ py, H_m_ py), 7.35–7.27 (m, 2H, H_p_ py, =CH Im), 5.72 (s, 2H, CH_2_Im), 4.03 (s, 2H, C*H_2_*NH^t^Bu), 1.71 (s, 9H, ^t^BuIm), 1.69 (s, 1H, NH), 1.38 (s, 9H, ^t^BuNH). ^13^C{^1^H} NMR (298 K, 75 MHz, CDCl_3_): δ 156.4, 151.9 (C_q_ py), 138.3 (C_p_ py), 137.9 (N*C*HN), 123.3 (=CH Im), 122.8, 122.0 (C_m_ py), 118.8 (=CH Im), 60.5 (C ^t^BuIm), 54.4 (C ^t^BuNH), 53.7 (CH_2_Im), 46.7 (CH_2_NH^t^Bu), 30.3 (CH_3_^t^BuIm), 27.8 (CH_3_^t^BuNH).

### Synthesis of [Cu_2_(μ-Br)_2_(^t^BuImCH_2_pyCH_2_L)]_*n*_ (L = OMe, NEt_2_, NH^t^Bu)

#### General Method

Ag_2_O and copper powder were added to a solution of [^t^BuHImCH_2_pyCH_2_L]Br (L = OMe, NEt_2_, NH^t^Bu) in acetonitrile (5 mL). The suspension was stirred for 30 h at 323 K and then filtered through Celite. The resulting solution was brought to dryness under a vacuum to give an oily residue that was disaggregated by stirring with cold diethyl ether. The solid was filtered, washed with diethyl ether (2 × 5 mL), and dried under a vacuum.

### [Cu_2_(μ-Br)_2_(^t^BuImCH_2_pyCH_2_OMe)]_*n*_ (**4**)

[^t^BuHImCH_2_pyCH_2_OMe]Br (**1**) (200 mg, 0.588 mmol), Ag_2_O (286 mg, 1.235 mmol), and Cu powder (224 mg, 3.528 mmol). Yield: 170 mg, 53% (light brown solid). Anal. calcd for C_15_H_21_Br_2_N_3_OCu_2_: 32.98 C, 3.87 H, 7.69 N. Found: 32.90 C, 3.66 H, 7.79 N. HRMS (ESI+, CH_3_CN, *m*/*z*): calcd for C_15_H_21_N_3_OCu [M-Cu-2Br]^+^: 322.0981, found: 322.0946; calcd for C_15_H_22_N_3_O [M-2Cu-2Br + H]^+^: 260.1763, found: 260.1704. ^1^H NMR (298 K, 300 MHz, CD_3_CN): δ 7.75 (t, *J*_H-H_ = 7.7 Hz, 1H, H_p_ py), 7.36 (d, *J*_H-H_ = 7.8 Hz, 1H, H_m_ py), 7.28 (d, *J*_H-H_ = 1.9 Hz, 1H, =CH Im), 7.18 (d, *J*_H-H_ = 1.9 Hz, 1H, =CH Im), 7.14 (d, *J*_H-H_ = 7.8 Hz, 1H, H_m_ py), 5.40 (s, 2H, CH_2_Im), 4.51 (s, 2H, CH_2_OMe), 3.39 (s, 3H, OCH_3_), 1.70 (s, 9H, ^t^Bu). ^13^C{^1^H} NMR (298 K, 75 MHz, CD_3_CN): δ 177.1 (C_NCN_), 159.8, 156.4 (C_q_ py), 138.9 (C_p_ py), 121.8, 121.7 6 (C_m_ py), 121.4, 119.8 (=CH, Im), 75.8 (CH_2_OMe), 58.9 (OCH_3_), 58.6 (C ^t^Bu), 57.8 (CH_2_Im), 32.0 (CH_3_^t^Bu).

### [Cu_2_(μ-Br)_2_(^t^BuImCH_2_pyCH_2_NEt_2_)]_*n*_ (**5**)

[^t^BuHImCH_2_pyCH_2_NEt_2_]Br (**2**) (200 mg, 0.524 mmol), Ag_2_O (255 mg, 1.100 mmol), and Cu powder (200 mg, 3.144 mmol). Yield: 166 mg, 54% (pale green solid). Anal. calcd for C_18_H_28_Br_2_N_4_Cu_2_: 36.81 C, 4.81 H, 9.54 N. Found: 36.42 C, 5.15 H, 9.84 N. HRMS (ESI+, CH_3_CN, *m*/*z*): calcd for C_36_H_58_Br_2_Cu_4_N_8_ [2M + 2H-2Br]^+^: 1012.0335, found: 1012.0384; calcd for C_18_H_31_BrCuN_4_ [M + 2H-Br-Cu]^+^: 445.1028, found: 445.0860; calcd for C_18_H_28_CuN_4_ [M-2Br-Cu]^+^: 363.1610, found: 363.1619; calcd for C_18_H_28_N_4_ [M-2Br-2Cu + H]^+^: 301.2392, found: 301.2392. ^1^H NMR (298 K, 300 MHz, CD_3_CN): δ 7.75 (t, *J*_H-H_ = 7.7 Hz, 1H, H_p_ py), 7.40 (d, *J*_H-H_ = 7.7 Hz, 1H, H_m_ py), 7.26 (d, *J*_H-H_ = 1.8 Hz, 1H, =CH Im), 7.22–7.16 (m, 2H, H_m_ py, =CH Im), 5.42 (s, 2H, CH_2_Im), 3.75 (s, 2H, CH_2_NEt_2_), 2.59 (q, *J*_H-H_ = 7.1 Hz, 4H, CH_2_ Et), 1.70 (s, 9H, ^t^Bu), 1.01 (t, *J*_H-H_ = 7.1 Hz, 6H, CH_3_ Et). ^13^C{^1^H} NMR (298 K, 75 MHz, CD_3_CN): δ 178.1 (C_NCN_), 160.5, 156.3 (C_q_ py), 139.3 (C_p_ py), 123.4 (C_m_ py), 122.3 (=CH Im), 121.5 (C_m_ py), 119.6 (=CH Im), 60.2 (CH_2_NEt_2_), 58.5 (C ^t^Bu), 57.7 (CH_2_Im), 48.3 (CH_2_ Et), 31.8 (CH_3_^t^Bu), 11.7 (CH_3_ Et).

### [Cu_2_(μ-Br)_2_(^t^BuImCH_2_pyCH_2_NH^t^Bu)]_*n*_ (**6**)

[^t^BuHImCH_2_pyCH_2_NH^t^Bu]Br (**3**) (200 mg, 0.524 mmol), Ag_2_O (255 mg, 1.100 mmol), and Cu powder (200 mg, 3.144 mmol). Yield: 160 mg, 52% (pale green solid). Anal. calcd for C_18_H_28_Br_2_N_4_Cu_2_: 36.81 C, 4.81 H, 9.54 N. Found: 36.56 C, 4.85 H, 9.48 N. HRMS (ESI+, CH_3_CN, *m*/*z*): calcd for C_18_H_29_BrCu_2_N_4_ [M-Br + H]^+^: 506.0168, found: 506.5417; calcd for C_18_H_28_N_4_ [M-2Br-2Cu + H]^+^: 301.2392, found: 301.2691. ^1^H NMR (298 K, 300 MHz, CD_3_CN): δ 7.80 (t, *J*_H-H_ = 7.8 Hz, 1H, H_p_ py), 7.38–7.28 (m, 3H, H_m_ py, 2 =CH Im), 7.17 (d, *J*_H-H_ = 7.7 Hz, 1H, H_m_ py), 5.76 (s, 2H, CH_2_Im), 4.07 (m, 2H, C*H_2_*NH^t^Bu), 3.35 (t, *J*_H-H_ = 7.2 Hz, 1H, NH), 1.69 (s, 9H, ^t^BuIm), 1.27 (s, 9H, ^t^BuNH). ^13^C{^1^H} NMR (298 K, 75 MHz, CD_3_CN): δ 177.9 (C_NCN_), 160.7, 156.4 (C_q_ py), 140.1 (C_p_ py), 123.3 (C_m_ py), 121.7, 119.6 (=CH Im), 58.7 (C ^t^BuIm), 57.7 (CH_2_Im), 54.3 (C ^t^BuNH), 48.7 (CH_2_NH^t^Bu), 31.9 (CH_3_^t^BuIm), 29.1 (CH_3_^t^BuNH).

### [(CuBr)_2_(C≡CPh)(^t^BuHImCH_2_pyCH_2_NEt_2_)] (**10**)

A moderate excess of phenylacetylene (0.125 mmol) was added to a solution of **5** (15 mg, 0.025 mmol) in CD_3_CN (0.5 mL) at 243 K to give immediately a yellow-brown solution. The acetylide compound formed was characterized spectroscopically in the solution. ^1^H NMR (298 K, 300 MHz, CD_3_CN): δ 9.19 (s, ^1^H, NCHN), 8.18 and 7.26 (d, 1H, *J*_H-H_ = 7.7, H_m_ py), 7.84 (t, *J*_H-H_ = 7.7, 1H, H_p_ py), 7.54 and 7.47 (br, 1H, =CH Im), 7.5–7.0 (m, 5H, Ph), 5.53 (s, 2H, CH_2_Im), 3.69 (s, 2H, CH_2_NEt_2_), 2.55 (q, *J*_H-H_ = 7.0, 4H, CH_2_ Et), 1.68 (s, 9H, ^t^Bu), 1.02 (t, *J*_H-H_ = 7.0, 6H, CH_3_ Et). ^13^C{^1^H}-APT NMR (243 K, 100.0 MHz, CD_3_CN): δ 161.70 and 153.4 (C_q_ py), 139.2 (NCHN), 132.5 (C_p_ py), 131.6, 130.4 and 129.1 (CH Ph), 129.6 and 124.3 (both C_m_ py), 128.1 (C_q_ Ph), 126.7 (Cu–*C*≡C), 124.1 and 121.0 (=CH Im), 98.8 (Cu–C≡*C*), 61.2 (C ^t^Bu), 59.9 (CH_2_NEt_2_), 54.4 (s, CH_2_Im), 47.7 (CH_2_ Et), 29.9 (CH_3_^t^Bu). 12.0 (CH_3_ Et).

### General Procedure for the Copper-Catalyzed Azide–Alkyne Cycloaddition Reactions

The catalytic reactions were carried out under an argon atmosphere under solvent-free conditions. First, the catalyst (0.005 mmol) was weighted in a Schlenk tube in a glovebox. Alternatively, in the experiments with a low catalyst load, a stock 8.5 × 10^–4^ M solution of the catalyst in acetonitrile was prepared in the glovebox from which the required volume was taken using a precision microsyringe, transferred to a Schlenk tube, and then brought to dryness under a vacuum. At this point, azide (0.5 mmol), alkyne (0.5 mmol), and mesitylene (0.25 mmol) as internal standard were sequentially added, and the Schlenk tube was introduced in a thermostatic bath at the desired temperature. *Caution*: CuAAC is a highly exothermic reaction, and scale-up of the reaction without a solvent can have a significant effect on the likelihood of runaway.^41^

Conversions and selectivities were determined by gas chromatography analysis of aliquots of the reaction mixture or by dissolving the solid formed in dichloromethane under the following conditions: column temperature of 80 °C (4 min) to 250 °C at a heating rate of 20 °C min^–1^ by using ultrapure He as carrier gas, and temperatures of 250 °C for the injector and the FID detector.

The 1,4-substituted 1,2,3-triazol reaction products were isolated in yields close to or greater than 90%. Typically, the reaction mixture was brought to dryness under a vacuum to give a white residue that was washed with pentane (3 × 10 mL) and dried under a vacuum.^10b^

### Crystal Structure Determination

Single crystals of **5** suitable for the X-ray diffraction studies were grown by slow diffusion of *n*-hexane into a dichloromethane solution of the compound. X-ray diffraction data were collected at 100(2) K on a Bruker APEX SMART CCD diffractometer with graphite-monochromated Mo–Kα radiation (λ = 0.71073 Å) using <1° ω rotations. Intensities were integrated and corrected for absorption effects with SAINT-PLUS^42^ and SADABS^43^ programs, both included in the APEX2 package. The structures were solved by the Patterson method with SHELXS-97^44^ and refined by full matrix least squares on *F*^2^ with SHELXL-2014^45^ under WinGX.^46^

### Crystal Data and Structure Refinement for **5**

C_18_H_28_Br_2_Cu_2_N_4_, 587.34 g·mol^–1^, monoclinic, *C*2/*c*, *a* = 28.8644(18) Å, *b* = 10.0279(6) Å, *c* = 15.8988(10) Å, β = 108.8500(10)°, *V* = 4355.1(5) Å^3^, *Z* = 8, *D*_calc_ = 1.792 g·cm^3^, μ = 5.633 mm^–1^, F(000) = 2336, 0.280 × 0.100 × 0.060 mm^3^, θ_min_/θ_max_ 2.163/26.372°, −36 ≤ *h* ≤ 36, −12 ≤ *k* ≤ 12, −19 ≤ *l* ≤ 19, reflections collected/independent 33,953/4445 [*R*(int) = 0.0349], *T*_max_/*T*_min_ 0.4920/0.3044, data/restraints/parameters 4445/0/240, GooF(*F*^2^) = 1.032, *R*_1_ = 0.0321 [*I* > 2·σ(*I*)], *wR*_2_ = 0.0892 (all data), largest diff. peak/hole 1.350/–1.129 e·Å^–3^, CCDC deposition number 2091444.

### Computational Details

DFT calculations were performed by means of the Gaussian 09 software package, revision D01.^47^ We selected the B3LYP exchange-correlation functional^48^ in conjunction with the D3BJ empirical dispersion correction scheme,^49^ which has been recently applied to similar processes.^23e^ For geometry optimizations and transition state search, we applied the Ahlrichs def2-SVP basis set, while energy results were further refined via single point calculations with the triple-zeta def2-TZVP basis set.^50^ Solvent effects were modeled through the polarizable continuum model (PCM) approach, as implemented in the Gaussian 09 suite, and were considered in both gradients and energy calculations.^51^ We selected acetonitrile as the solvent as many experiments were performed in such solvent and it is much easier to model than neat conditions, which would involve a mixture of the azide and the alkyne. Notice that we applied an ″ultrafine″ grid in all the calculations. The nature of the stationary points has been confirmed by analytical frequency analysis, which was also applied for the calculation of Gibbs energy corrections (at 298.15 K and considering a reference concentration of 1 M). The CylView software was used for structure graphical representations.^52^

## Supporting Information Available

NMR spectra of functionalized imidazolium salts and copper(I) compounds; isolation procedure, characterization, and NMR spectra of 1,4-substituted-1,2,3-triazole derivatives, and DFT energy data (PDF)
Optimized coordinates for catalytic intermediates and transition states (XYZ)

## Supplementary Material

om2c00246_si_001.pdf

om2c00246_si_002.xyz
