# Supplementary material for: Copper-Catalyzed Azide–Alkyne Cycloaddition (CuAAC) by Functionalized NHC-Based Polynuclear Catalysts: Scope and Mechanistic Insights
Source: Organometallics. 2022 Jul 15;41(15):2154–69. doi: 10.1021/acs.organomet.2c00246 (PMC9374069; doi:10.1021/acs.organomet.2c00246)
Supplement: Supplementary file 1 — om2c00246_si_001.pdf [file om2c00246_si_001.pdf]

Supporting Information

Copper-Catalyzed Azide–Alkyne Cycloaddition (CuAAC) by Functionalized NHC-based Polynuclear Catalysts: Scope and Mechanistic Insights

Miguel González-Lainez,[a] Miguel Gallegos,[b] Julen Munarriz,[b] Ramón Azpiroz,[a] Vincenzo Passarelli,[a] M. Victoria Jiménez,[,a] and Jesús J. Pérez-Torrente[*,a]*

[a] Departamento de Química Inorgánica, Instituto de Síntesis Química y Catálisis Homogénea-ISQCH, Universidad de Zaragoza-C.S.I.C., 50009-Zaragoza, Spain.

[b] Departamento de Química Física y Analítica. Universidad de Oviedo, 33006-Oviedo, Spain.

Emails: M. V. Jiménez: vjimenez@unizar.es, J. J. Pérez-Torrente: perez@unizar.es

Contents

1.- NMR spectra of functionalized imidazolium salts **2**-**3**. S2

2.- NMR and HRMS spectra of copper(I) compounds **4**-**6**. S5

3.- Formation of **10**. S17

4.- Isolation and characterization of 1,4-substituted-1,2,3-triazole compounds. S21

5.- NMR spectra of 1,4-substituted-1,2,3-triazole compounds. S25

6.- Deuterium labeling studies. S38

7.- DFT energy data. S42

8.- References. S43

1.- NMR spectra of functionalized imidazolium salts 2-3.

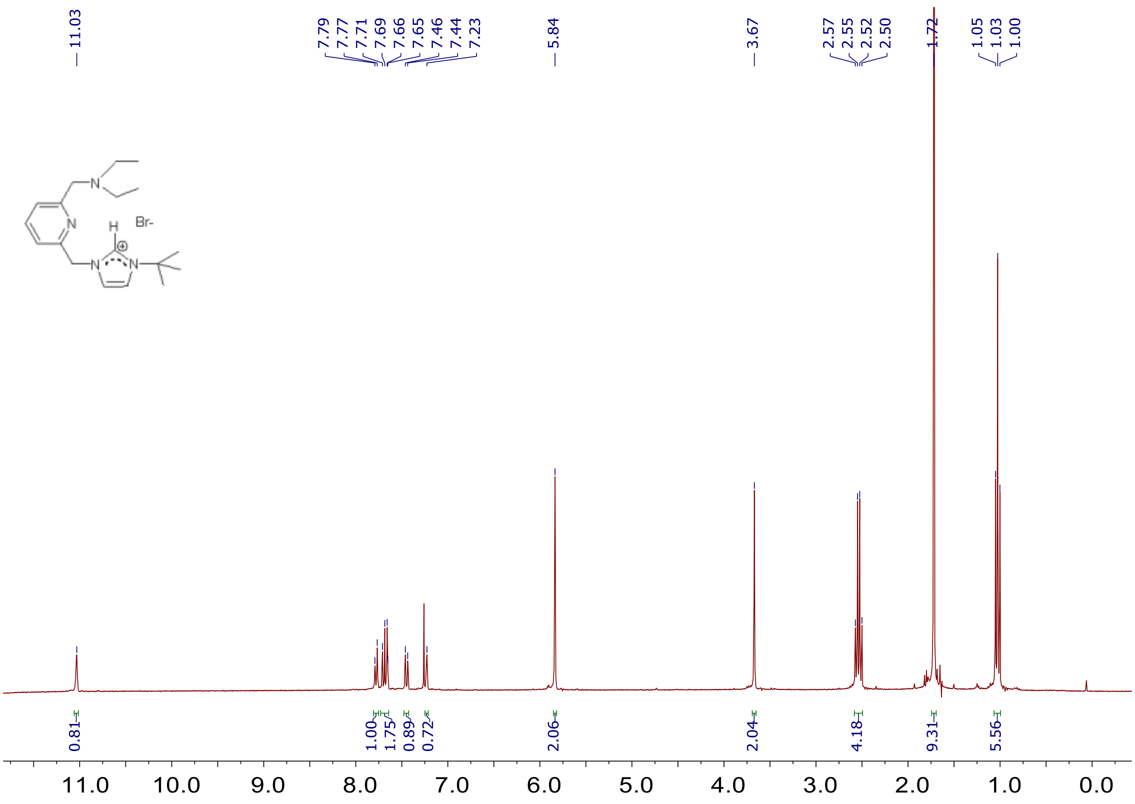

Figure S1. ^{1}H NMR ($CDCl_3$) of (^{t}BuHImCH$_2$PyCH$_2$NMe$_2$)Br (**2**).

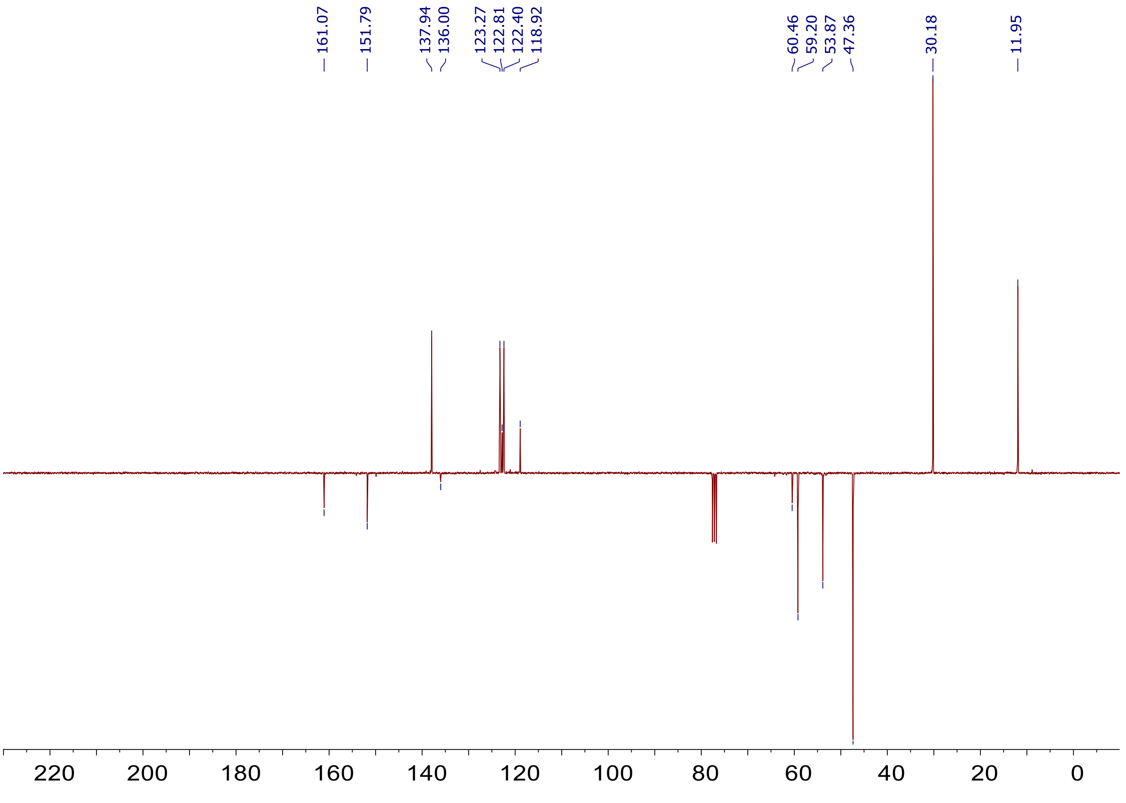

Figure S2. $^{13}C\{^1H\}$ NMR ($CDCl_3$) of (^{t}BuHImCH$_2$PyCH$_2$NMe$_2$)Br (**2**).

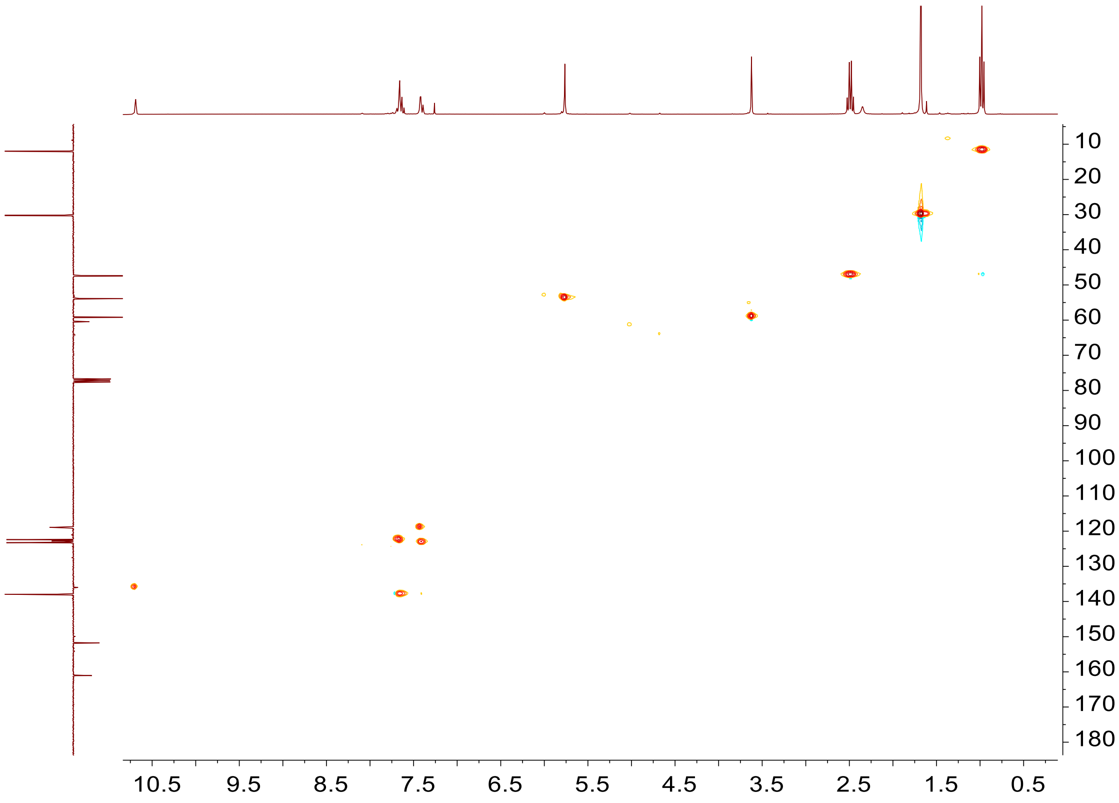

Figure S3. ^{1}H/^{13}C-hsqc NMR ($CDCl_3$) of (^{t}BuHImCH$_2$PyCH$_2$NMe$_2$)Br (**2**).

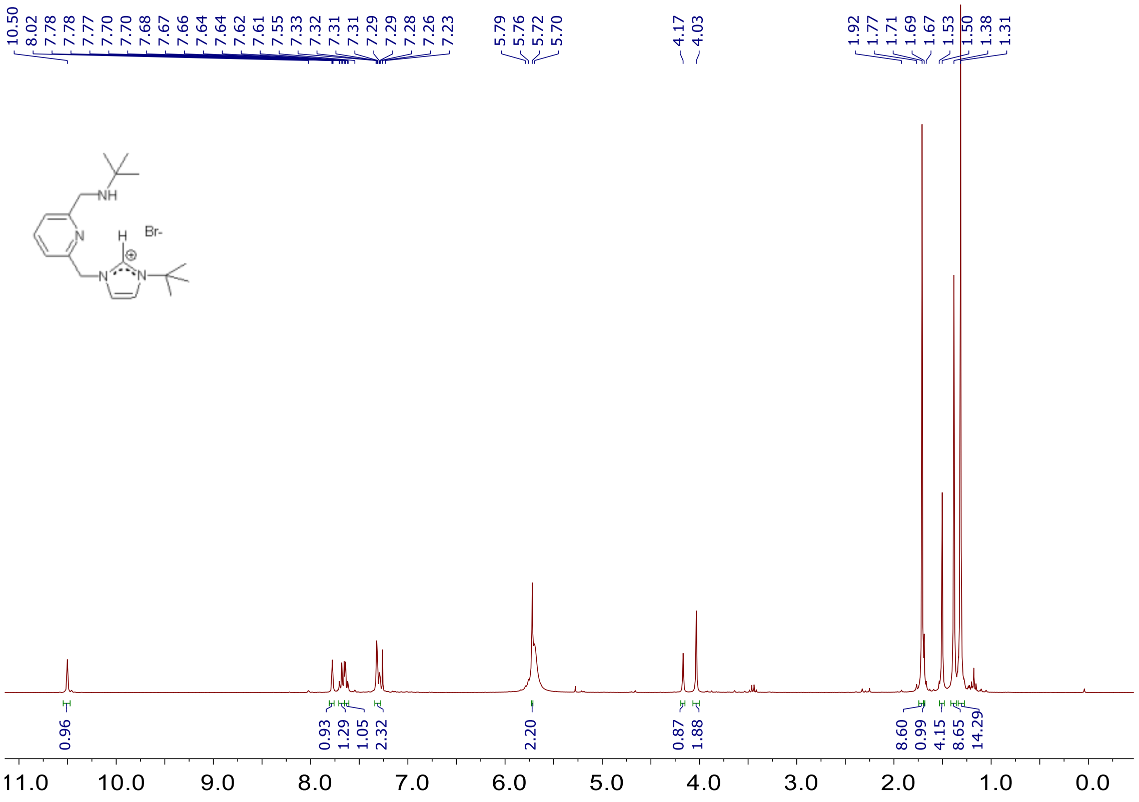

Figure S4. ^{1}H NMR ($CDCl_3$) of (^{t}BuHImCH$_2$PyCH$_2$NH^{t}Bu)Br (**3**).

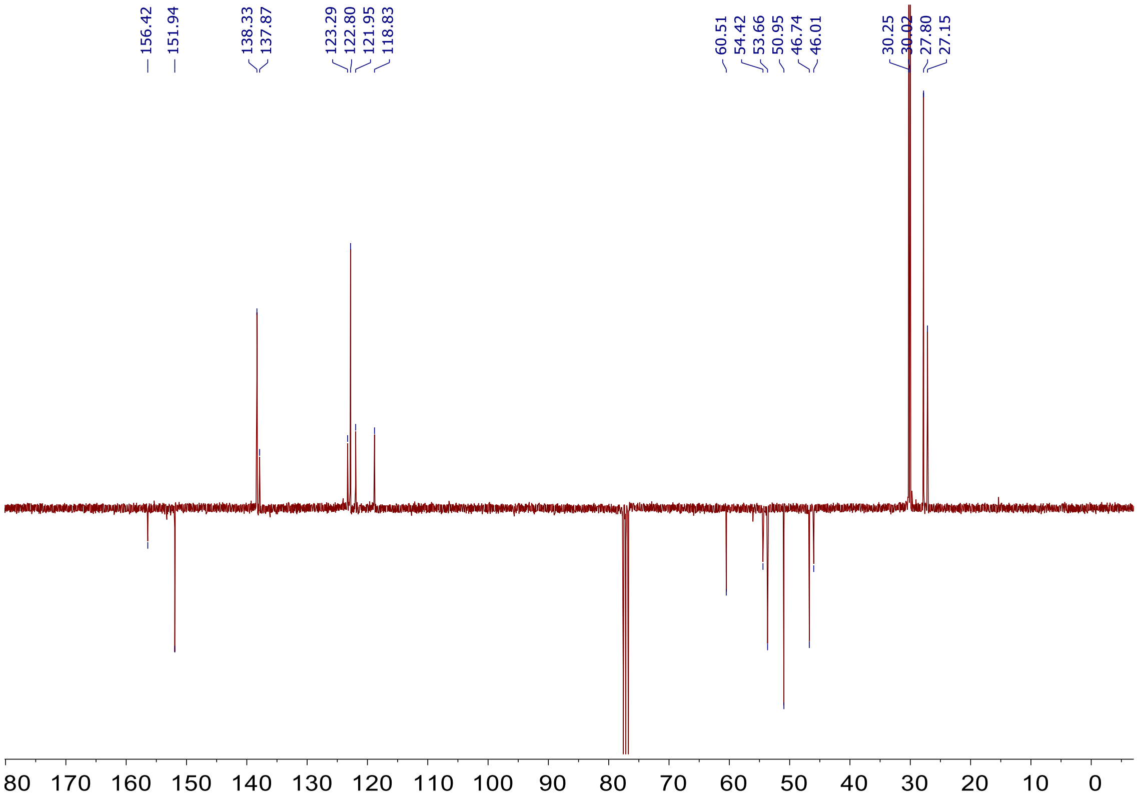

Figure S5. $^{13}C\{^{1}H\}$ NMR ($CDCl_3$) of (^{t}BuHImCH$_2$PyCH$_2$NH^{t}Bu)Br (**3**).

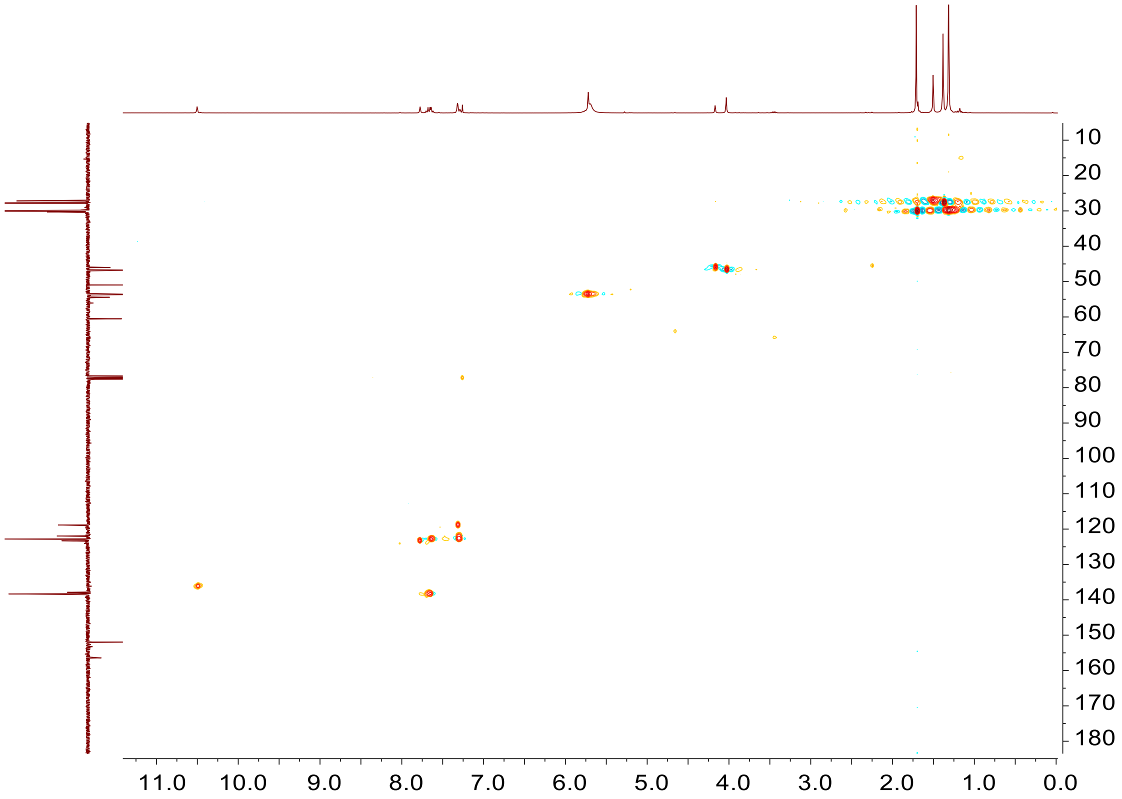

Figure S6. $^{1}H/^{13}C$-hsqc NMR ($CDCl_3$) of (^{t}BuHImCH$_2$PyCH$_2$NH^{t}Bu)Br (**3**).

2.- NMR spectra of copper(I) compounds 4-6.

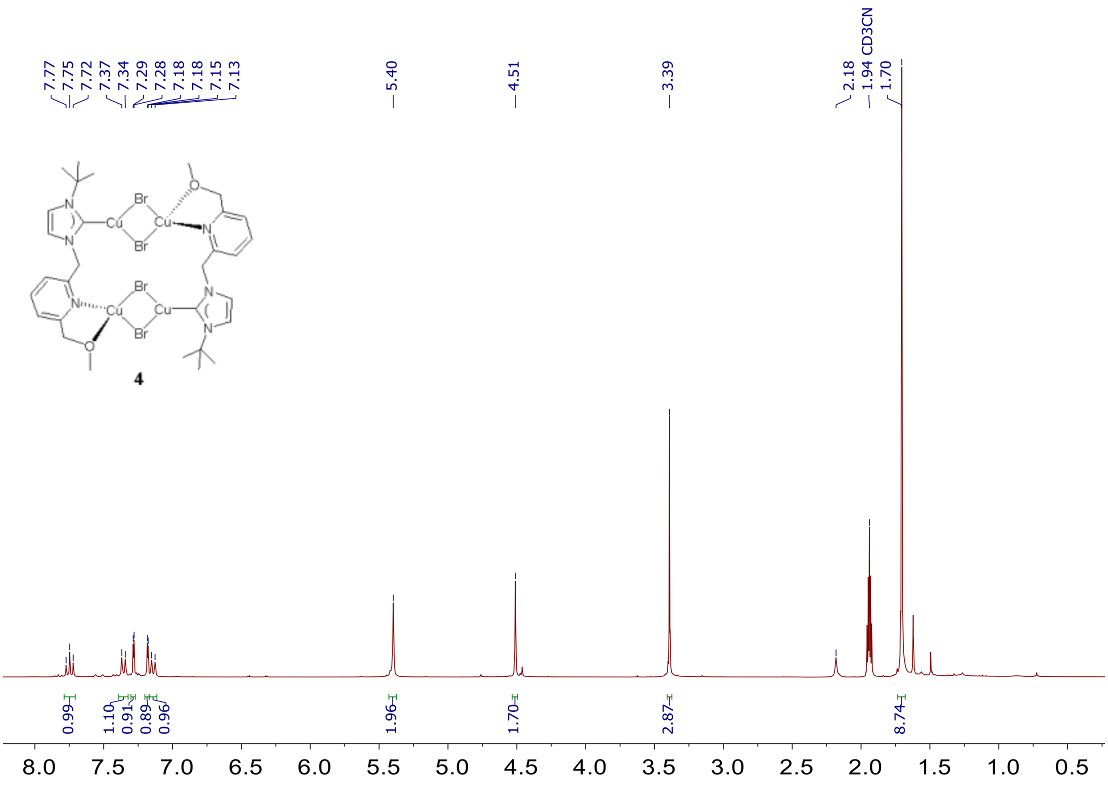

Figure S7. ^{1}H NMR (CD_3CN) of $[Cu_2(\mu\text{-}Br)_2(^{t}BuImCH_2PyCH_2OMe)]_n$ (**4**).

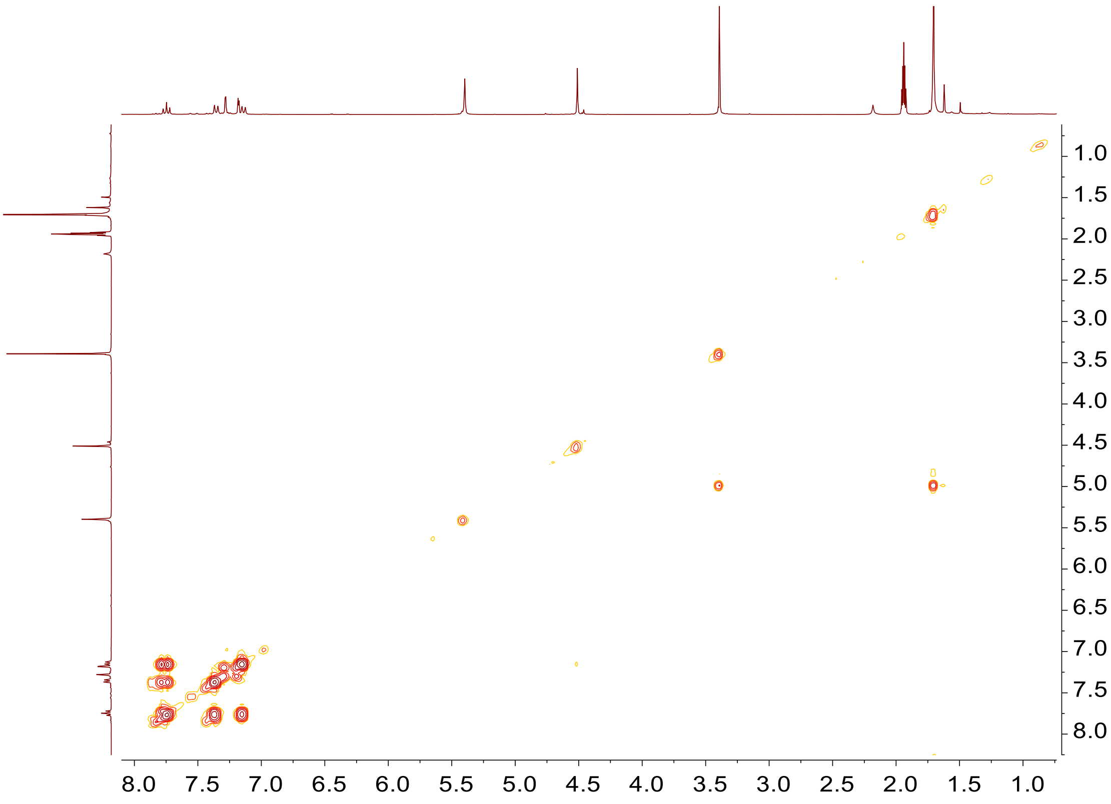

Figure S8. $^{1}H/^{1}H$-cosy NMR (CD_3CN) of $[Cu_2(\mu\text{-}Br)_2(^{t}BuImCH_2PyCH_2OMe)]_n$ (**4**).

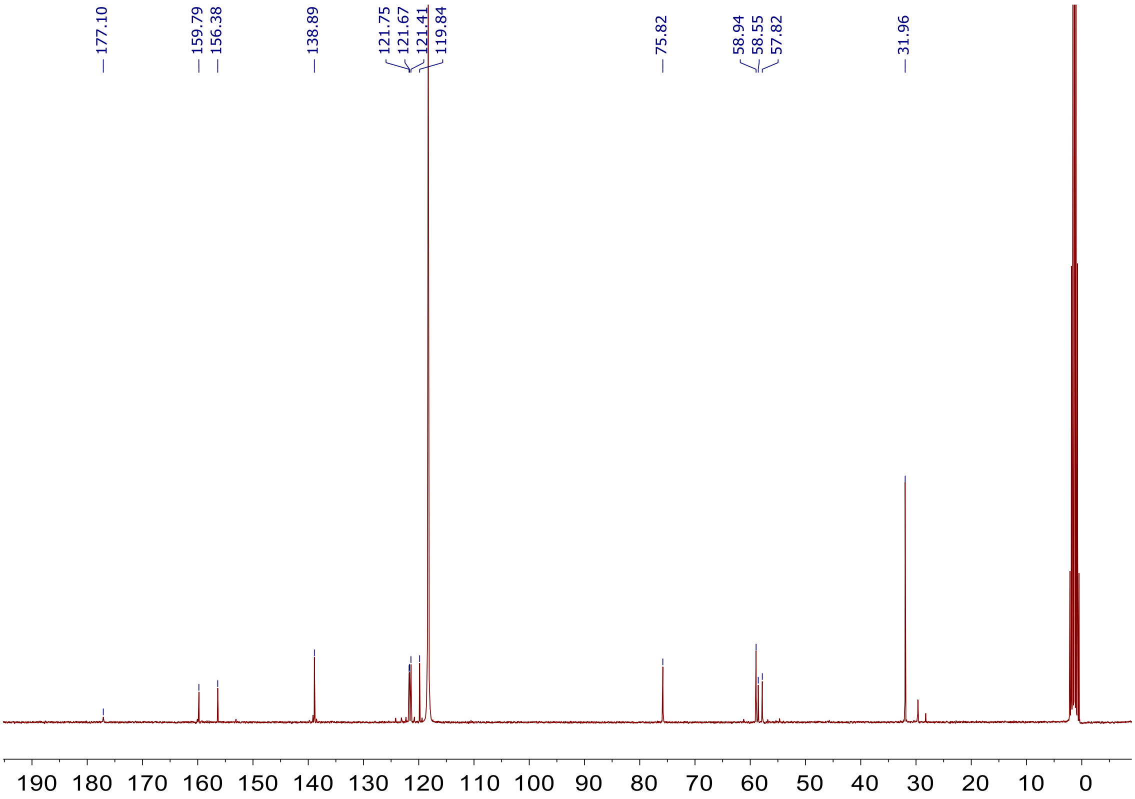

Figure S9. $^{13}C\{^1H\}$ NMR (CD_3CN) of [Cu_2(μ-Br)$_2$(^{t}BuImCH$_2$PyCH$_2$OMe)]$_n$ (**4**).

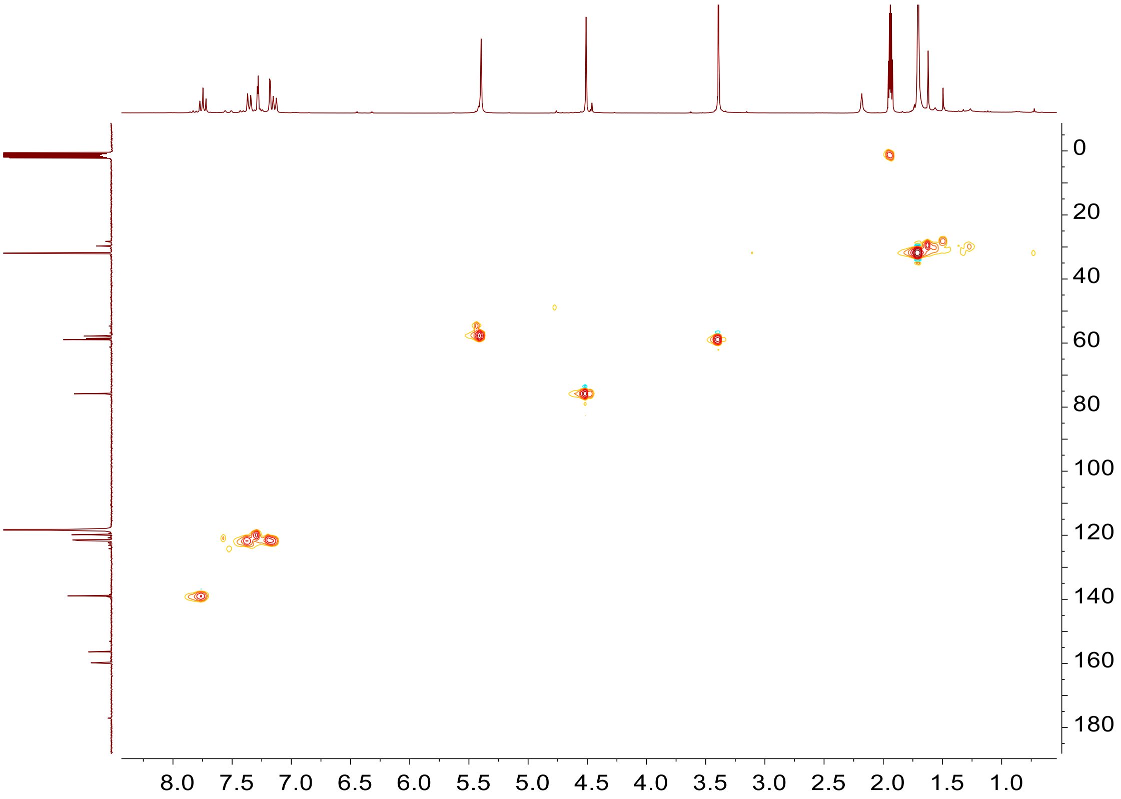

Figure S10. $^1H/^{13}C$-hsqc NMR (CD_3CN) of [Cu_2(μ-Br)$_2$(^{t}BuImCH$_2$PyCH$_2$OMe)]$_n$ (**4**).

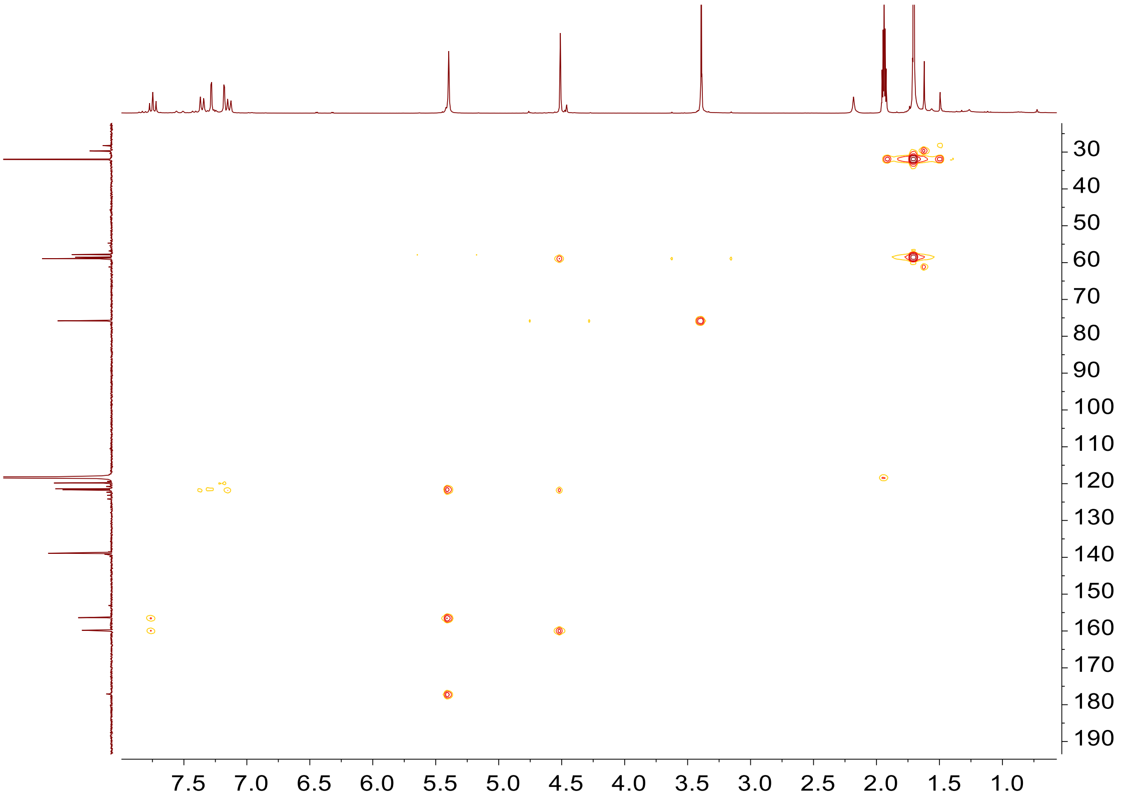

Figure S11. $^{1}H/^{13}C$-hmbc NMR (CD_3CN) of $[Cu_2(\mu\text{-}Br)_2(^{t}BuImCH_2PyCH_2OMe)]_n$ (**4**).

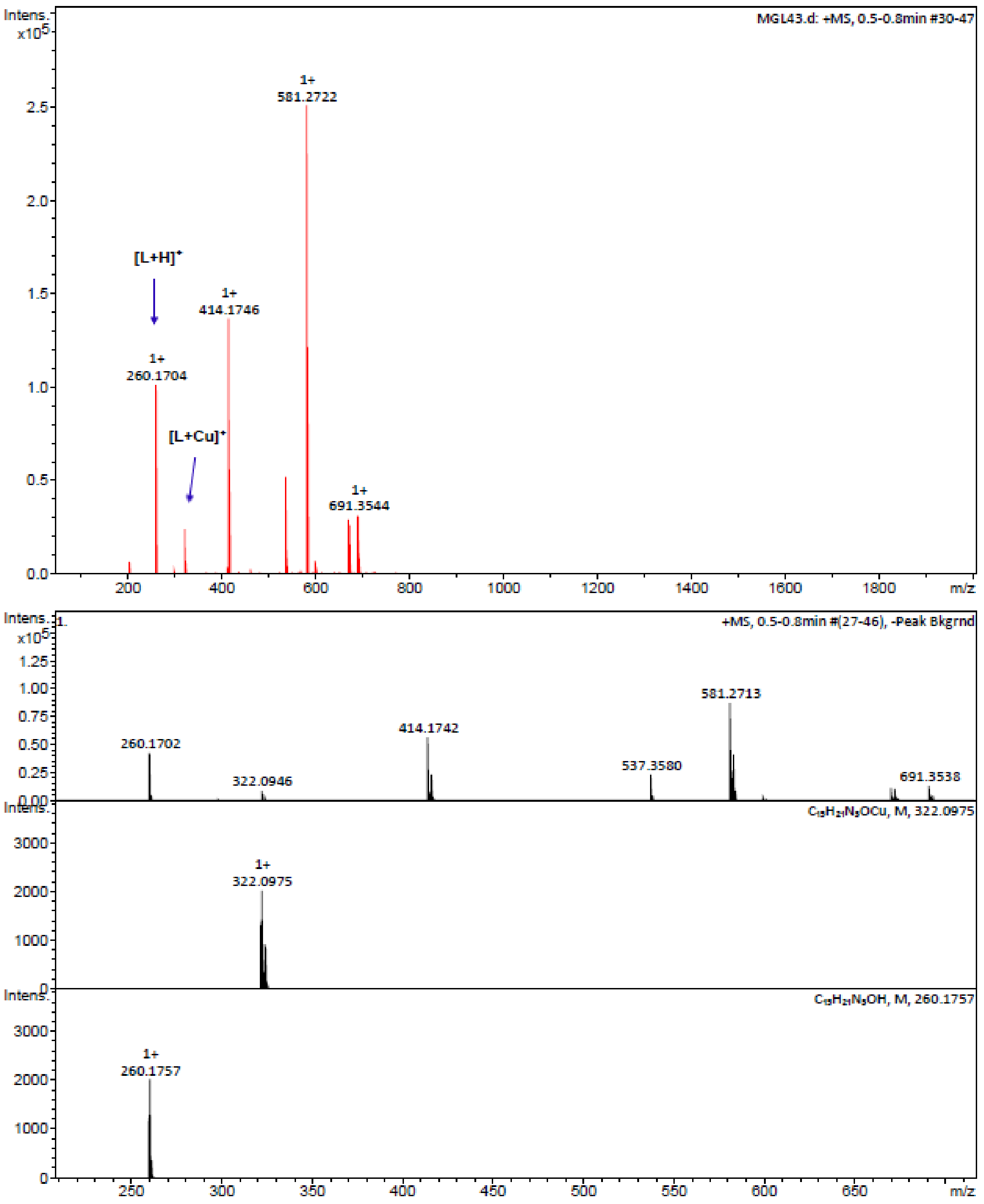

Figure S12. HRMS (ESI+, CH_3CN, m/z) of $[Cu_2(\mu\text{-}Br)_2(^tBuImCH_2PyCH_2OMe)]_n$ (**4**): 322.0946 $[L+Cu]^+$, 260.1704 $[L+H]^+$.

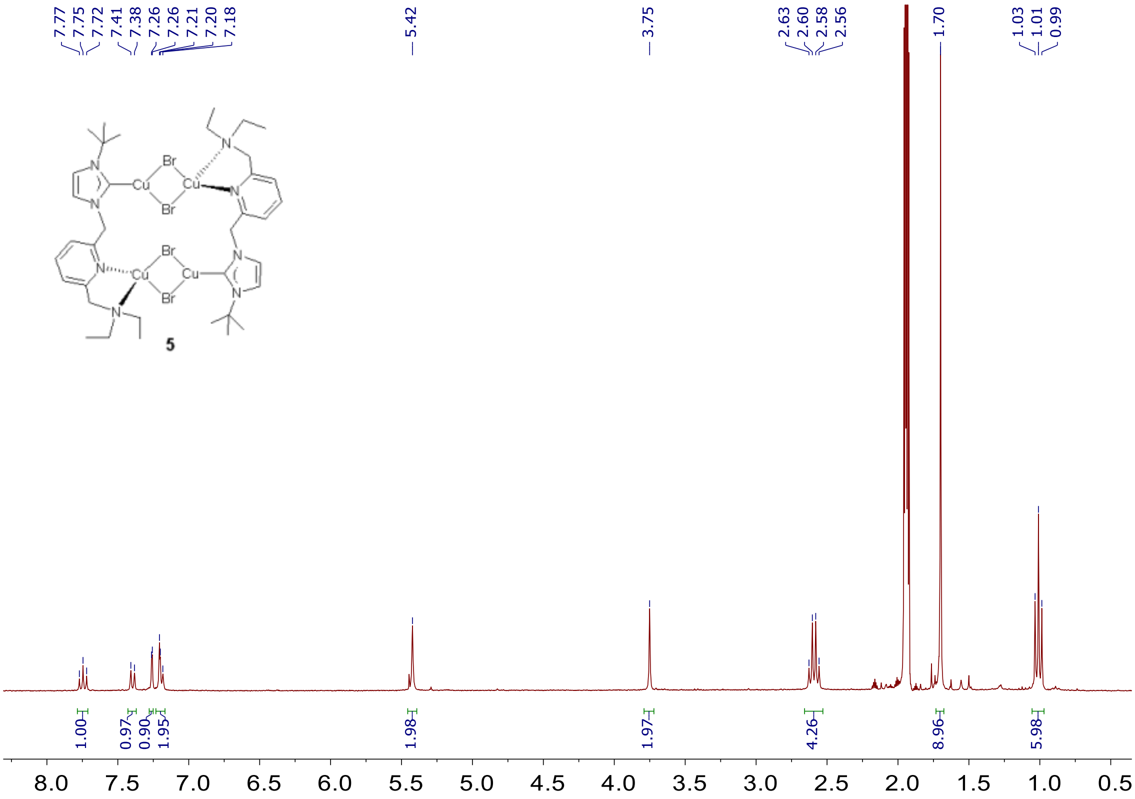

Figure S13. ^{1}H NMR (CD_3CN) of [Cu_2(μ-Br)$_2$(^{t}BuImCH$_2$PyCH$_2$NMe$_2$)]$_n$ (**5**).

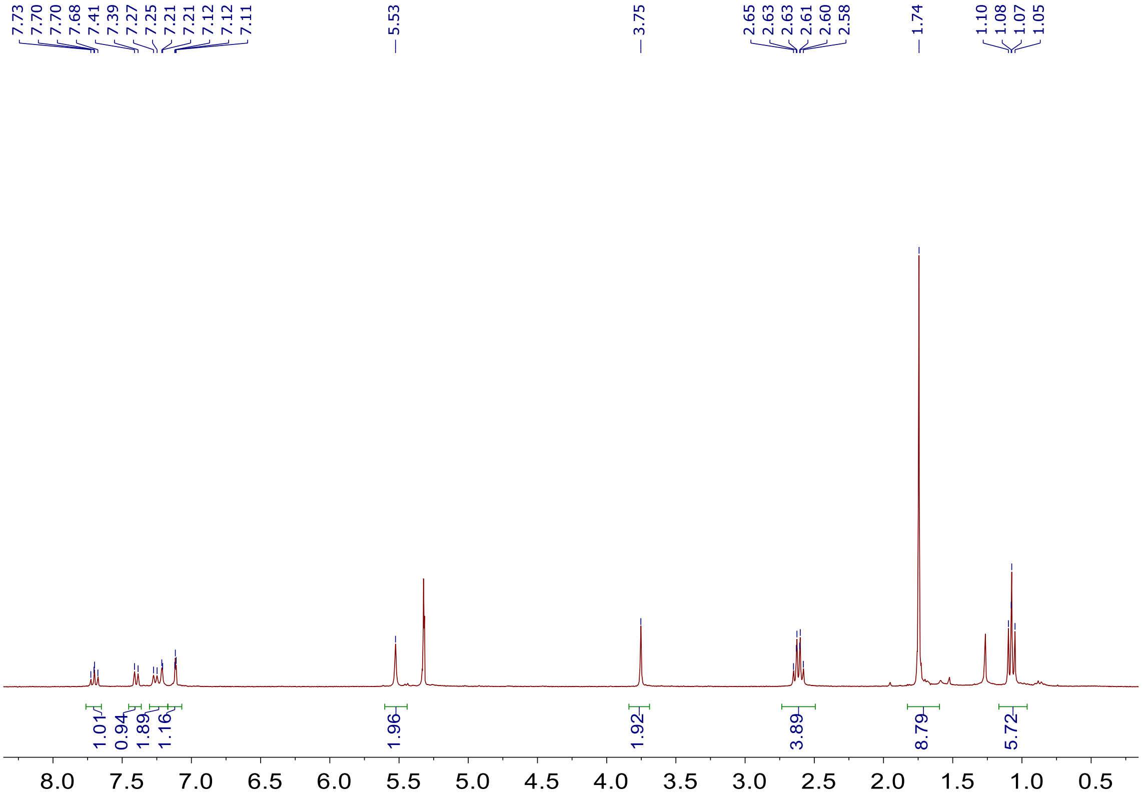

Figure S14. ^{1}H NMR (CD_2Cl_2) of [Cu_2(μ-Br)$_2$(^{t}BuImCH$_2$PyCH$_2$NMe$_2$)]$_n$ (**5**).

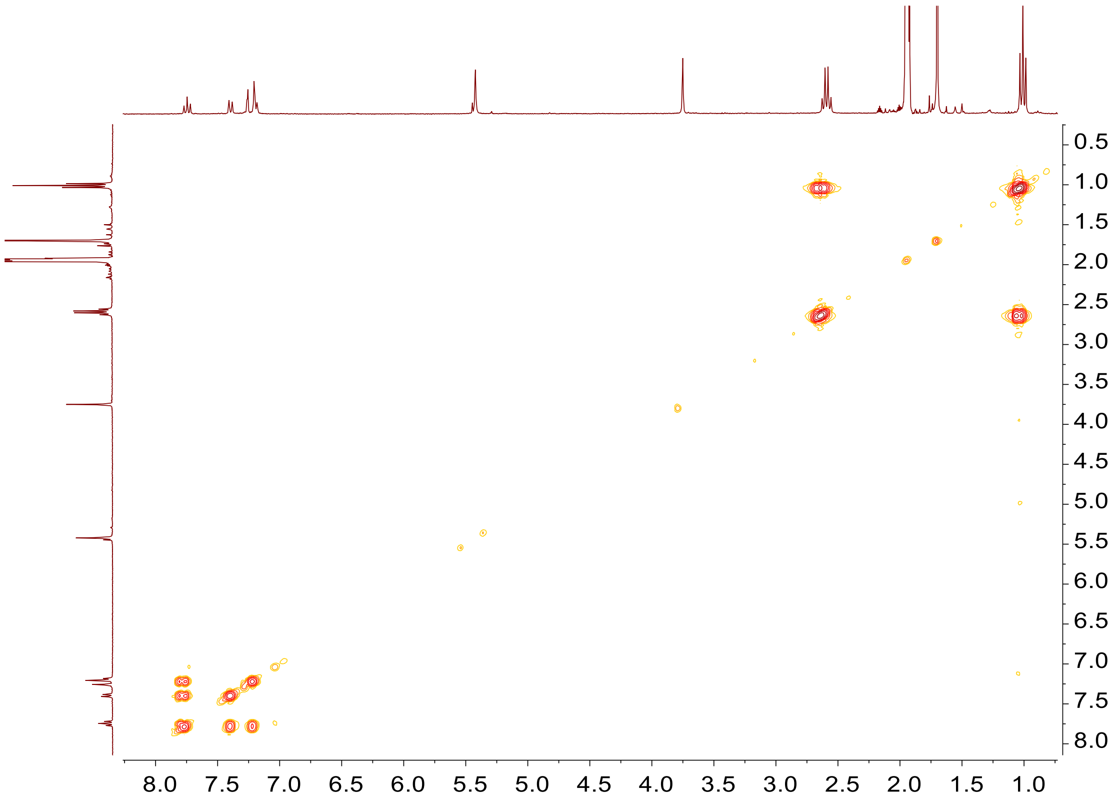

Figure S15. $^{1}H/^{1}H$-cosy NMR (CD_3CN) of $[Cu_2(\mu\text{-}Br)_2(^{t}BuImCH_2PyCH_2NMe_2)]_n$ (**5**).

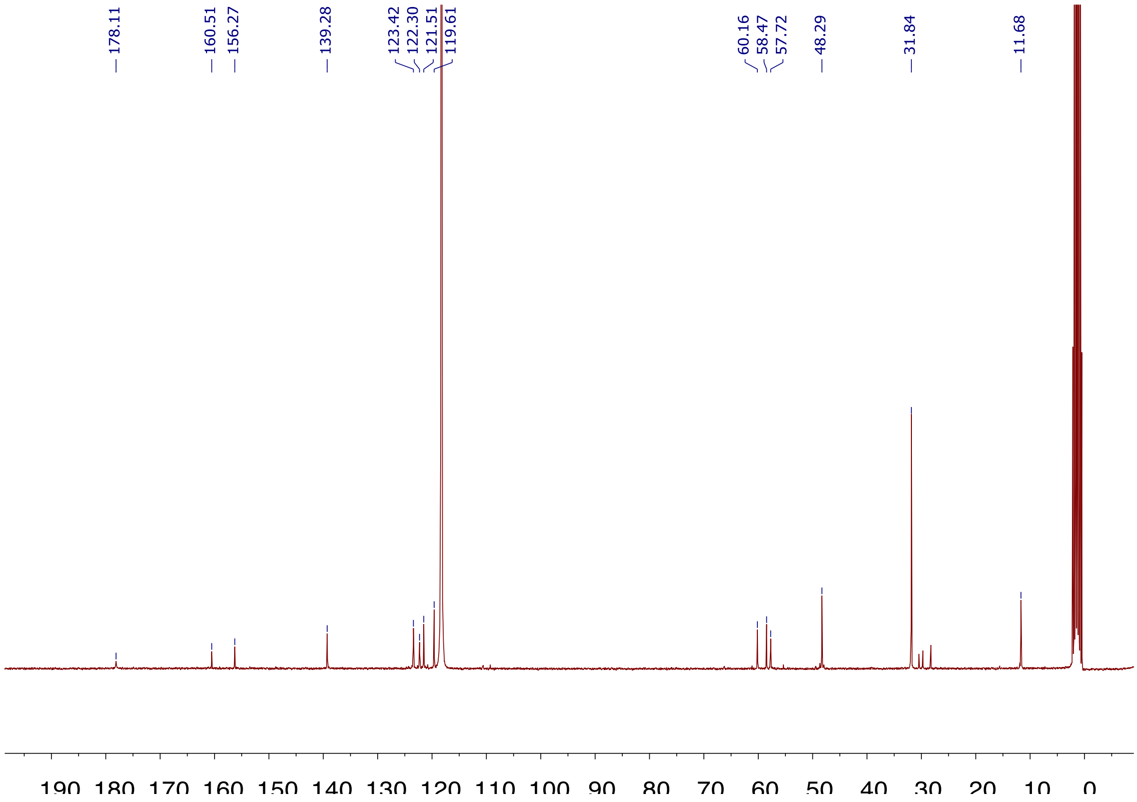

Figure S16. $^{13}C\{^{1}H\}$ NMR (CD_3CN) of $[Cu_2(\mu\text{-}Br)_2(^{t}BuImCH_2PyCH_2NMe_2)]_n$ (**5**).

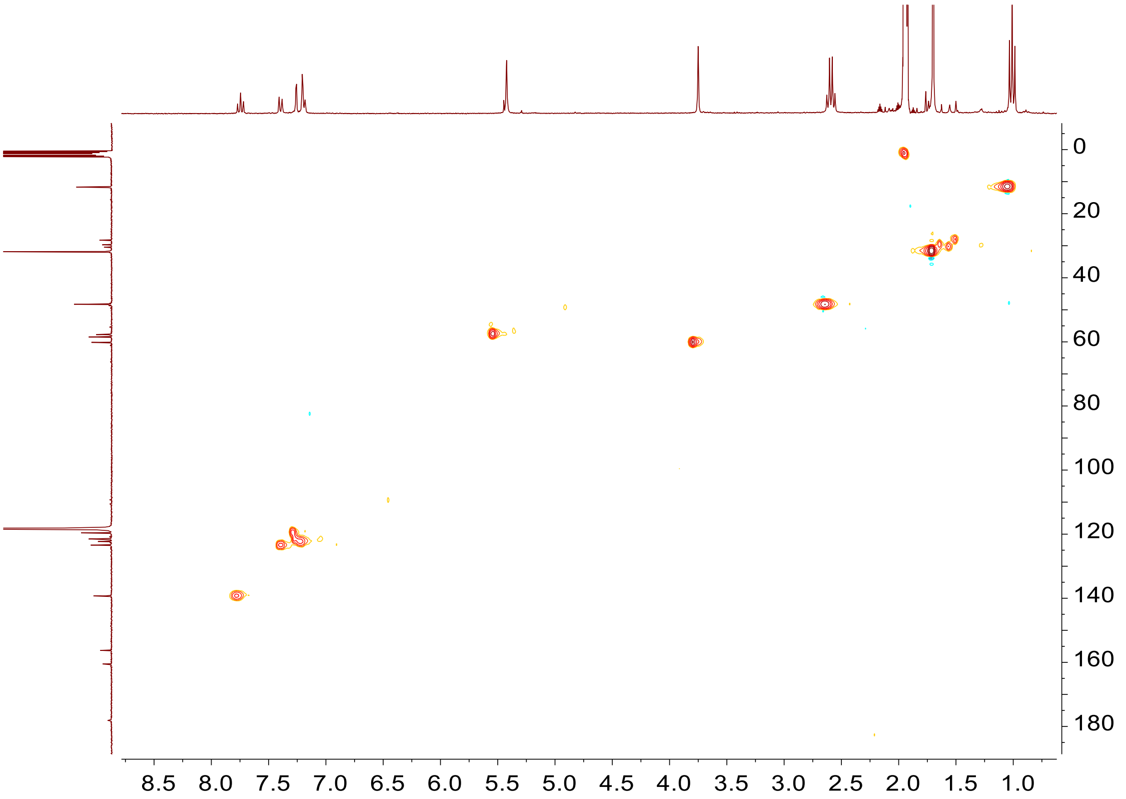

Figure S17. $^{1}H/^{13}C$-hsqc NMR (CD_3CN) of $[Cu_2(\mu\text{-}Br)_2(^{t}BuImCH_2PyCH_2NMe_2)]_n$ (**5**).

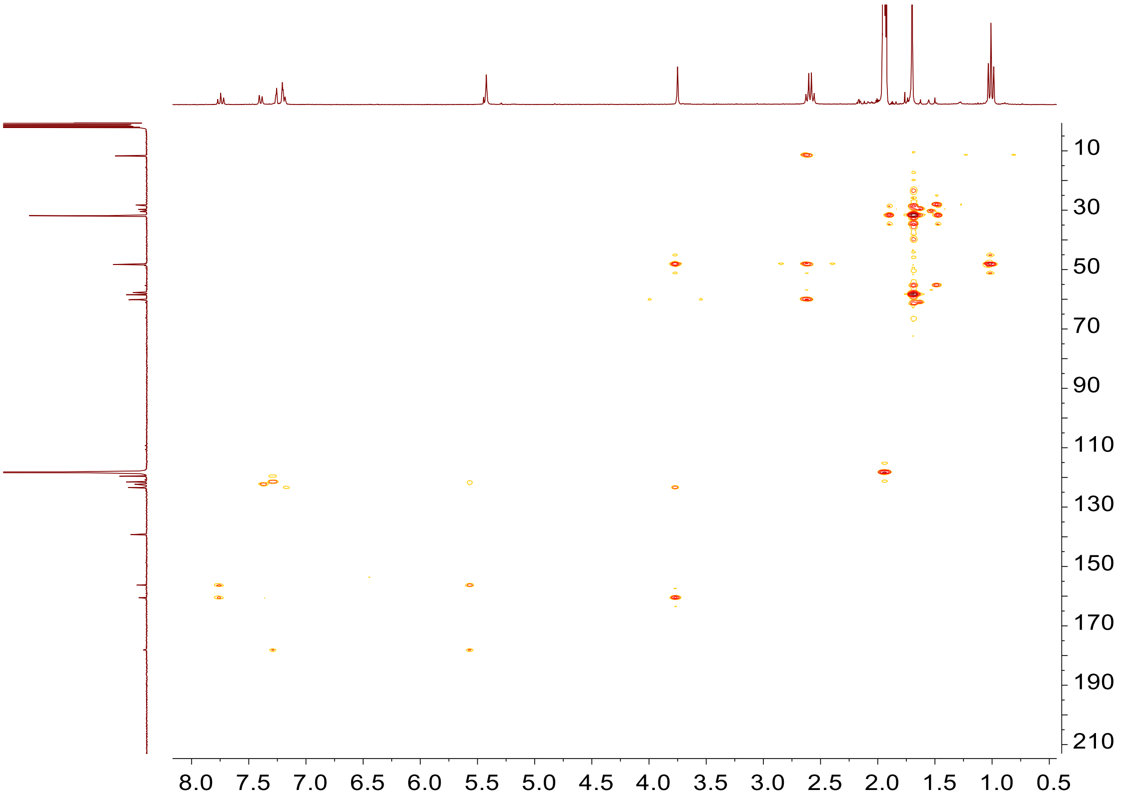

Figure S18. $^{1}H/^{13}C$-hmbc NMR (CD_3CN) of $[Cu_2(\mu\text{-}Br)_2(^{t}BuImCH_2PyCH_2NMe_2)]_n$ (**5**).

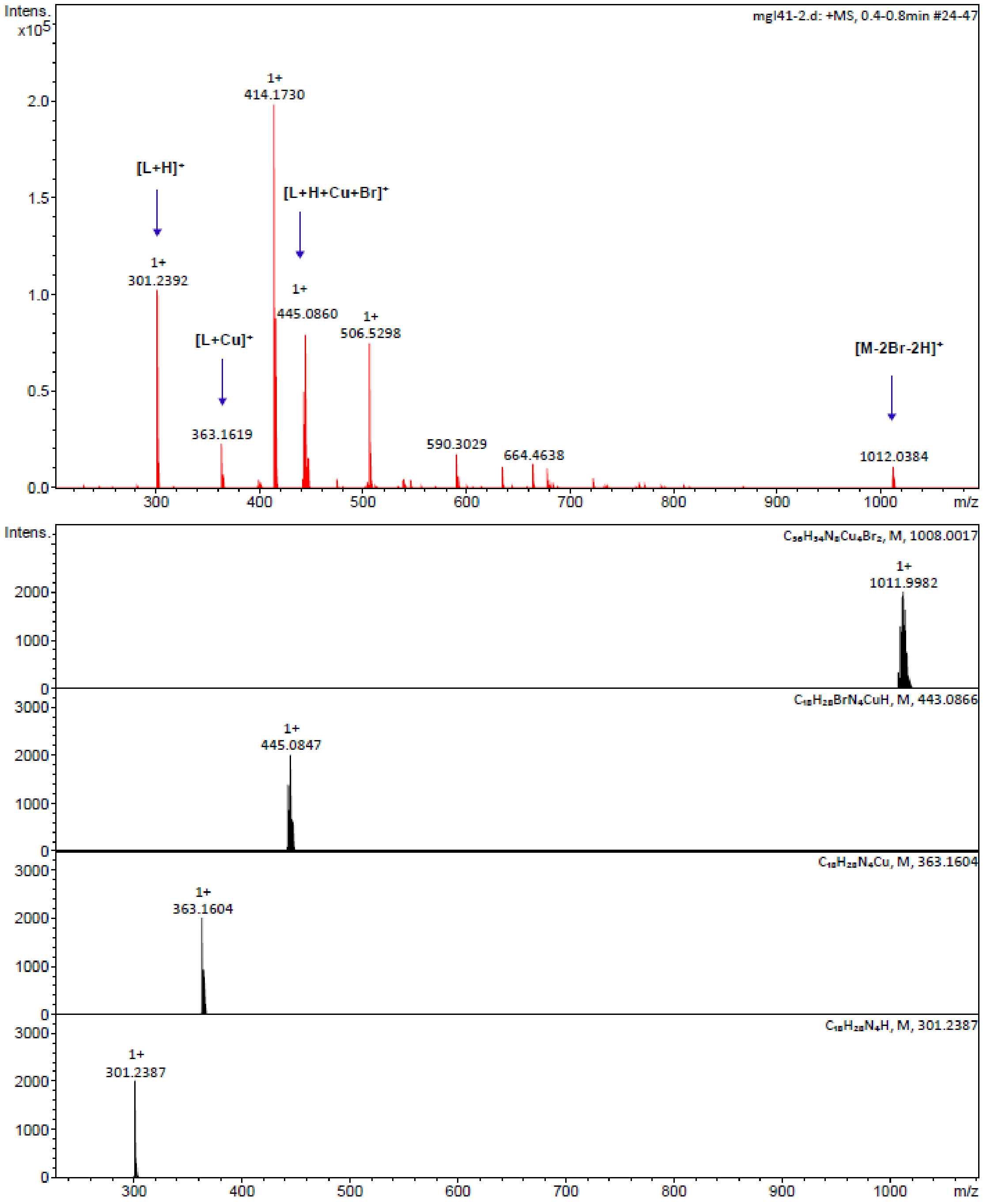

Figure S19. HRMS (ESI+, CH_3CN, m/z) of $[Cu_2(\mu\text{-}Br)_2(^{t}BuImCH_2PyCH_2NMe_2)]_n$ (**5**): 1012.0384 $[M\text{-}2Br\text{-}2H]^+$, 445.0860 $[L+H+Cu+Br]^+$, 363.1619 $[L+Cu]^+$, 301.2392 $[L+H]^+$.

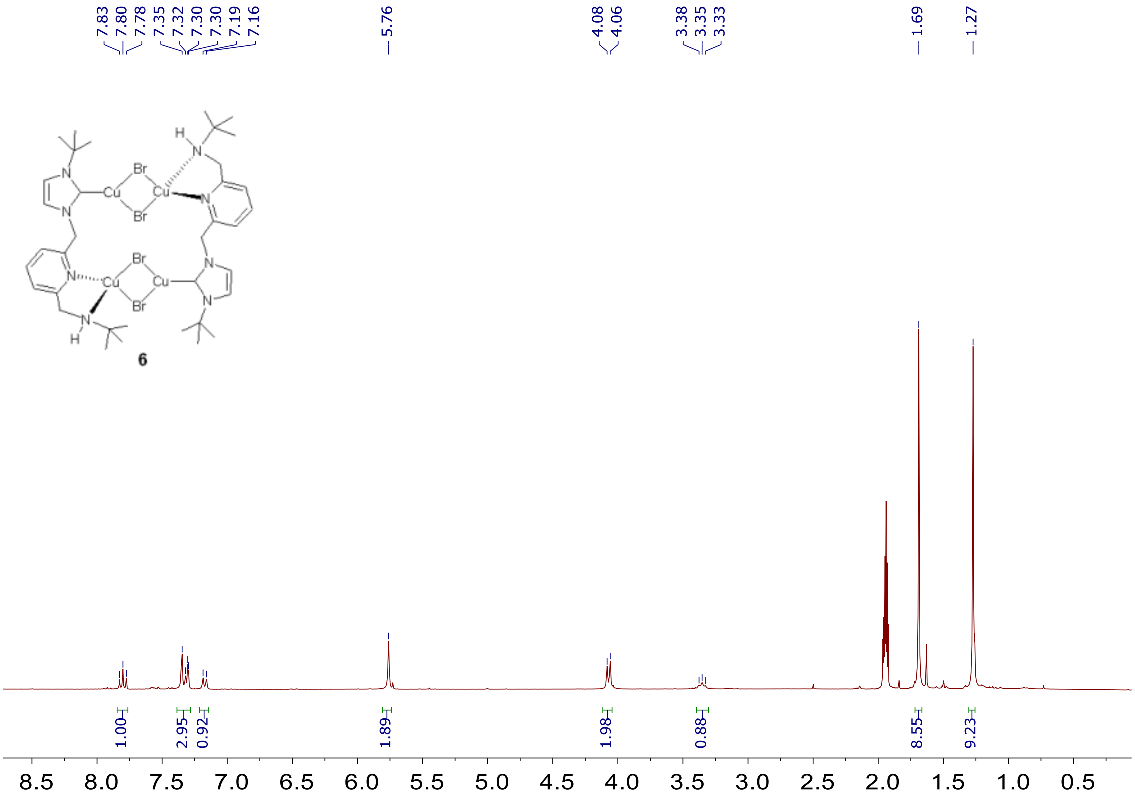

Figure S20. ^{1}H NMR (CD_3CN) of $[Cu_2(\mu\text{-}Br)_2(^{t}BuImCH_2PyCH_2NH^{t}Bu)]_n$ (**6**).

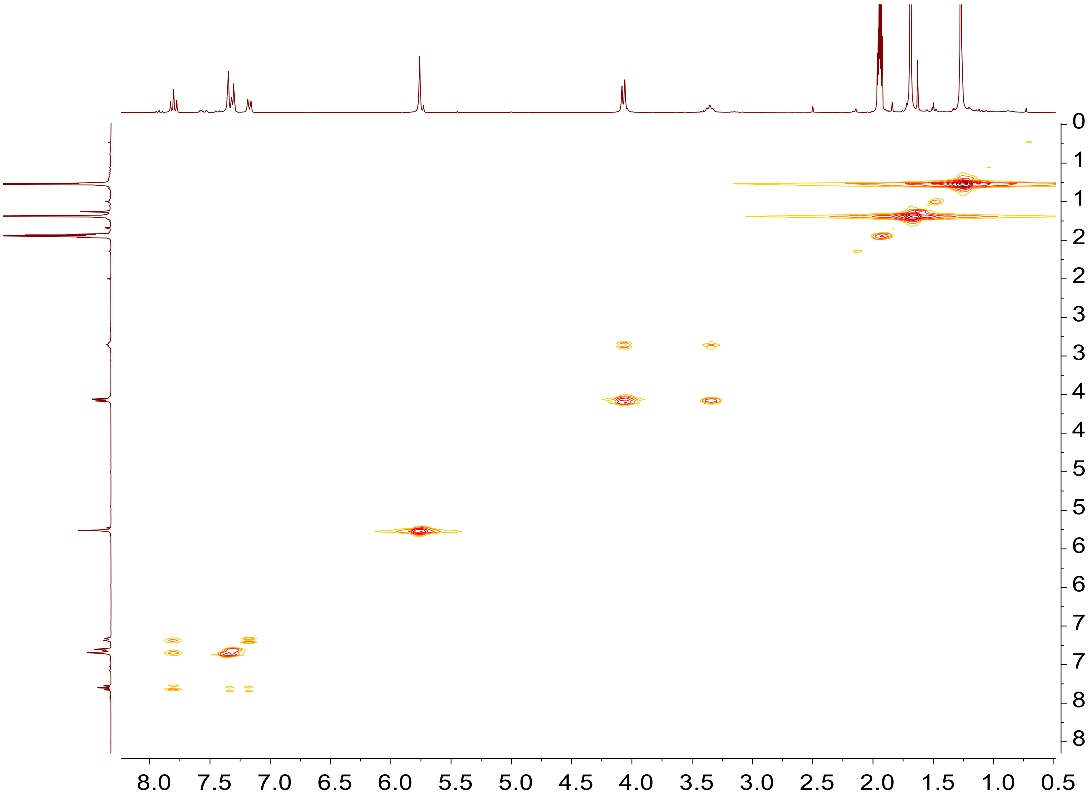

Figure S21. $^{1}H/^{1}H$-cosy NMR (CD_3CN) of $[Cu_2(\mu\text{-}Br)_2(^{t}BuImCH_2PyCH_2NH^{t}Bu)]_n$ (**6**).

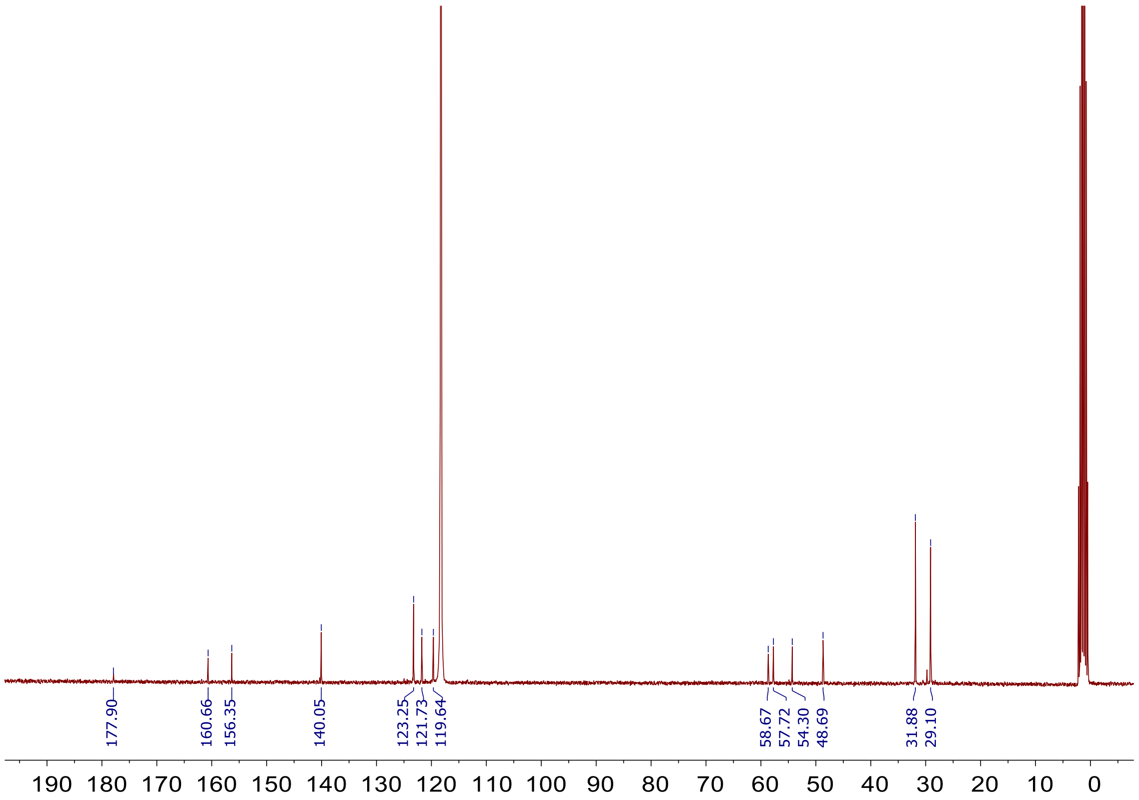

Figure S22. $^{13}C\{^{1}H\}$ NMR (CD_3CN) of [Cu_2(μ-Br)$_2$(^{t}BuImCH$_2$PyCH$_2$NH^{t}Bu)]$_n$ (**6**).

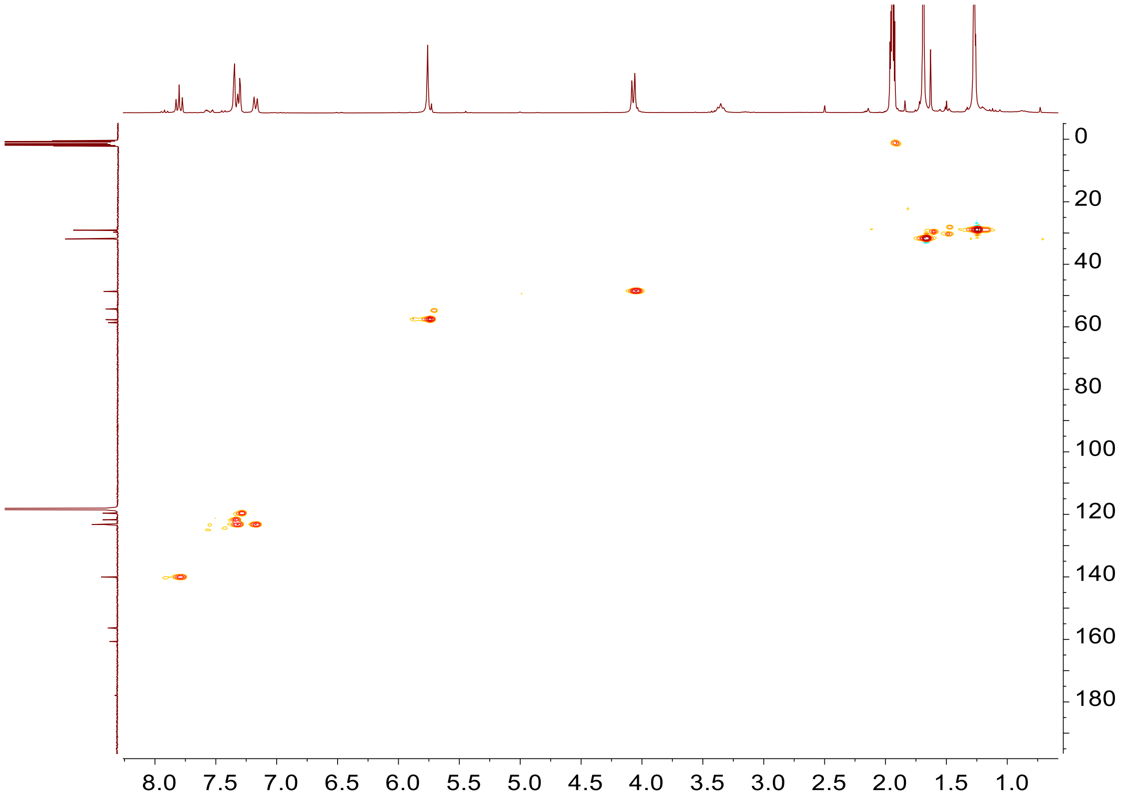

Figure S23. $^{1}H/^{13}C$-hsqc NMR (CD_3CN) of [Cu_2(μ-Br)$_2$(^{t}BuImCH$_2$PyCH$_2$NH^{t}Bu)]$_n$ (**6**).

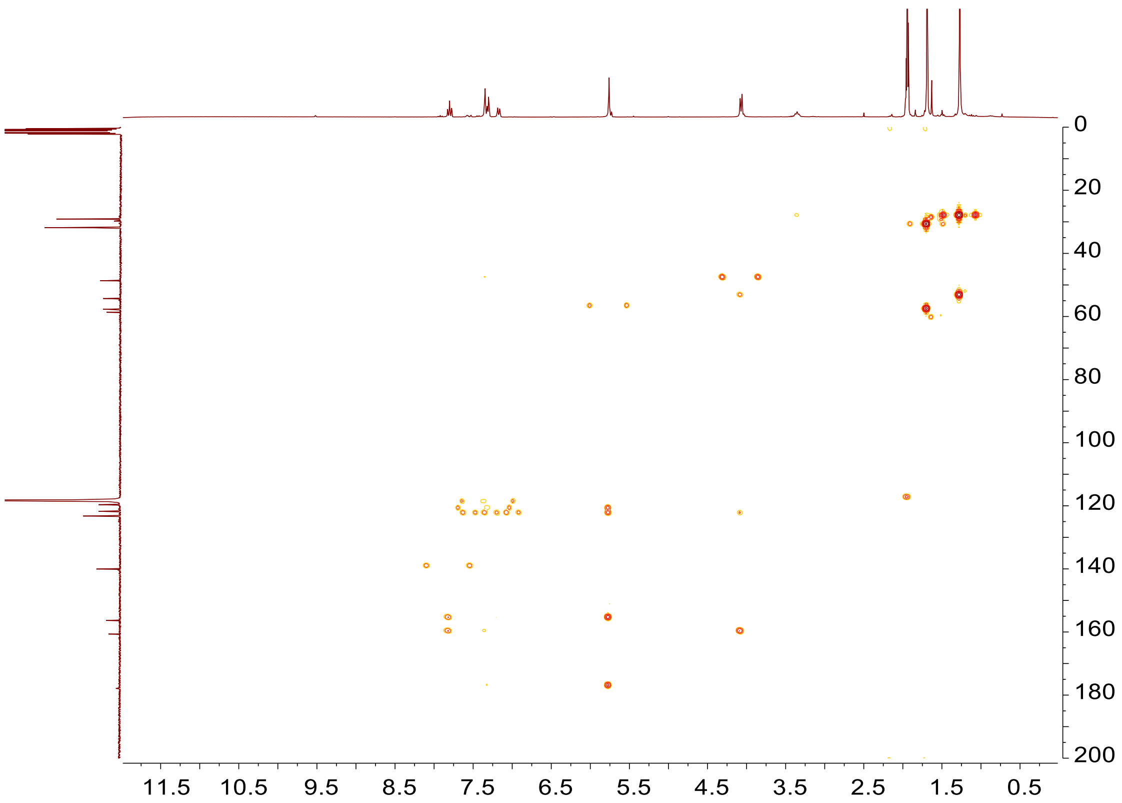

Figure S24. $^{1}H/^{13}C$-hmbc NMR (CD_3CN) of $[Cu_2(\mu\text{-}Br)_2(^{t}BuImCH_2PyCH_2NH^{t}Bu)]_n$ (**6**).

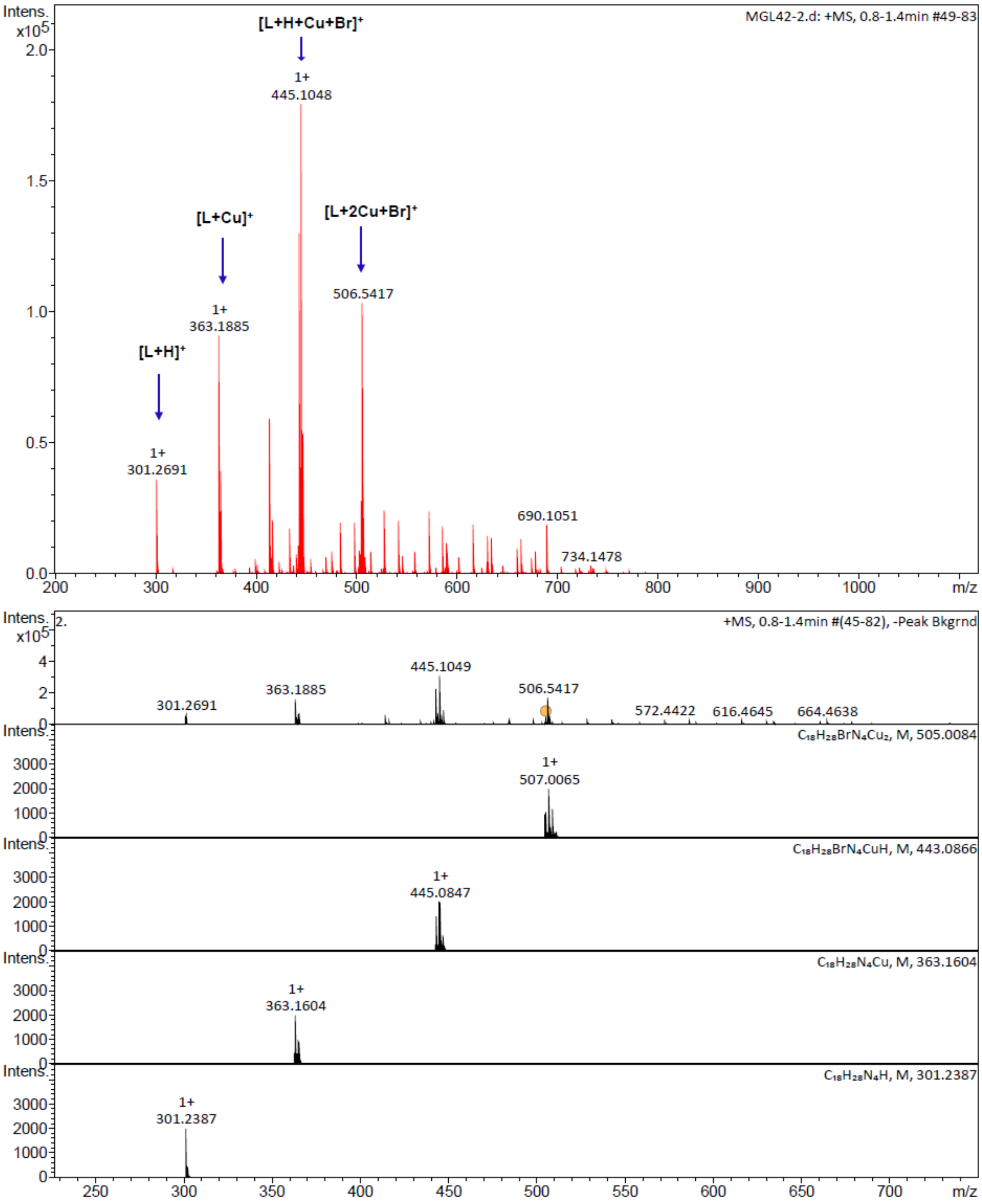

Figure S25. HRMS (ESI+, CH_3CN, m/z) of $[Cu_2(\mu\text{-}Br)_2(^tBuImCH_2PyCH_2NH^tBu)]_n$ (**6**): 506.5417 $[L+Cu_2+Br]$, 445.1048 $[L+H+Cu+Br]^+$, 363.1885 $[L+Cu]^+$, 301.2691 $[L+H]^+$.

3.- Formation of [(CuBr)$_2$(C≡CPh)(^{t}BuHImCH$_2$pyCH$_2$NEt$_2$)] (10).

*3.1 Cycloaddition of benzyl azide and phenylacetylene catalyzed by [Cu$_2$(μ-Br)$_2$(^{t}BuImCH$_2$pyCH$_2$NEt$_2$)]$_2$ (**5**) at high catalyst loading.*

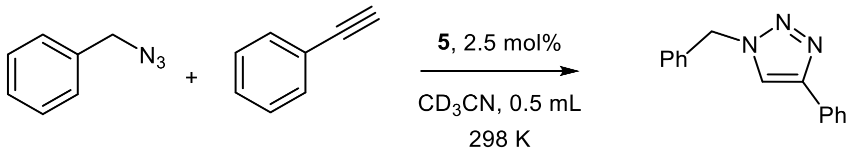

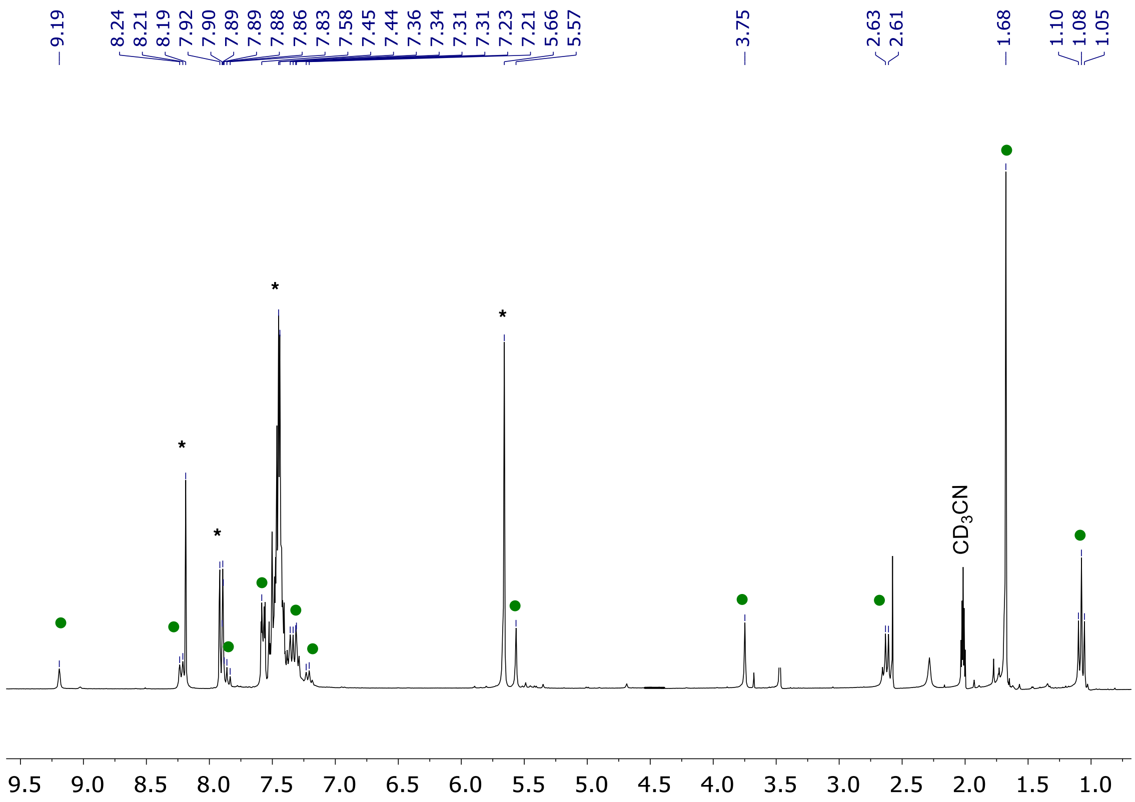

Figure S26. ^{1}H NMR (298 K, 300 MHz, CD$_3$CN) of the catalytic reaction showing the formation of [(CuBr)$_2$(C≡CPh)(^{t}BuHImCH$_2$pyCH$_2$NEt$_2$)] (**10**) (●) and the reaction product 1-benzyl-4-phenyl-1*H*-1,2,3-triazole (*).

*3.2 Reaction of [$Cu_2(\mu$-$Br)_2$(^{t}BuImCH$_2$pyCH$_2$NEt$_2$)]$_2$ (**5**) with phenylacetylene at 243 K.*

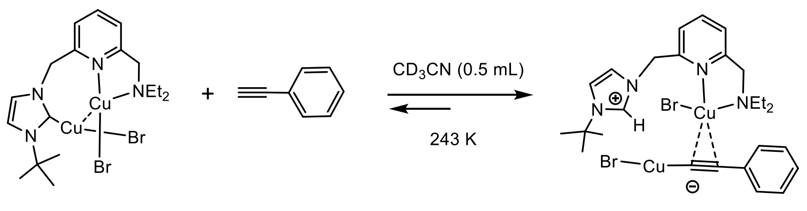

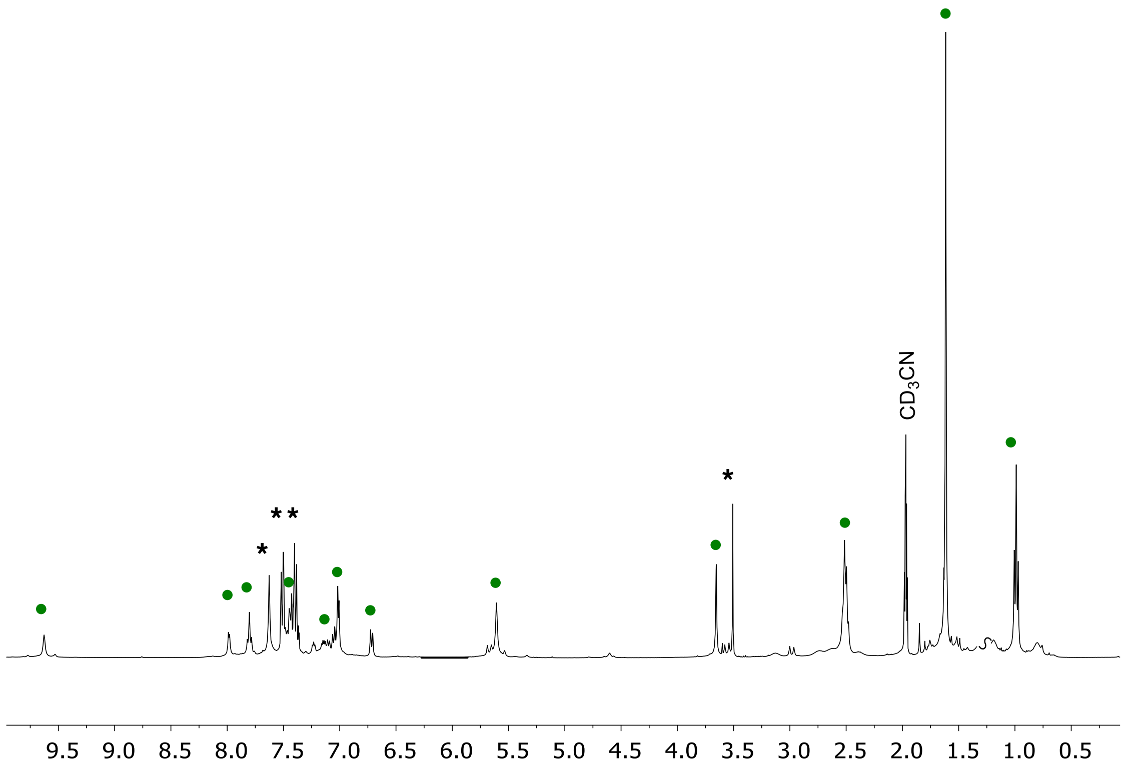

Figure S27. ^{1}H NMR (243 K, 400 MHz, CD_3CN) of the reaction of **5** with HC≡CPh (1.2 equiv) showing the presence of [$(CuBr)_2$(C≡CPh)(^{t}BuHImCH$_2$pyCH$_2$NEt$_2$)] (**10**) and phenylacetylene in excess (*).

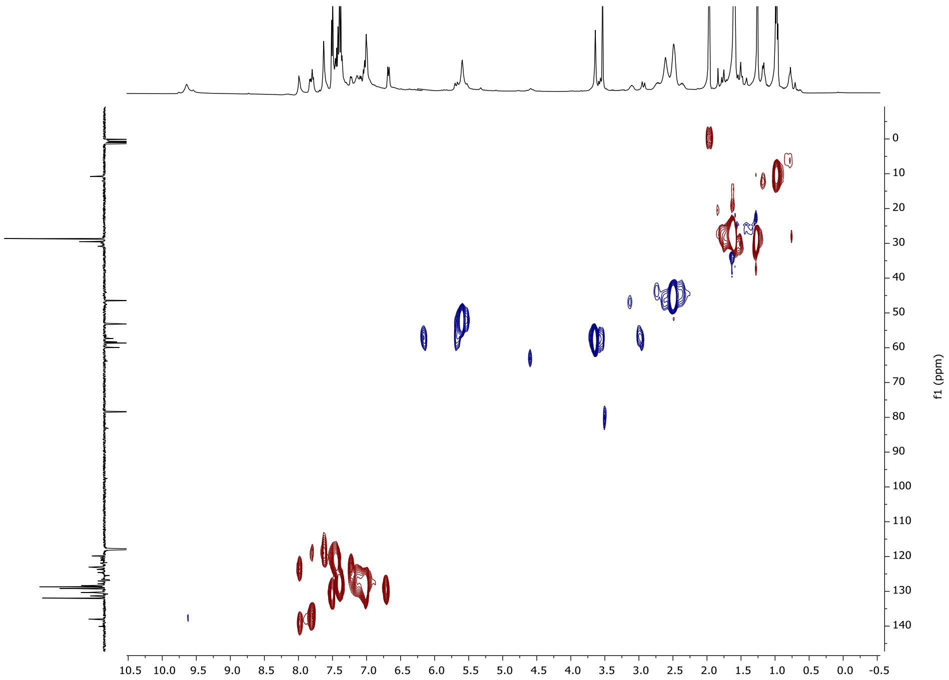

Figure S28. $^{1}H/^{13}C$-hsqc NMR (243 K, CD_3CN) of the reaction of **5** with HC≡CPh (1.2 equiv).

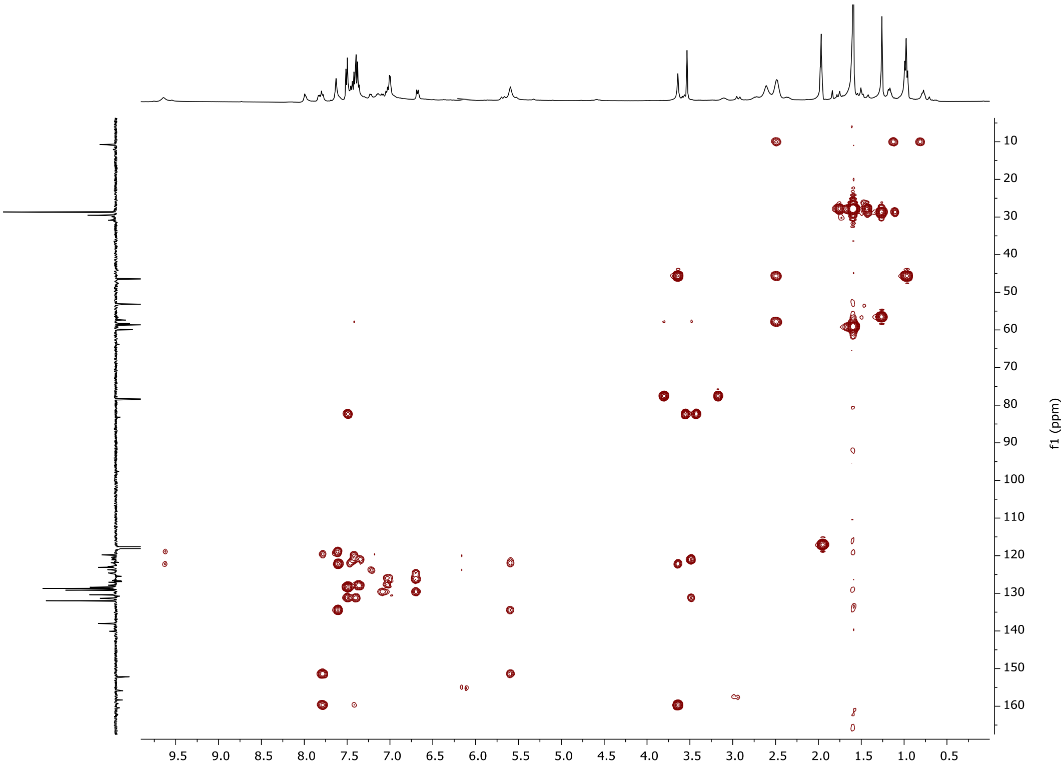

Figure S29. $^{1}H/^{13}C$-HMBC NMR (243 K, CD_3CN) of the reaction of **5** with HC≡CPh (1.2 equiv).

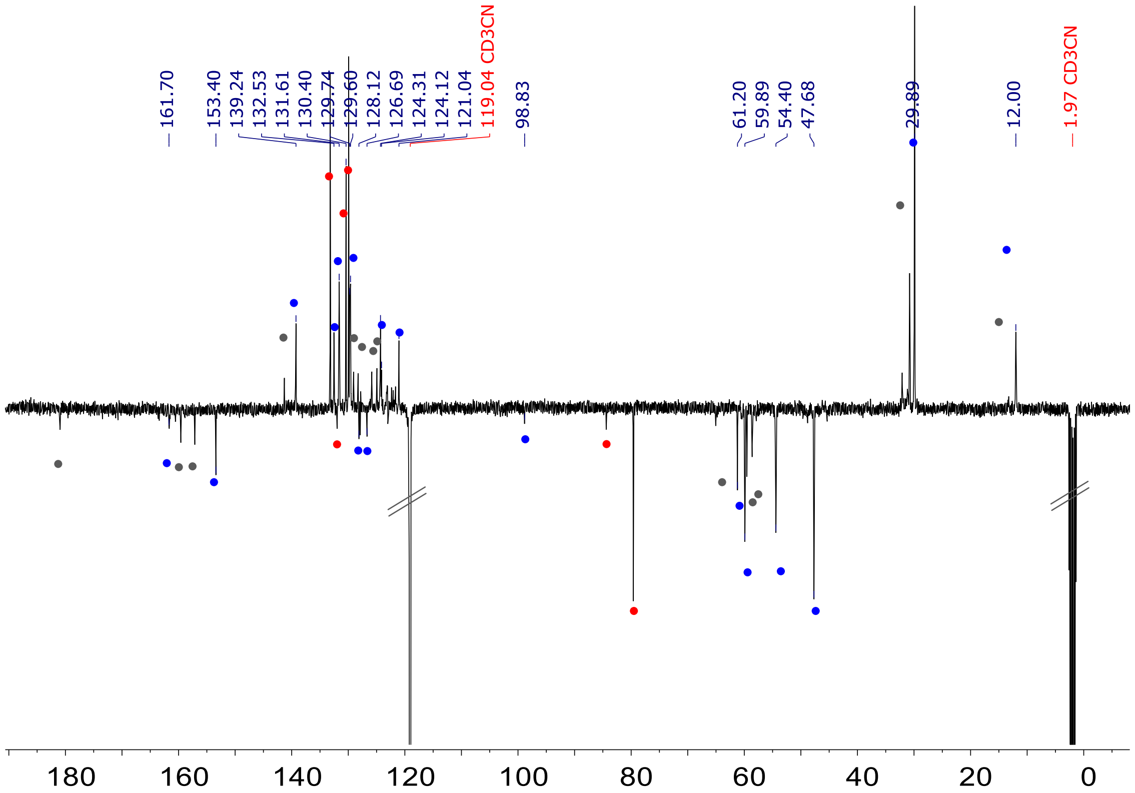

Figure S30. $^{13}C\{^{1}H\}$-APT NMR (243 K, 100.0 MHz, CD_3CN) of the reaction of **5** with HC≡CPh (5.0 equiv): [$(CuBr)_2$(C≡CPh)(^{t}BuHImCH$_2$pyCH$_2$NEt$_2$)] (**10**) (•), **5** (•) and phenylacetylene in excess (•).

4.- Isolation and characterization of 1,4-substituted 1,2,3-triazole compounds.

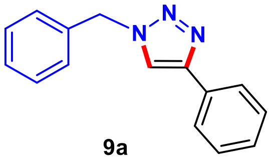

9a

5 min, 100% (98)

1-benzyl-4-phenyl-1*H*-1,2,3-triazole.[1] The general procedure was followed with benzyl azide (0.5 mmol, 62 μL) and phenylacetylene (0.5 mmol, 55 μL). Once the reaction has finished, the mixture was brought to dryness under vacuum and the residue washed with n-pentane (2 x 2 mL) yielding a white solid (115 mg, 98%). HRMS (ESI+, MeOH, m/z): 236.1189 $[M+H]^+$. ^{1}H-NMR (298 K, 300 MHz, $CDCl_3$): δ 7.90–7.77 (m, 2H), 7.46–7.26 (m, 9H), 5.58 (d, 2H).

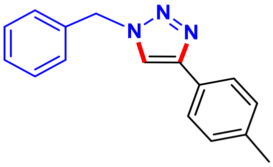

9b, 5 min, 100 (96)%

1-benzyl-4-(4-methylphenyl)-1*H*-1,2,3-triazole.[2] The general procedure was followed with benzyl azide (0.5 mmol, 62 μL) and 1-ethynyl-4-methylbenzene (0.5 mmol, 63 μL). Once the reaction has finished, the mixture was brought to dryness under vacuum and the residue washed with n-pentane (2 x 2 mL) yielding a white solid (120 mg, 96%). HRMS (ESI+, MeOH, m/z): 250.1334 $[M+H]^+$. ^{1}H-NMR (298 K, 300 MHz, $CDCl_3$): δ 7.69 (d, $J_{H\text{-}H}$ = 8.1 Hz, 2H), 7.62 (s, 1H), 7.43–7.28 (m, 5H), 7.21 (d, $J_{H\text{-}H}$ = 7.9 Hz, 2H), 5.57 (s, 2H), 2.36 (s, 3H).

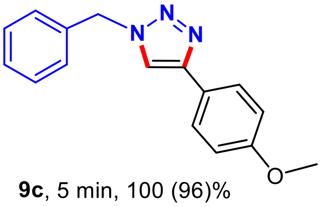

9c, 5 min, 100 (96)%

1-Benzyl-4-(4-methoxyphenyl)-1*H*-1,2,3-triazole.[2] The general procedure was followed with benzyl azide (0.5 mmol, 62 μL) and 1-ethynyl-4-methoxybenzene (0.5 mmol, 65 μL). Once the reaction has finished, the mixture was brought to dryness under vacuum and the residue washed with n-pentane (2 x 2 mL) yielding a white solid (127 mg, 96%). HRMS (ESI+, MeOH, m/z): 266.1289 $[M+H]^+$. ^{1}H-NMR (298 K, 300 MHz, $CDCl_3$): 7.75–7.68 (m, 2H), 7.58 (s, 1H), 7.43–7.27 (m, 5H), 6.96–6.88 (m, 2H), 5.55 (s, 2H), 3.82 (s, 3H).

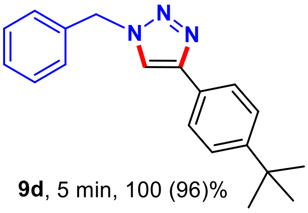

9d, 5 min, 100 (96)%

1-benzyl-(4-*t*-butylphenyl)-1*H*-1,2,3-triazole.[3] The general procedure was followed with with benzyl azide (0.5 mmol, 62 μL) and 1-(*t*-butyl)-4-ethynylbenzene (0.5 mmol, 90 μL). Once the reaction has finished, the mixture was brought to dryness under vacuum and the residue washed with n-pentane (2 x 2 mL) yielding a white solid (140 mg, 96%). HRMS (ESI+, MeOH, m/z): 292.1802 $[M+H]^+$.

^{1}H-NMR (298 K, 300 MHz, $CDCl_3$): δ 7.85 (br, 2H), 7.50–7.27 (m, 8H), 5.58 (s, 2H), 1.34 (s, 9H).

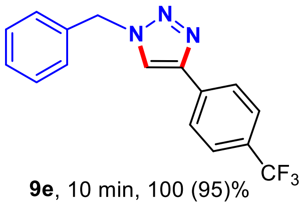

9e, 10 min, 100 (95)%

1-benzyl-4-(4-(trifluoromethyl)phenyl)-1*H*-1,2,3-triazole.[4] The general procedure was followed using benzyl azide (0.5 mmol, 62 μL) and 1-ethynyl-4-(trifluoromethyl)benzene (0.5 mmol, 81 μL). Once the reaction has finished, the mixture was brought to dryness under vacuum and the residue washed with n-pentane (2 x 2 mL) yielding a white solid (146 mg, 96%). HRMS (ESI+, MeOH, m/z): 304.1056 $[M+H]^+$. ^{1}H-NMR (298 K, 300 MHz, $CDCl_3$): δ 7.92 (d, J_{H-H} = 8.0 Hz, 2H), 7.75 (s, 1H), 7.65 (d, J_{H-H} = 8.0 Hz, 2H), 7.47–7.28 (m, 5H), 5.60 (s, 2H).

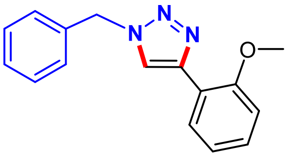

9f, 5 min, 100 (99)%

1-benzyl-4-(2-methoxyphenylphenyl)-1*H*-1,2,3-triazole.[5] The general procedure was followed using benzyl azide (0.5 mmol, 62 μL) and 1-ethynyl-2-methoxybenzene (0.5 mmol, 65 μL). Once the reaction has finished, the mixture was brought to dryness under vacuum and the residue washed with n-pentane (2 x 2 mL) yielding a white solid (131 mg, 99%). HRMS (ESI+, MeOH, m/z): 266.1278 $[M+H]^+$. ^{1}H-NMR (298 K, 300 MHz, $CDCl_3$): 8.36 (dd, J_{H-H} = 7.7 Hz, J_{H-H} = 1.7 Hz, 1H), 7.98 (s, 1H), 7.42–7.27 (m, 6H), 7.08 (td, J_{H-H} = 7.6 Hz, J_{H-H} = 1.0 Hz, 1H), 6.95 (d, J_{H-H} = 8.3 Hz, 1H).

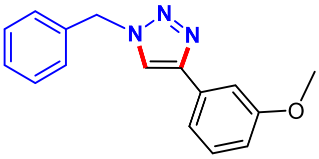

9g, 5 min, 100 (94)%

1-benzyl-4-(3-methoxyphenylphenyl)-1*H*-1,2,3-triazole.[6] The general procedure was followed using benzyl azide (0.5 mmol, 62 μL) and 1-ethynyl-3-methoxybenzene (0.5 mmol, 64 μL). Once the reaction has finished, the mixture was brought to dryness under vacuum and the residue washed with n-pentane (2 x 2 mL) yielding a white solid (125 mg, 94%). HRMS (ESI+, MeOH, m/z): 266.1278 $[M+H]^+$. ^{1}H-NMR (298 K, 300 MHz, $CDCl_3$): 7.93 (br, 1H), 7.52 (br, 1H), 7.45–7.26 (m, 7H), 6.88 (d, J_{H-H} = 7.9 Hz, 1H), 5.58 (s, 2H), 3.87 (s, 3H).

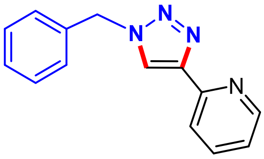

9h, 5 min, 100 (93)%

2-(1-benzyl-1*H*-1,2,3-triazol-4-yl)pyridine.[6] The general procedure was followed using benzyl azide (0.5 mmol, 62 μL) and 2-ethynyl-pyridine (0.5 mmol, 50 μL). Once the reaction has finished, the mixture was brought to dryness under vacuum and the

residue washed with n-pentane (2 x 2 mL) yielding a white solid (110 mg, 93%). HRMS (ESI+, MeOH, m/z): 259.0966 $[M+Na]^+$. ^{1}H-NMR (298 K, 300 MHz, $CDCl_3$): 8.23 (br, 3H), 7.85 (br, 1H), 7.50–7.28 (m, 6H), 5.59 (s, 2H).

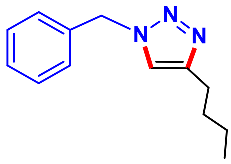

9i, 3h, 100 (95)%

1-benzyl-4-butyl-1*H*-1,2,3-triazole.[1] The general procedure was followed using benzyl azide (0.5 mmol, 62 µL) and 1-hexyne (0.5 mmol, 57 µL). Once the reaction has finished, the mixture was brought to dryness under vacuum and the residue washed with n-pentane (2 x 2 mL) yielding a white solid (102 mg, 95%). HRMS (ESI+, MeOH, m/z): 216.1504 $[M+H]^+$. ^{1}H-NMR (298 K, 300 MHz, $CDCl_3$): 7.55–7.09 (m, 6H), 5.58 (s, 2H), 2.70 (br, 2H), 1.89 (br, 2H), 1.49 (br, 2H), 1.00 (br, 3H).

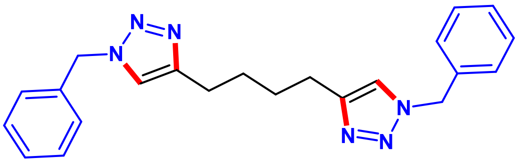

9j, 3 h, 94 (90)%

1,4-bis(1-benzyl-1*H*-1,2,3-triazol-4-yl)-butane. The general procedure was followed using benzyl azide (1.0 mmol, 124 µL) and 1-7-octadiyne (0.5 mmol, 66 µL). Once the reaction has finished, the mixture was brought to dryness under vacuum and the residue washed with n-pentane (2 x 2 mL) yielding a white solid (168 mg, 90%). HRMS (ESI+, MeOH, m/z): 373.2149 $[M+H]^+$. ^{1}H-NMR (298 K, 300 MHz, $CDCl_3$): 7.33–7.12 (m, 12H), 5.43 (s, 4H), 2.67 (br, 4H), 1.72 (br, 4H).

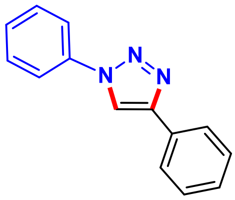

9m, 30 min, 100 (94)%

1,4-diphenyl-1*H*-1,2,3-triazole.[1] The general procedure was followed using phenyl azide (0.5 mmol, 59 µL) and phenylacetylene (0.5 mmol, 55 µL). Once the reaction has finished, the mixture was brought to dryness under vacuum and the residue washed with n-pentane (2 x 2 mL) yielding a white solid (104 mg, 94%). HRMS (ESI+, MeOH, m/z): 222.1030 $[M+H]^+$. ^{1}H-NMR (298 K, 300 MHz, $CDCl_3$): 8.37 (br, 1H), 7.97 (br, 2H), 7.87–7.71 (m, 2H), 7.60–7.33 (m, 6H).

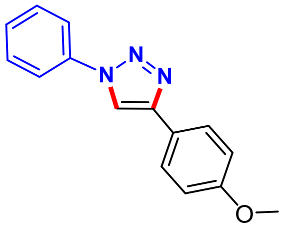

9n, 30 min, 100 (94)%

4-(4-methoxyphenyl)-1-phenyl-1*H*-1,2,3-triazole. The general procedure was followed using phenyl azide (0.5 mmol, 59 µL) and 1-ethynyl-4-methoxybenzene (0.5 mmol, 65 µL). Once the reaction has finished, the mixture was brought to dryness under vacuum and the residue washed with n-pentane (2 x 2 mL) yielding a white solid (118 mg, 94%). HRMS (ESI+, MeOH, m/z): 274.0947 $[M+Na]^+$. ^{1}H-NMR

(298 K, 300 MHz, $CDCl_3$): 8.11 (s, 1H), 7.88–7.74 (m, 4H), 7.60–7.40 (m, 3H), 7.03–6.96 (m, 2H), 3.86 (s, 3H).

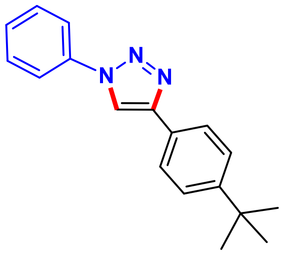

9o, 30 min, 100 (95)%

4-(4-*t*-butylphenyl)-1-phenyl-1*H*-1,2,3-triazole. The general procedure was followed using phenyl azide (0.5 mmol, 59 µL) and 1-(*t*-butyl)-4-ethynylbenzene (0.5 mmol, 90 µL). Once the reaction has finished, the mixture was brought to dryness under vacuum and the residue washed with n-pentane (2 x 2 mL) yielding a pale yellow solid (131 mg, 95%). HRMS (ESI+, MeOH, m/z): 278.1652 $[M+H]^+$. ^{1}H-NMR (298 K, 300 MHz, $CDCl_3$): 8.39 (br, 1H), 8.13–7.69 (m, 4H), 7.66–7.34 (m, 5H), 1.37 (s, 9H).

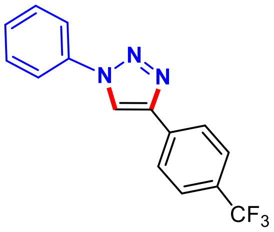

9p, 30 min, 94 (89)%

4-(4-(trifluoromethyl)phenyl)-1-phenyl-1*H*-1,2,3-triazole. The general procedure was followed using phenyl azide (0.5 mmol, 59 µL) and 1-ethynyl-4-(trifluoromethyl)benzene (0.5 mmol, 81 µL). Once the reaction has finished, the mixture was brought to dryness under vacuum and the residue washed with n-pentane (2 x 2 mL) yielding a pale-yellow solid (129 mg, 89%). HRMS (ESI+, MeOH, m/z): 312.0720 $[M+Na]^+$. ^{1}H-NMR (298 K, 300 MHz, $CDCl_3$): 8.34–8.25 (m, 1H), 8.14–8.00 (m, 2H), 7.90–7.69 (m, 4H), 7.67–7.45 (m, 3H).

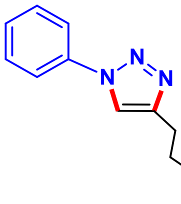

9q, 30 min, 100 (90)%

4-butyl-1-phenyl-1*H*-1,2,3-triazole.[1] The general procedure was followed using phenyl azide (0.5 mmol, 59 µL) and 1-hexyne (0.5 mmol, 57 µL). Once the reaction has finished, the mixture was brought to dryness under vacuum and the residue washed with n-pentane (2 x 2 mL) yielding a pale-yellow solid (90 mg, 90%). HRMS (ESI+, MeOH, m/z): 202.1344 $[M+H]^+$. ^{1}H-NMR (298 K, 300 MHz, $CDCl_3$): 8.43 (br, 1H), 7.90–7.69 (m, 2H), 7.60–7.34 (m, 3H), 2.81 (br, 2H), 1.82 (br, 2H), 1.47 (br, 2H), 0.99 (br, 3H).

5.- NMR spectra of 1,4-disubstituted-1,2,3-triazole compounds.

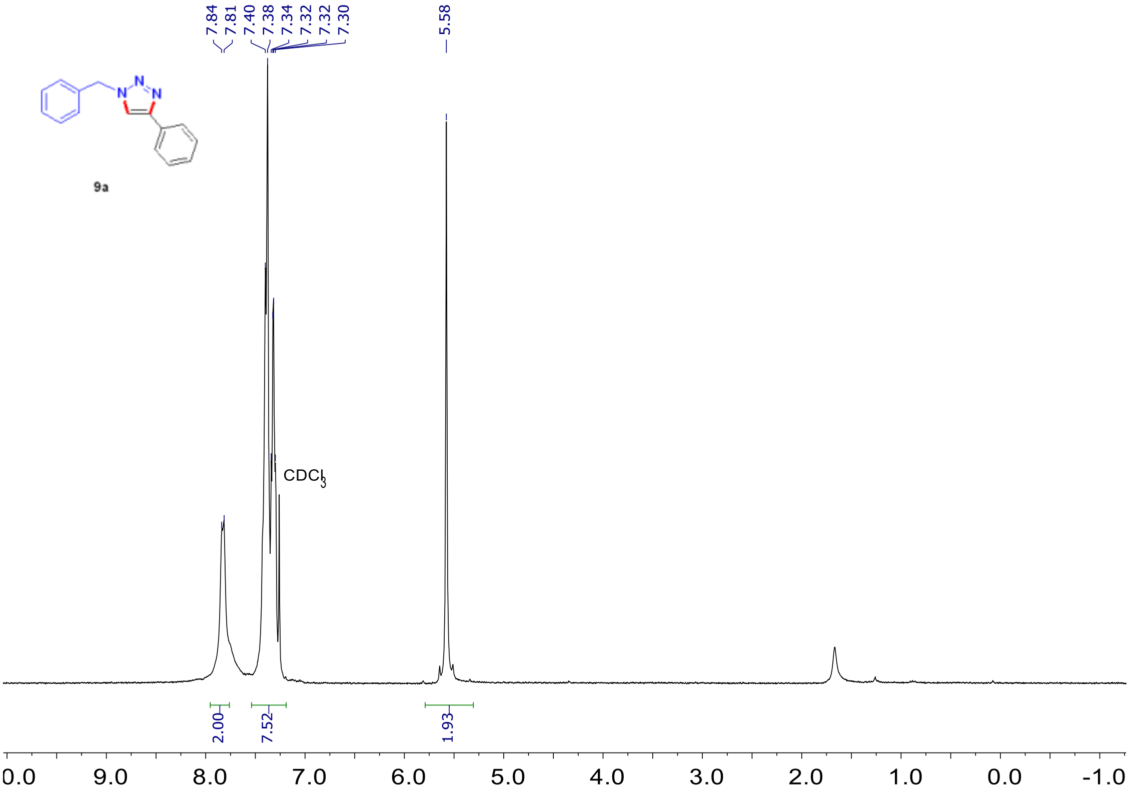

Figure S31. ^{1}H NMR ($CDCl_3$, 300 MHz) of **9a**.

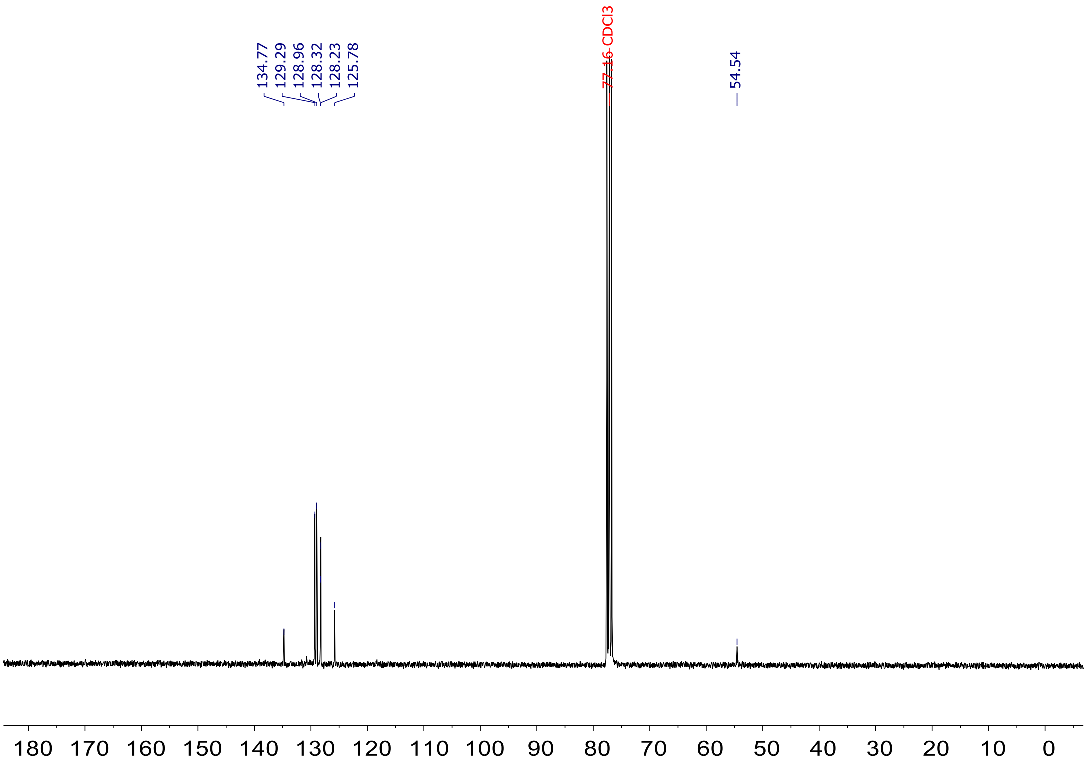

Figure S32. $^{13}C\{^{1}H\}$ NMR ($CDCl_3$, 75 MHz) of **9a**.

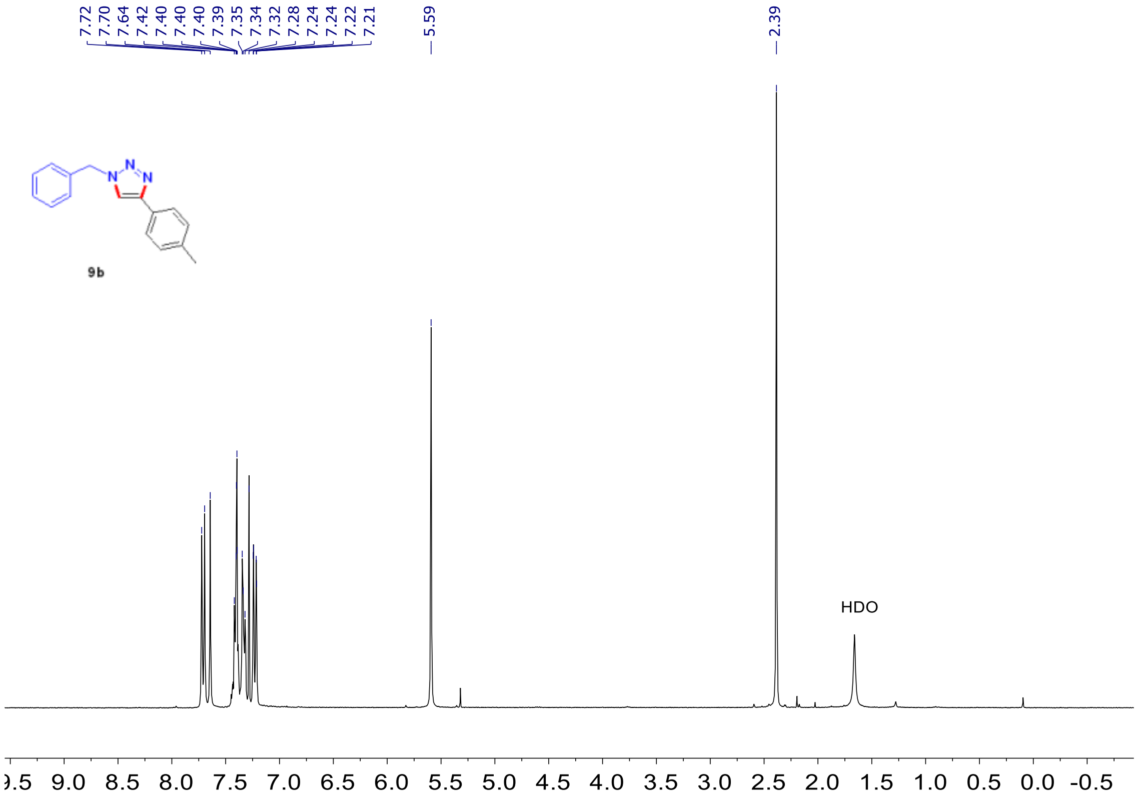

Figure S33. ^{1}H NMR ($CDCl_3$, 300 MHz) of **9b**.

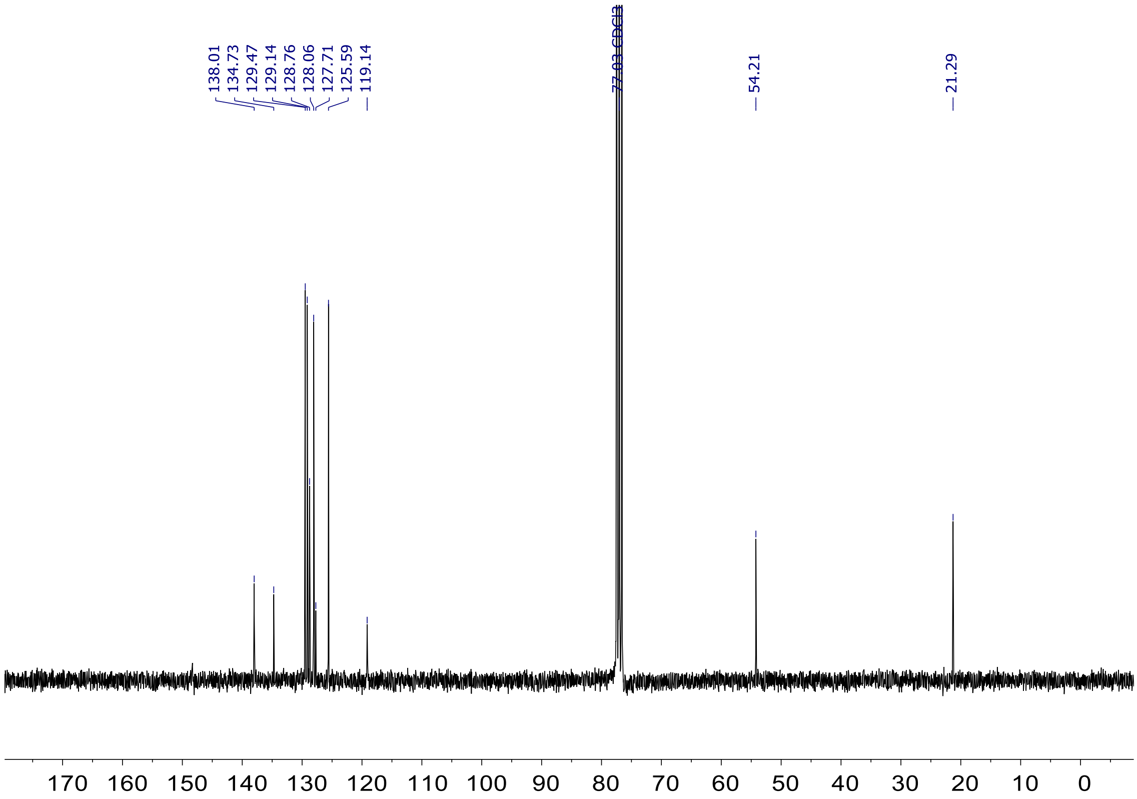

Figure S34. $^{13}C\{^{1}H\}$ NMR ($CDCl_3$, 75 MHz) of **9b**.

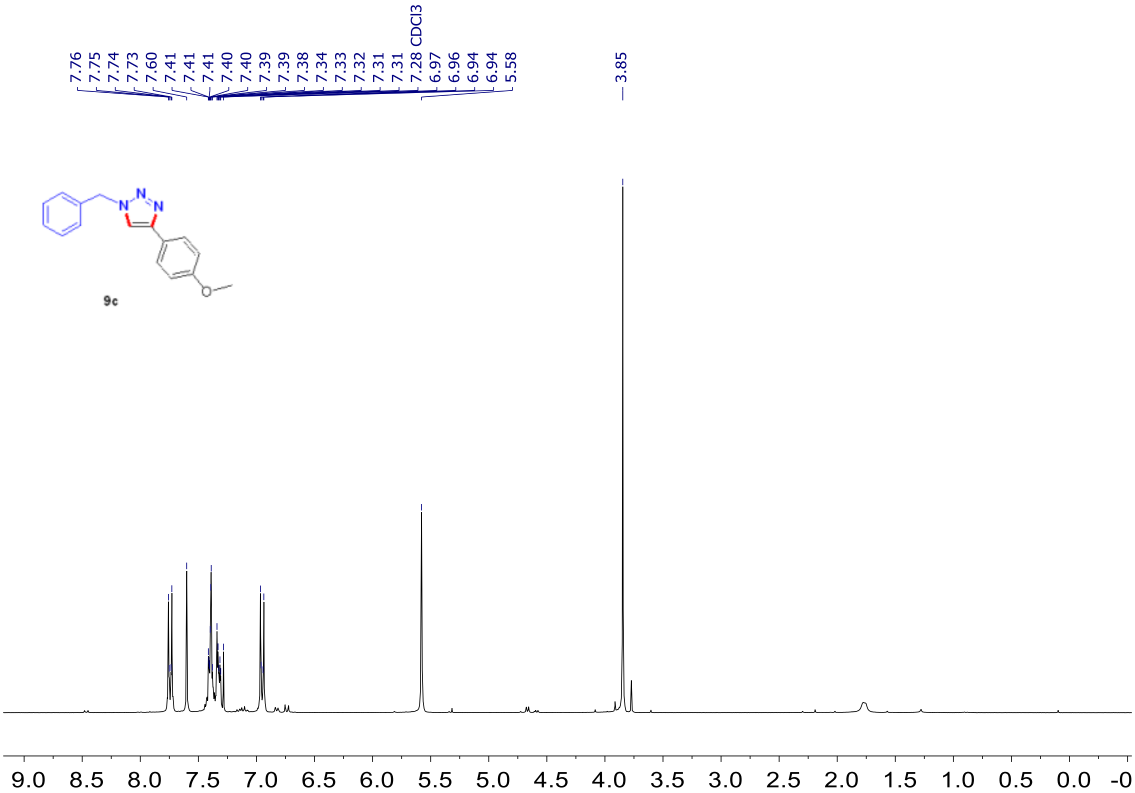

Figure S35. ^{1}H NMR ($CDCl_3$, 300 MHz) of **9c**.

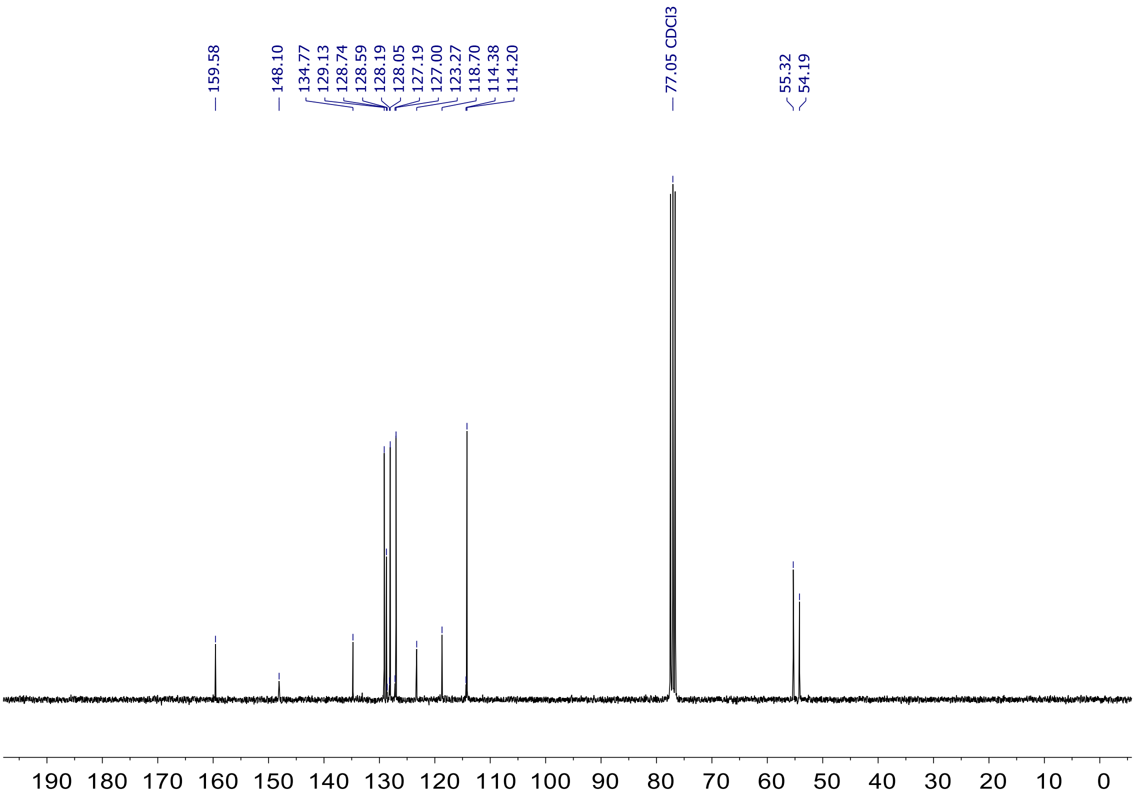

Figure S36. $^{13}C\{^{1}H\}$ NMR ($CDCl_3$, 75 MHz) of **9c**.

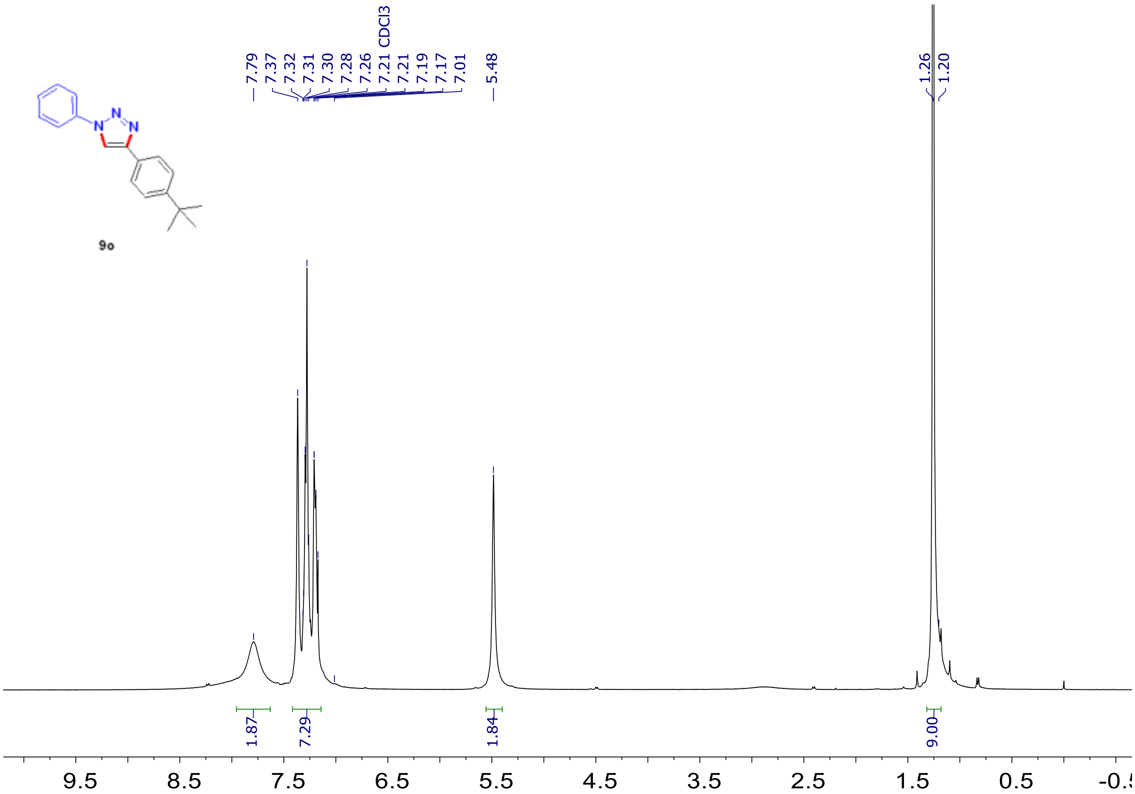

Figure S37. ^{1}H NMR ($CDCl_3$, 300 MHz) of **9d**.

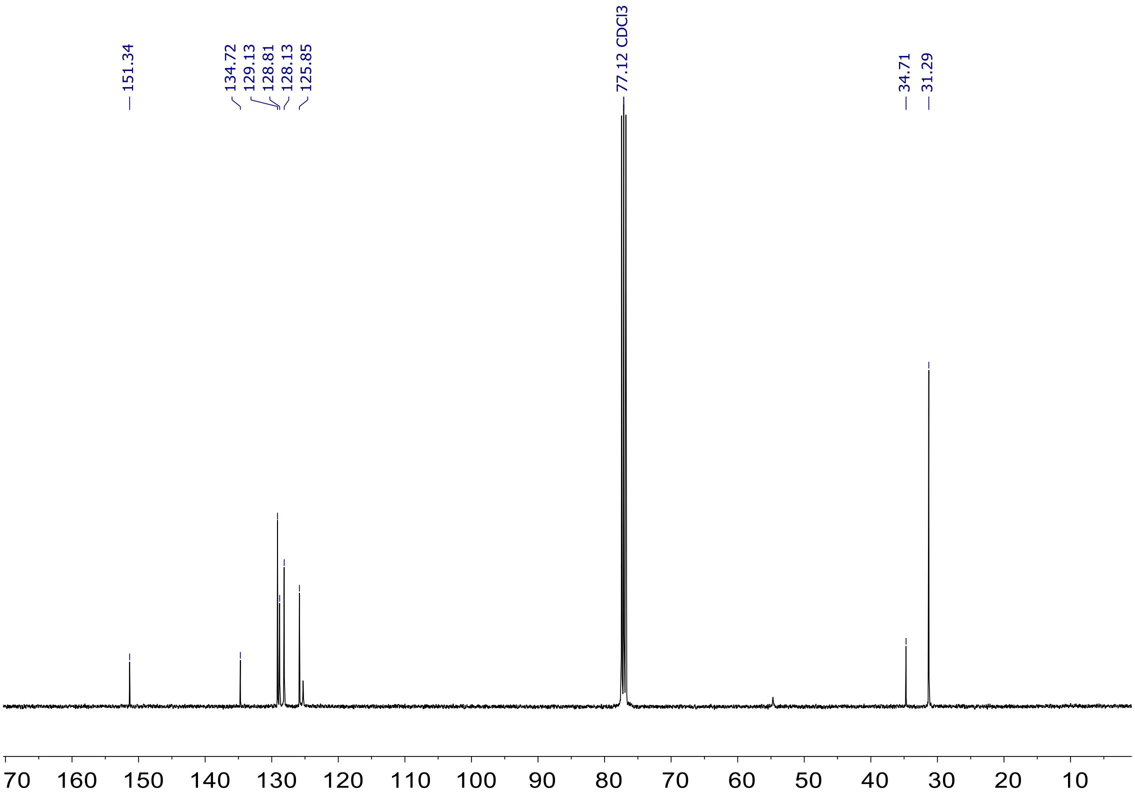

Figure S38. $^{13}C\{^{1}H\}$ NMR ($CDCl_3$, 75 MHz) of **9d**.

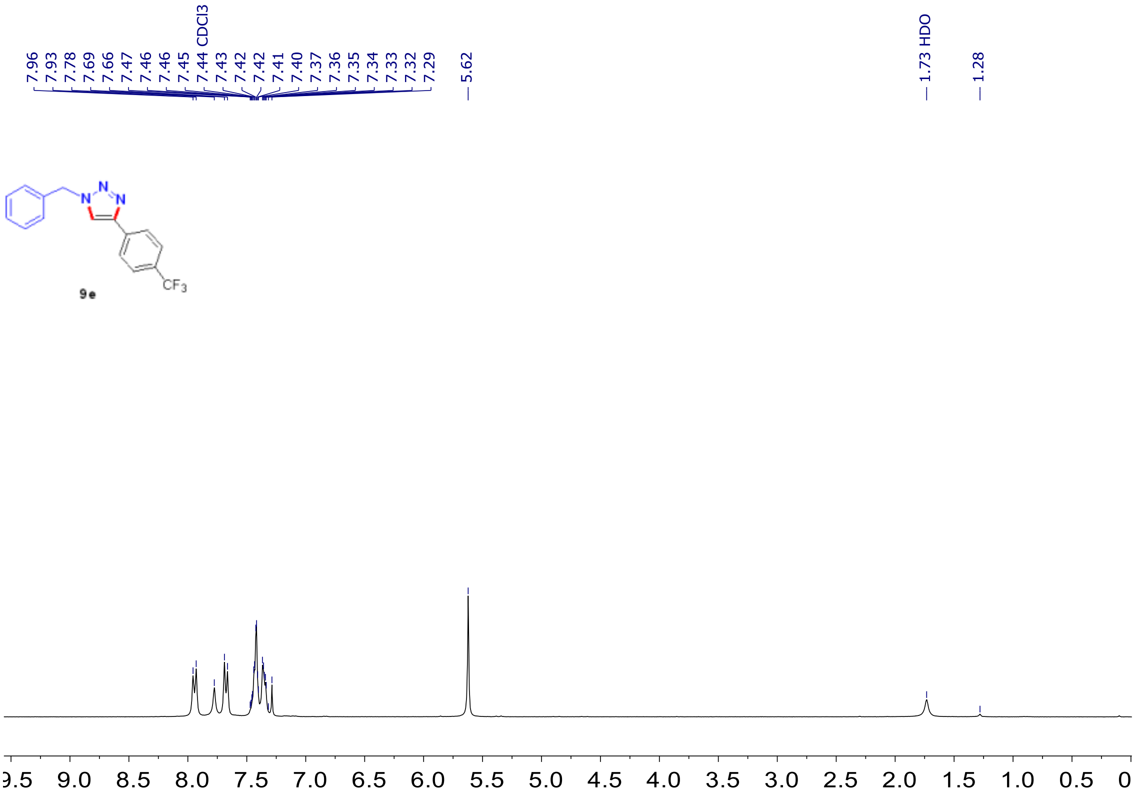

Figure S39. ^{1}H NMR ($CDCl_3$, 300 MHz) of **9e**.

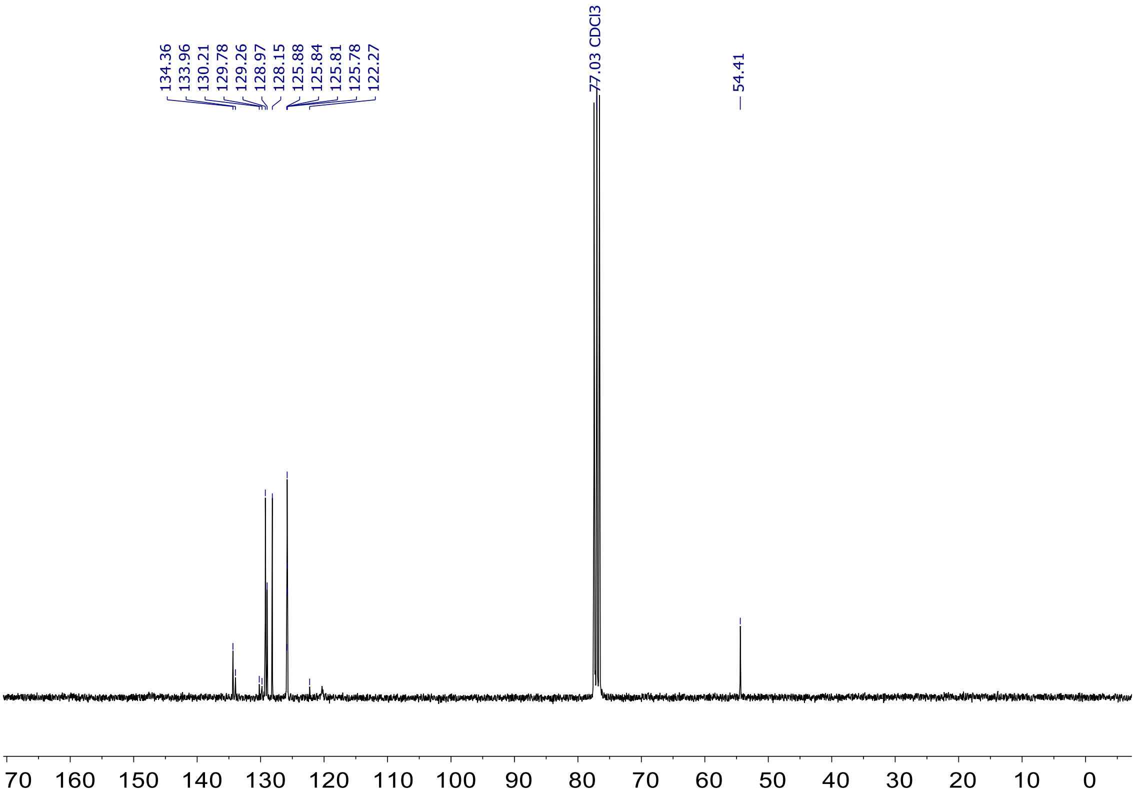

Figure S40. $^{13}C\{^{1}H\}$ NMR ($CDCl_3$, 75 MHz) of **9e**.

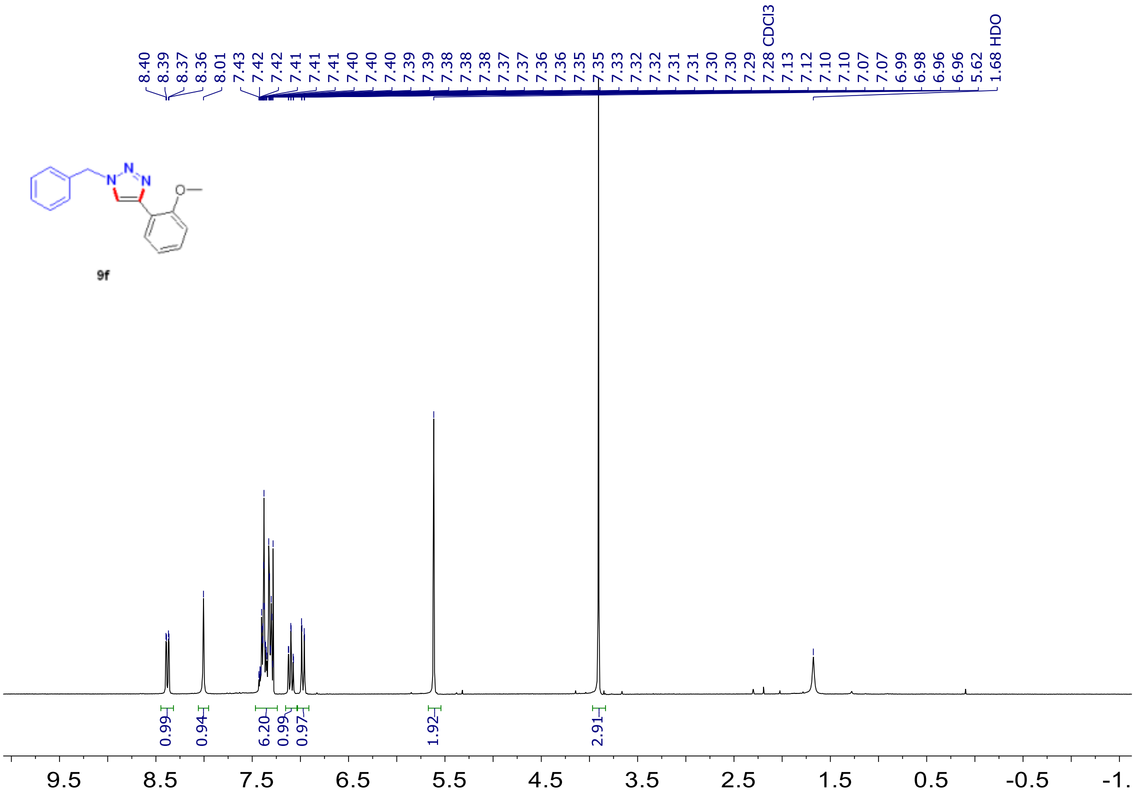

Figure S41. ^{1}H NMR ($CDCl_3$, 300 MHz) of **9f**.

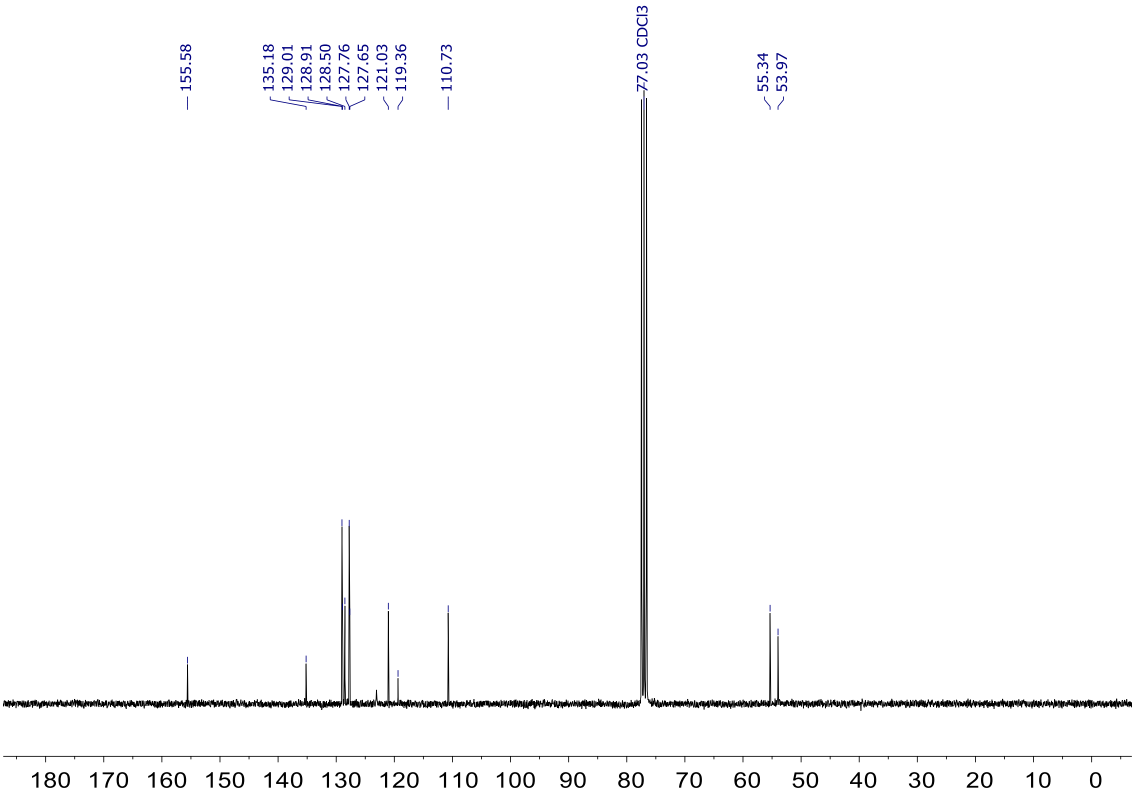

Figure S42. $^{13}C\{^{1}H\}$ NMR ($CDCl_3$, 75 MHz) of **9f**.

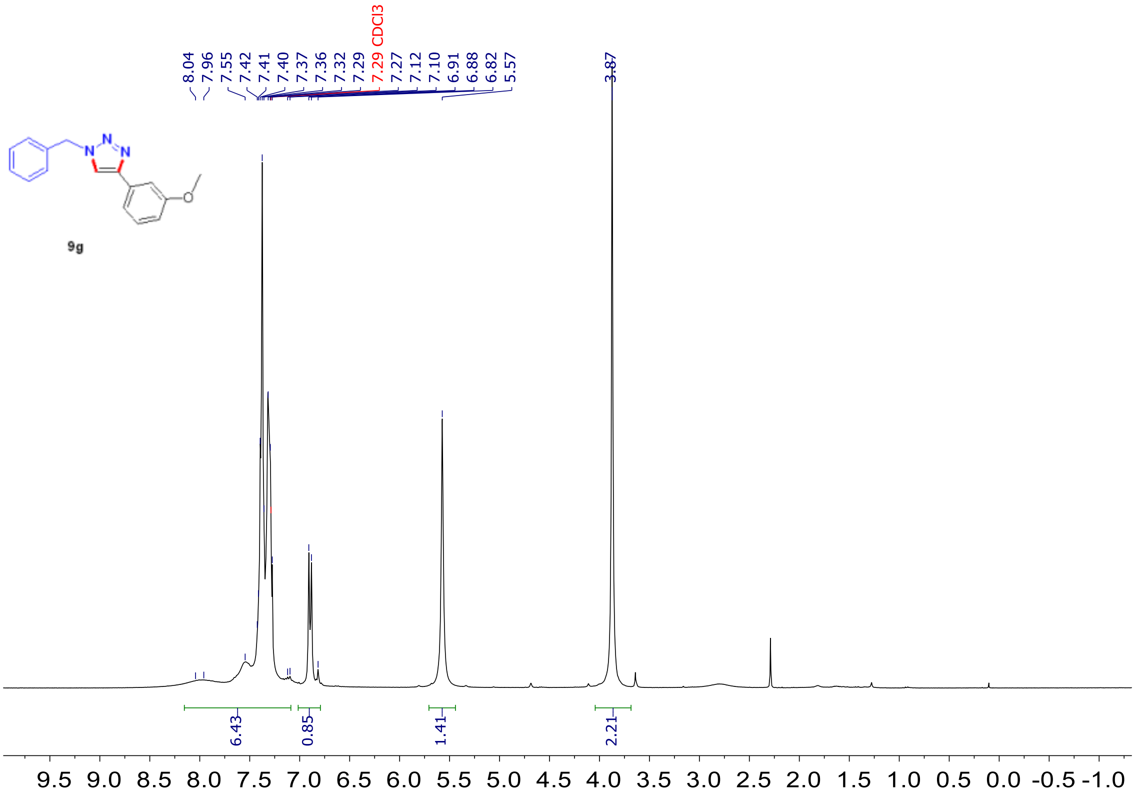

Figure S43. ^{1}H NMR ($CDCl_3$, 300 MHz) of **9g**.

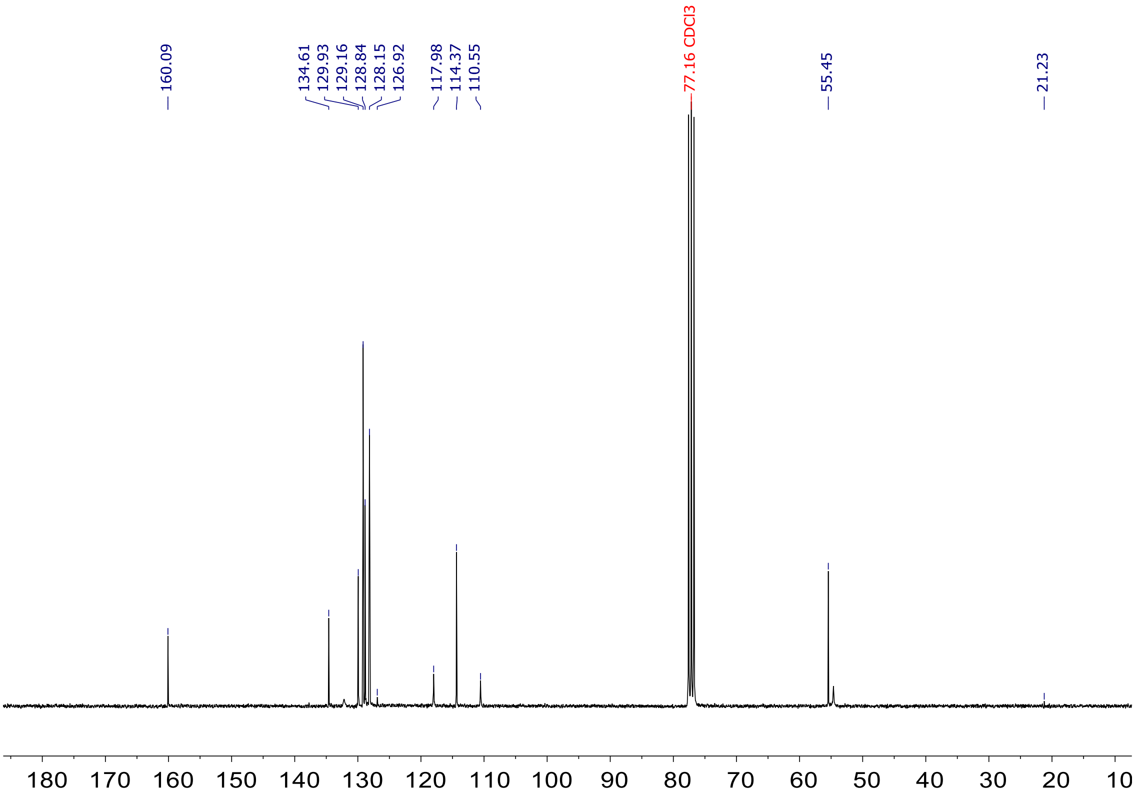

Figure S44. $^{13}C\{^{1}H\}$ NMR ($CDCl_3$, 75 MHz) of **9g**.

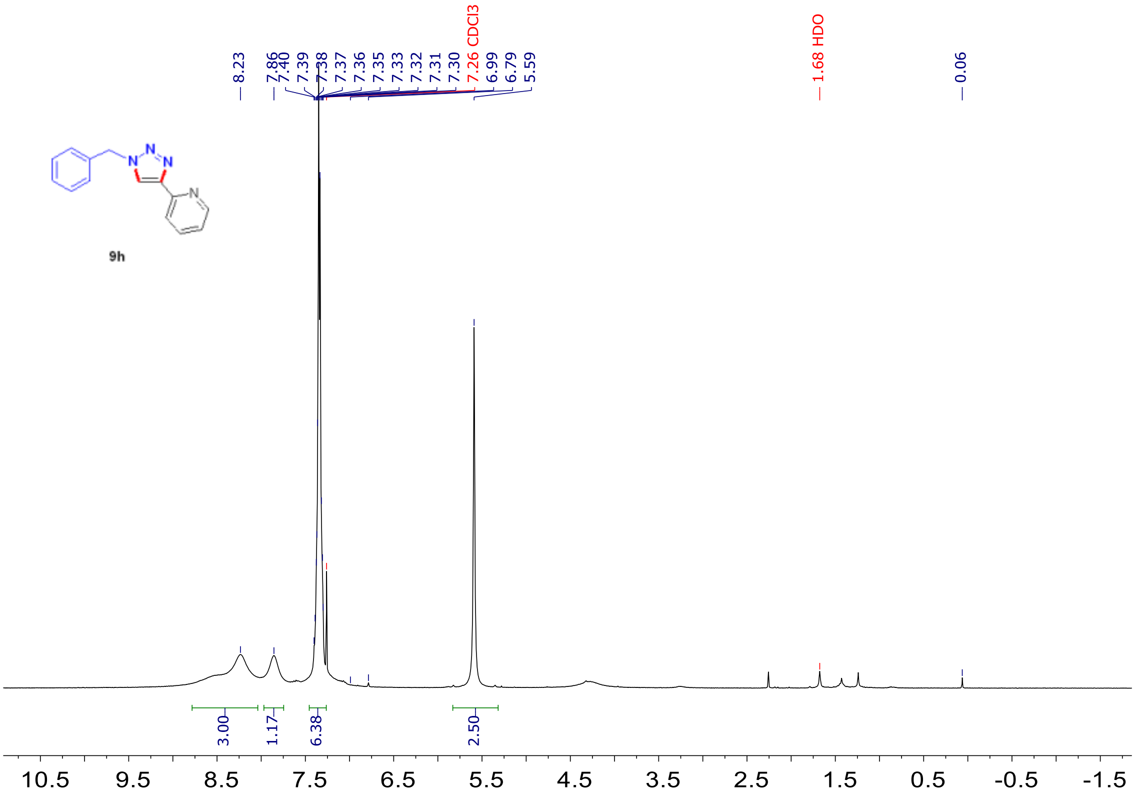

Figure S45. ^{1}H NMR ($CDCl_3$, 300 MHz) of **9h**.

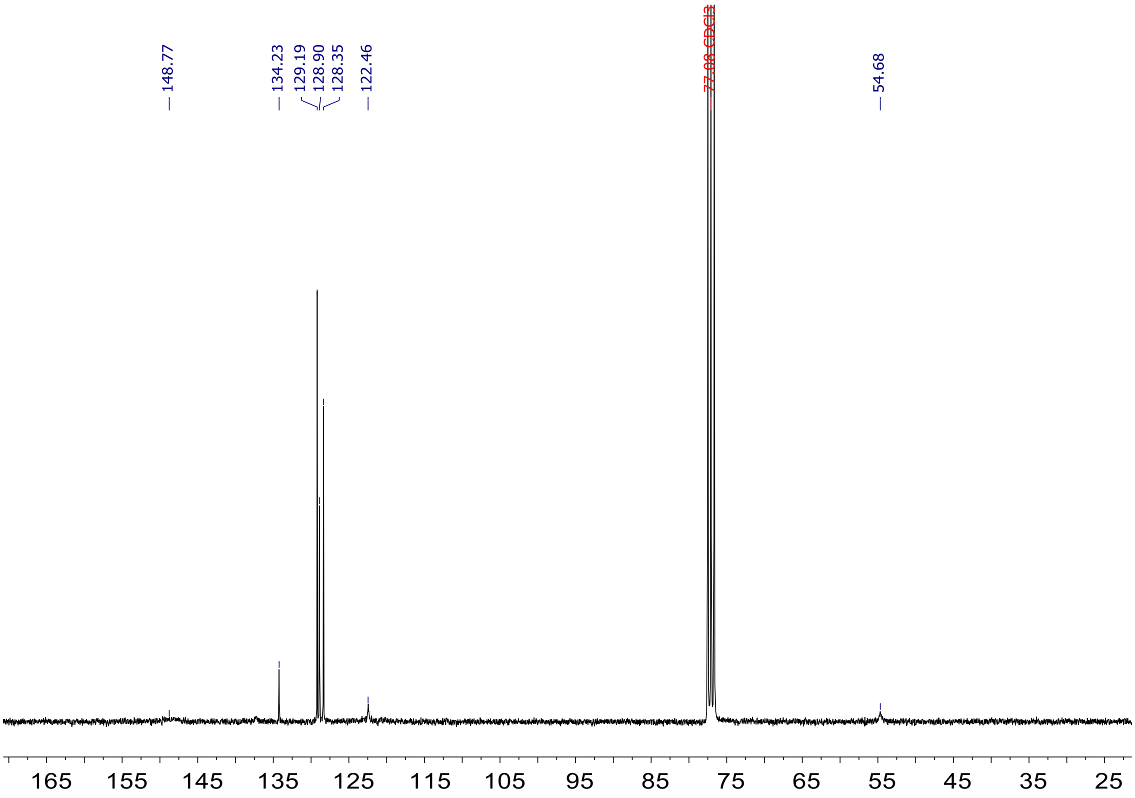

Figure S46. $^{13}C\{^1H\}$ NMR ($CDCl_3$, 75 MHz) NMR of **9h**.

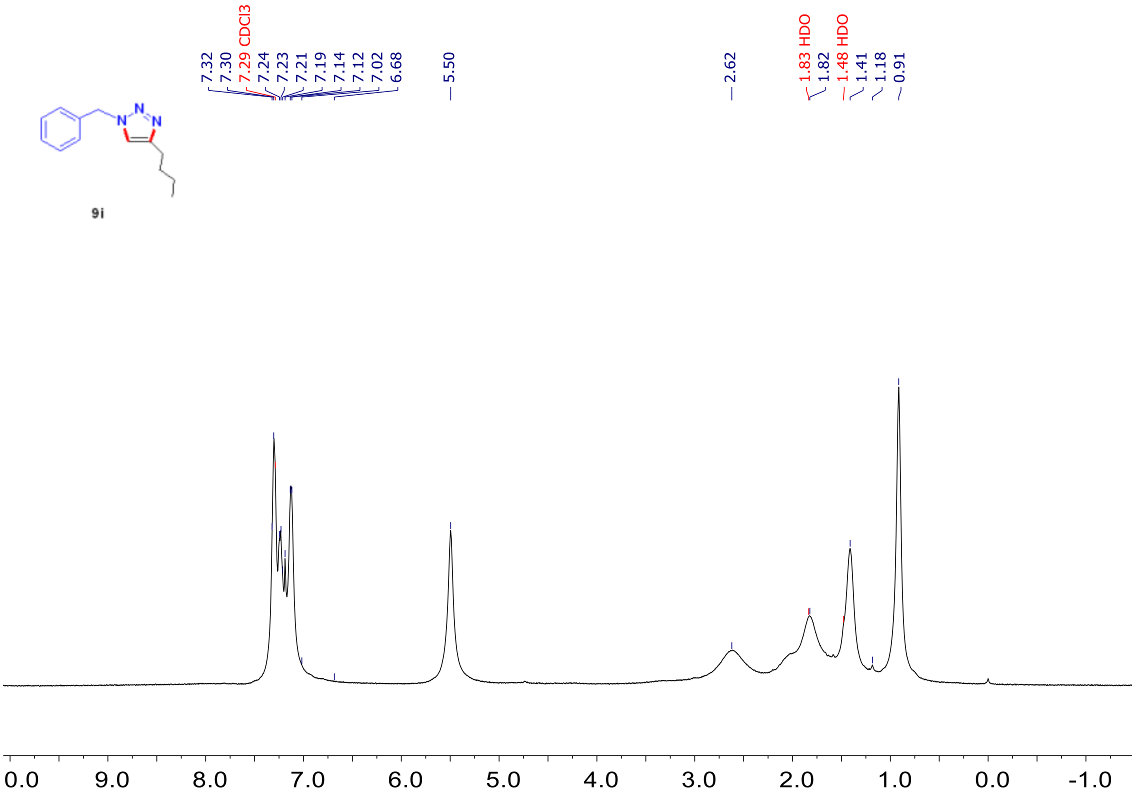

Figure S47. ^{1}H NMR ($CDCl_3$, 300 MHz) of **9i**.

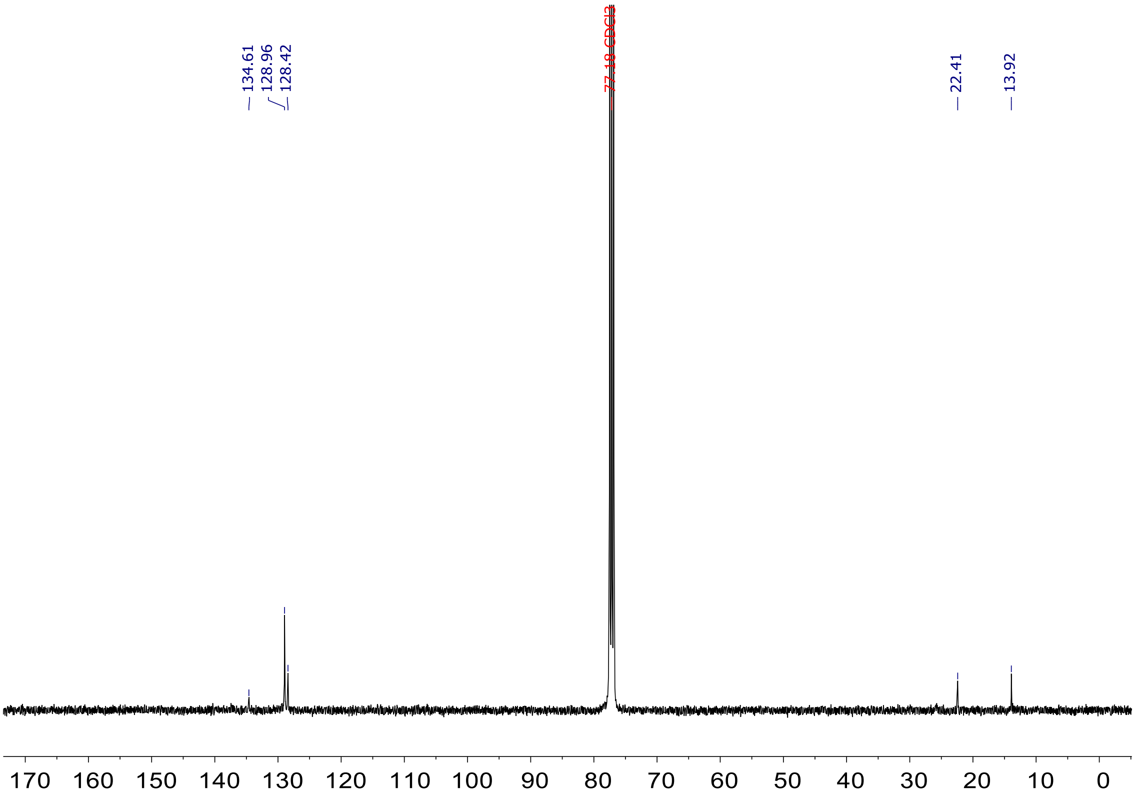

Figure S48. $^{13}C\{^{1}H\}$ NMR ($CDCl_3$, 75 MHz) of **9i**.

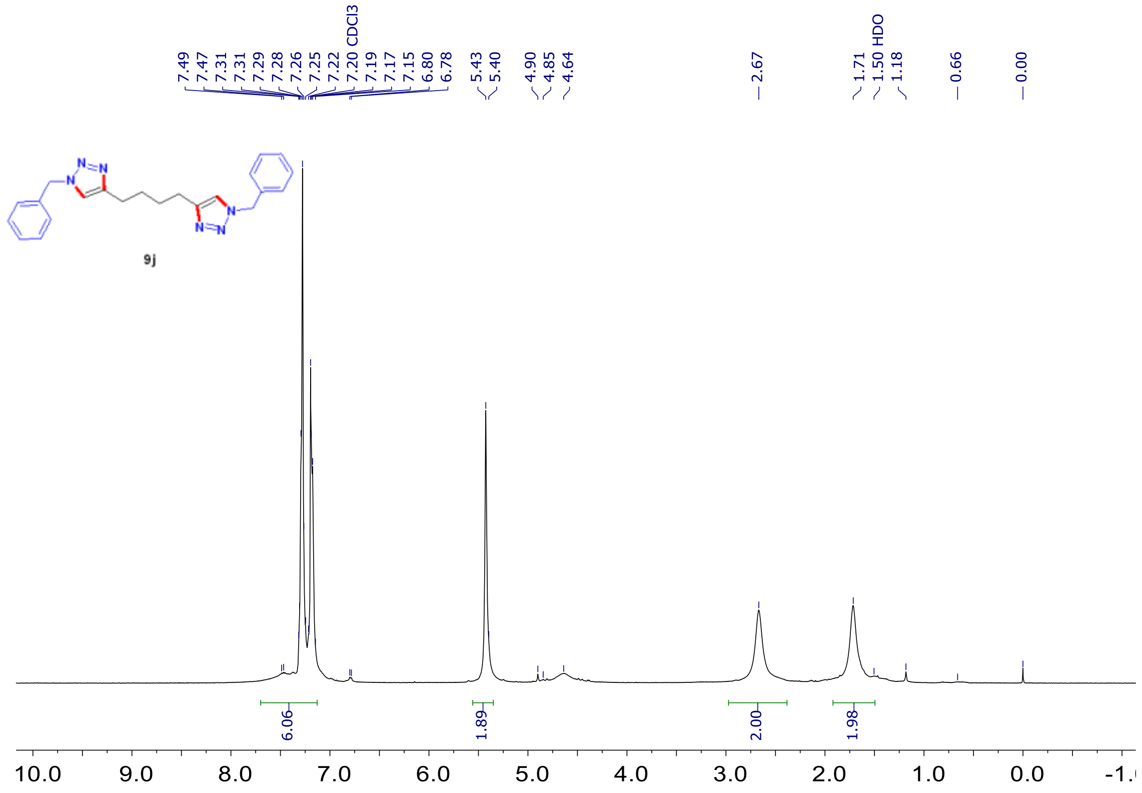

Figure S49. ^{1}H NMR ($CDCl_3$, 300 MHz) of **9j**.

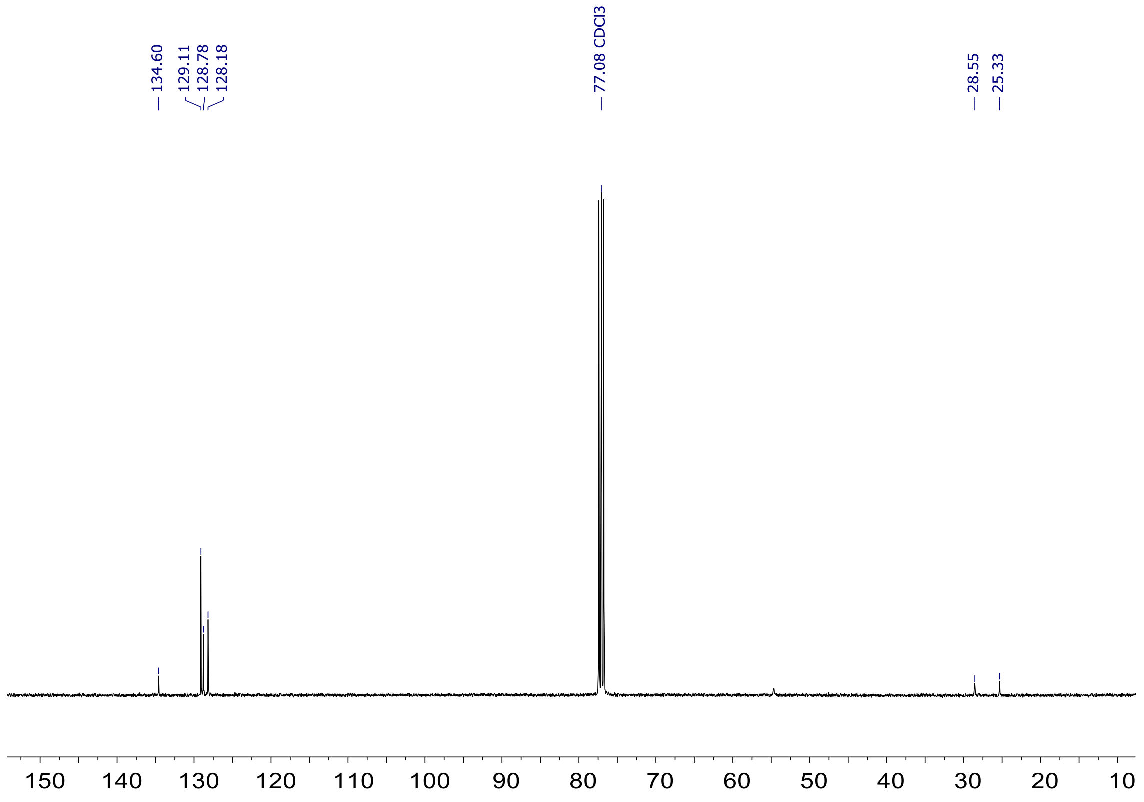

Figure S50. $^{13}C\{^{1}H\}$ NMR ($CDCl_3$, 75 MHz) of **9j**.

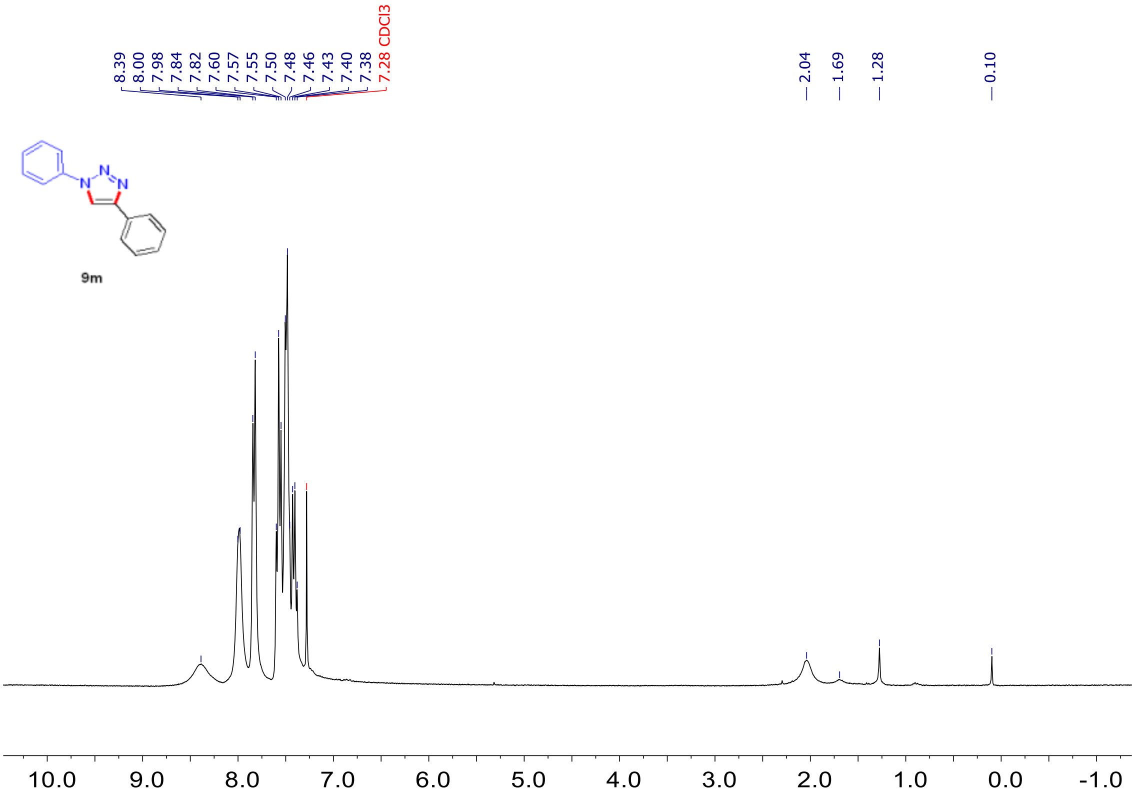

Figure S51. ^{1}H NMR ($CDCl_3$, 300 MHz) of **9m**.

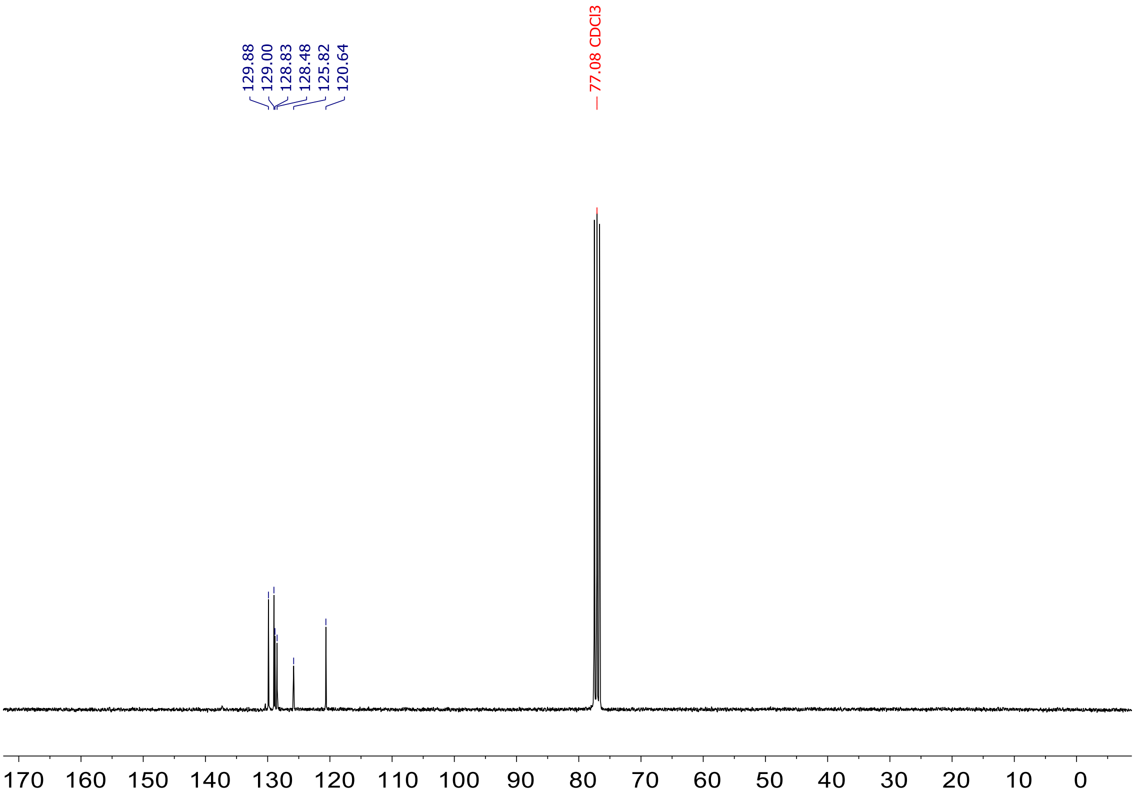

Figure S52. $^{13}C\{^{1}H\}$ NMR ($CDCl_3$, 75 MHz) of **9m**.

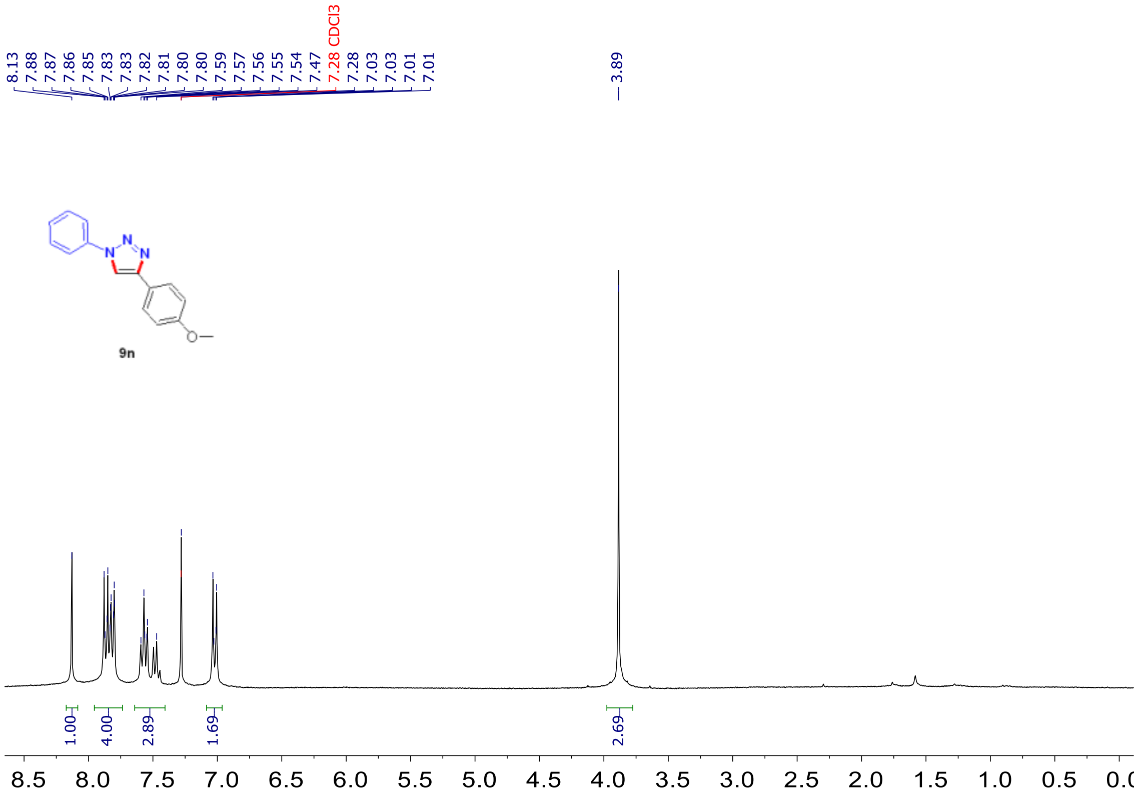

Figure S53. ^{1}H NMR ($CDCl_3$, 300 MHz) of **9n**.

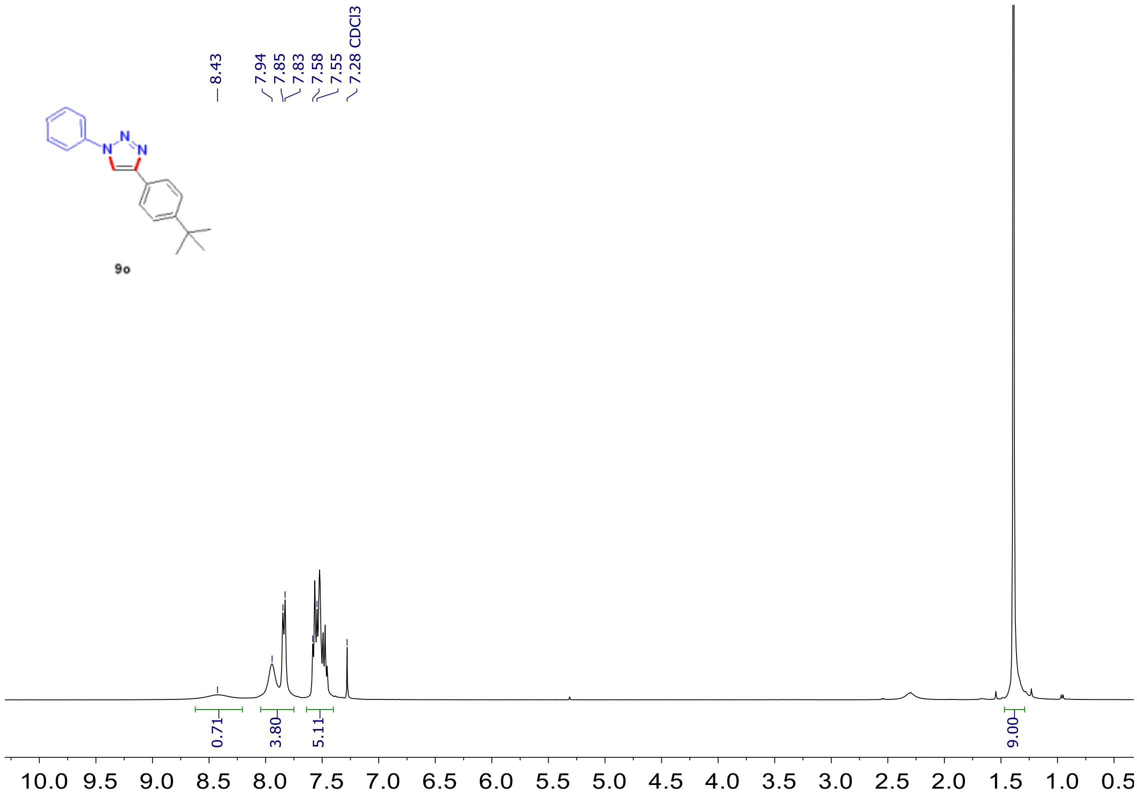

Figure S54. ^{1}H NMR ($CDCl_3$, 300 MHz) of **9o**.

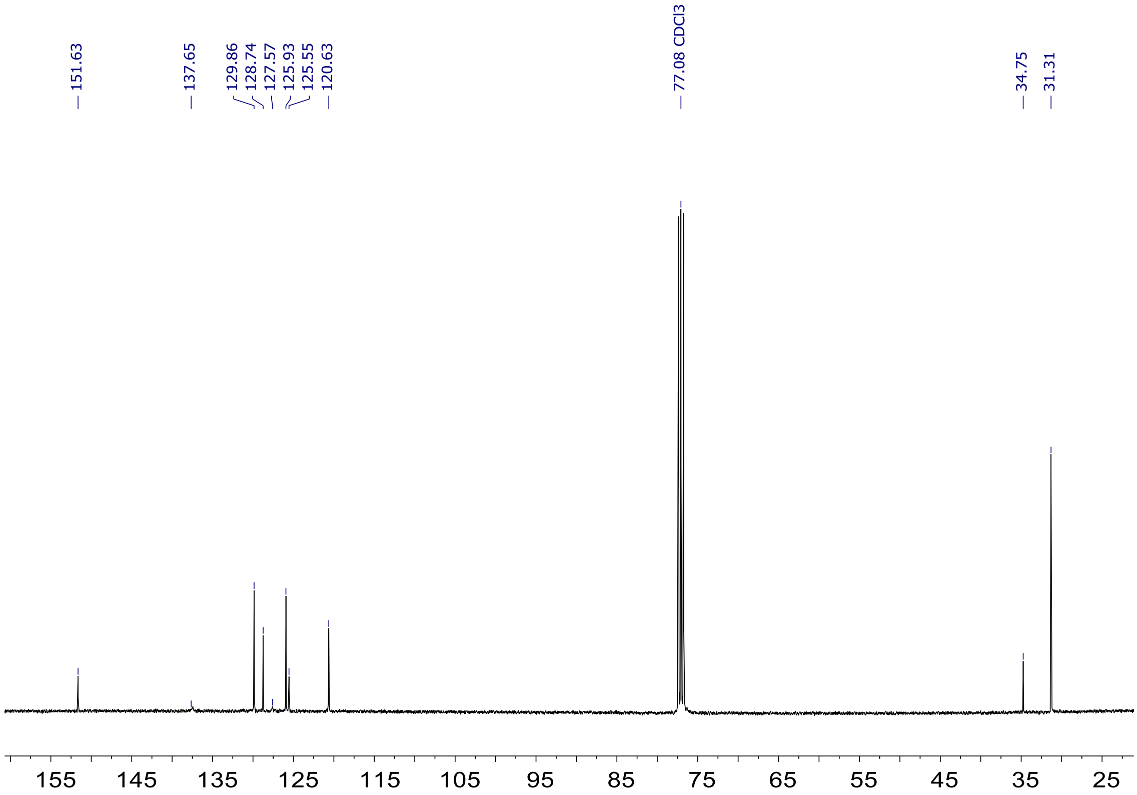

Figure S55. $^{13}C\{^{1}H\}$ NMR ($CDCl_3$, 75 MHz) of **9o**.

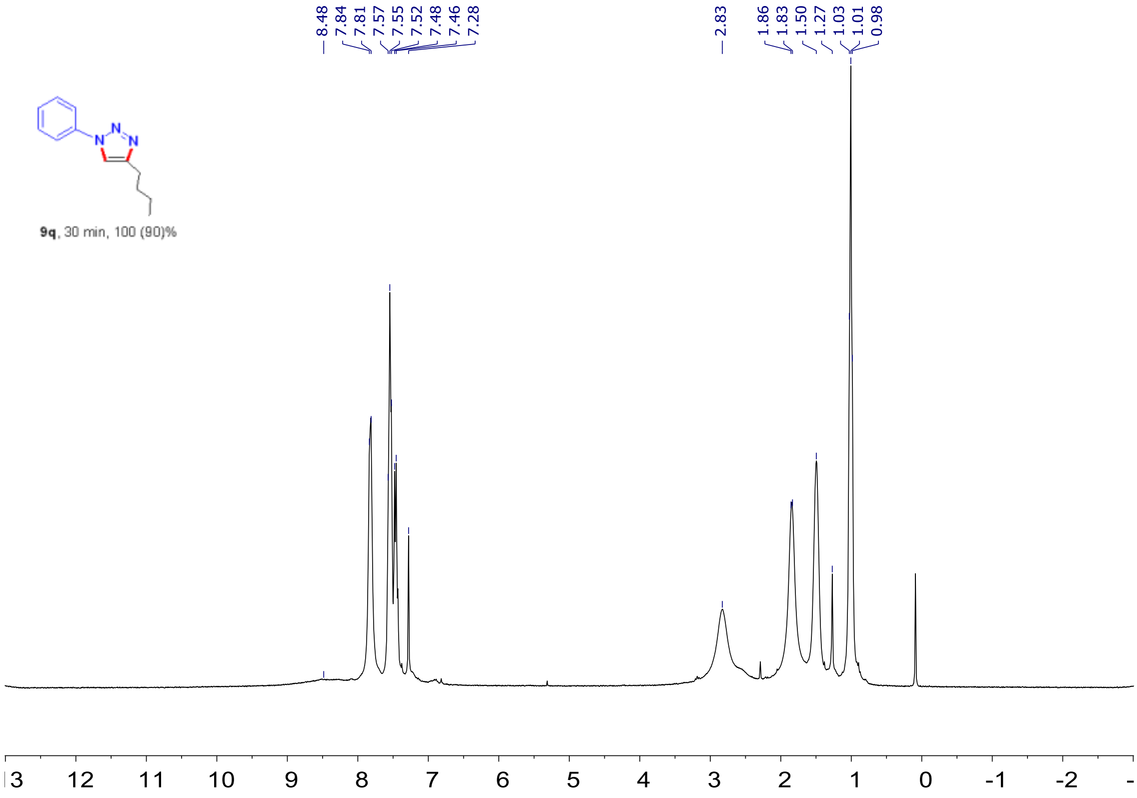

Figure S56. ^{1}H NMR ($CDCl_3$, 300 MHz) of **9q**.

6.- Deuterium labeling studies.

Experimental KIE determination. In a Schlenk tube under argon, 1 mg of catalyst **5** was dissolved in 1 mL of CD_3CN. Then, 150 μL of this solution (0.000125 mmol, 0.025 mol%) were added to a NMR tube containing 350 μL of CD_3CN. The tube was placed in an isopropanol bath at 233 K and then, 0.50 mmol (55 μL) of benzyl azide, 0.50 mmol (62 μL) of natural phenylacetylene and 0.25 mmol (35 μL) of mesitylene as internal standard were sequentially added to the frozen mixture. Finally, the tube was allowed to warm up to room temperature just before the first NMR spectrum was recorded. The reaction course was monitored by ^1H NMR spectroscopy and the benzyl azide conversion determined by integration of the methylene resonance and the aromatic proton of mesitylene (internal standard). The same procedure was followed for phenylacetylene-d_1.

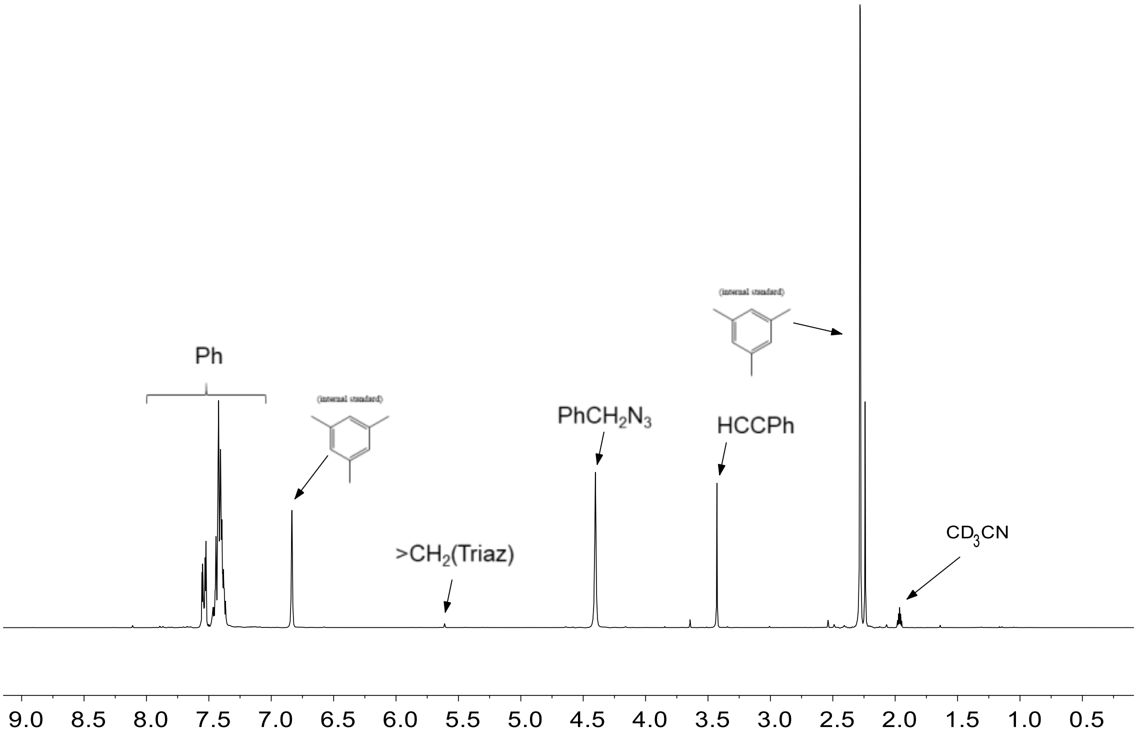

Figure S57. ^1H NMR spectrum of the 1st catalytic experiment using phenylacetylene at 298 K.

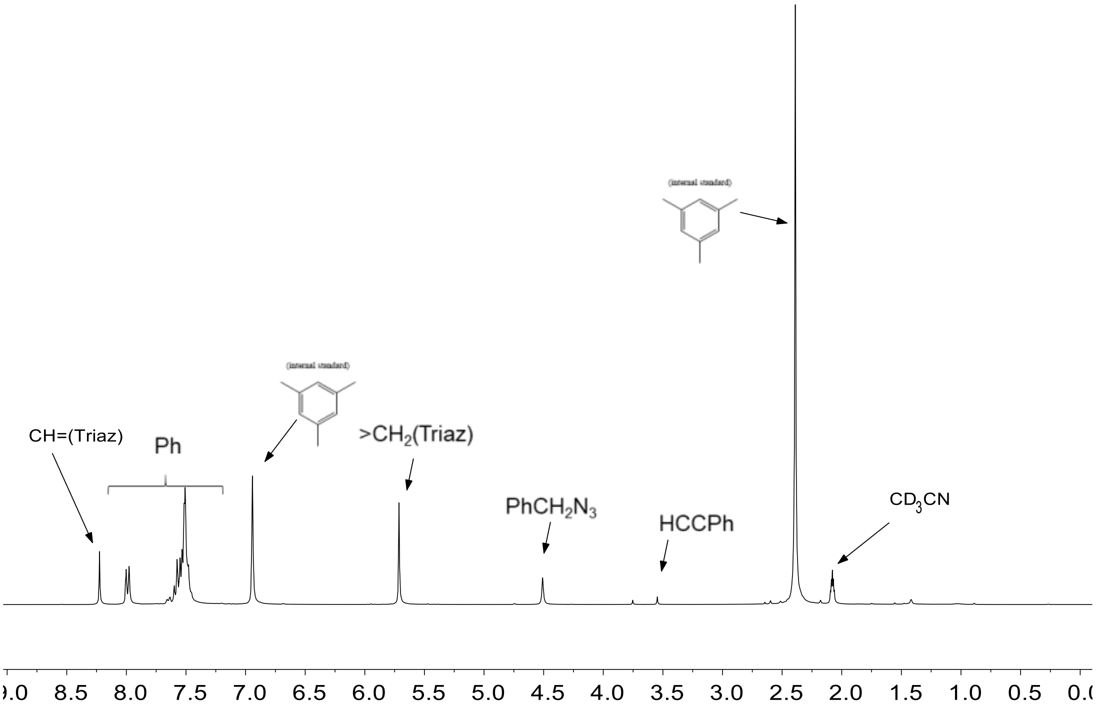

Figure S58. ^{1}H NMR spectrum of the last catalytic experiment using phenylacetylene at 298 K.

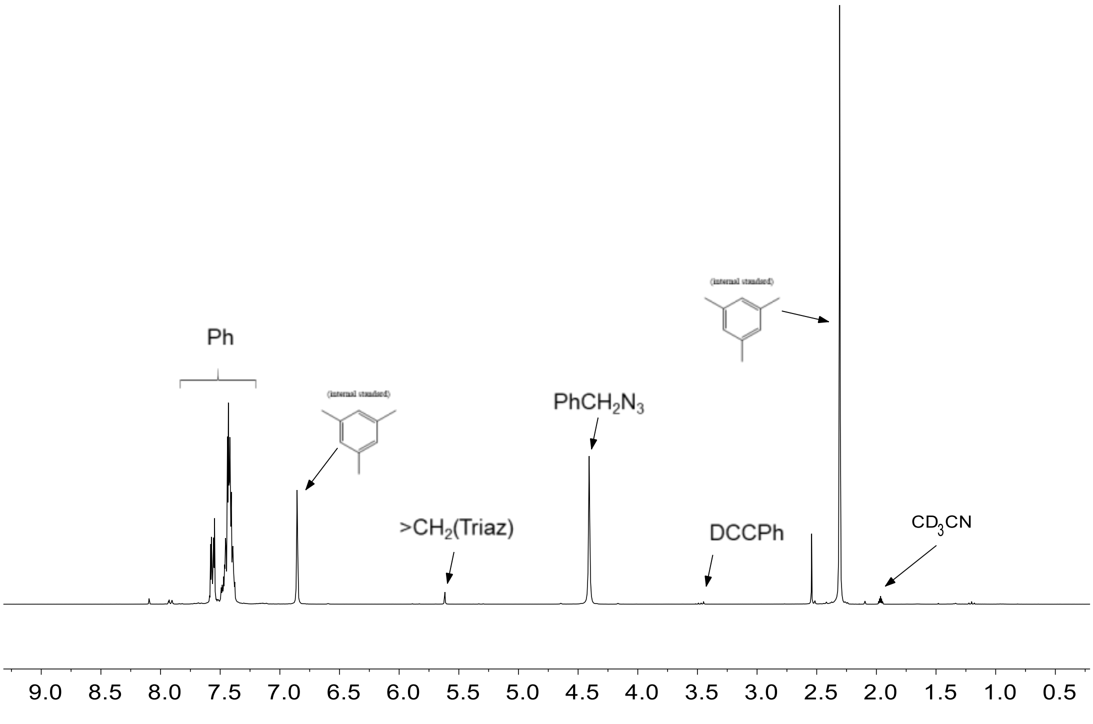

Figure S59. ^{1}H NMR spectrum of the 1st catalytic experiment using phenylacetylene-d_1 at 298 K.

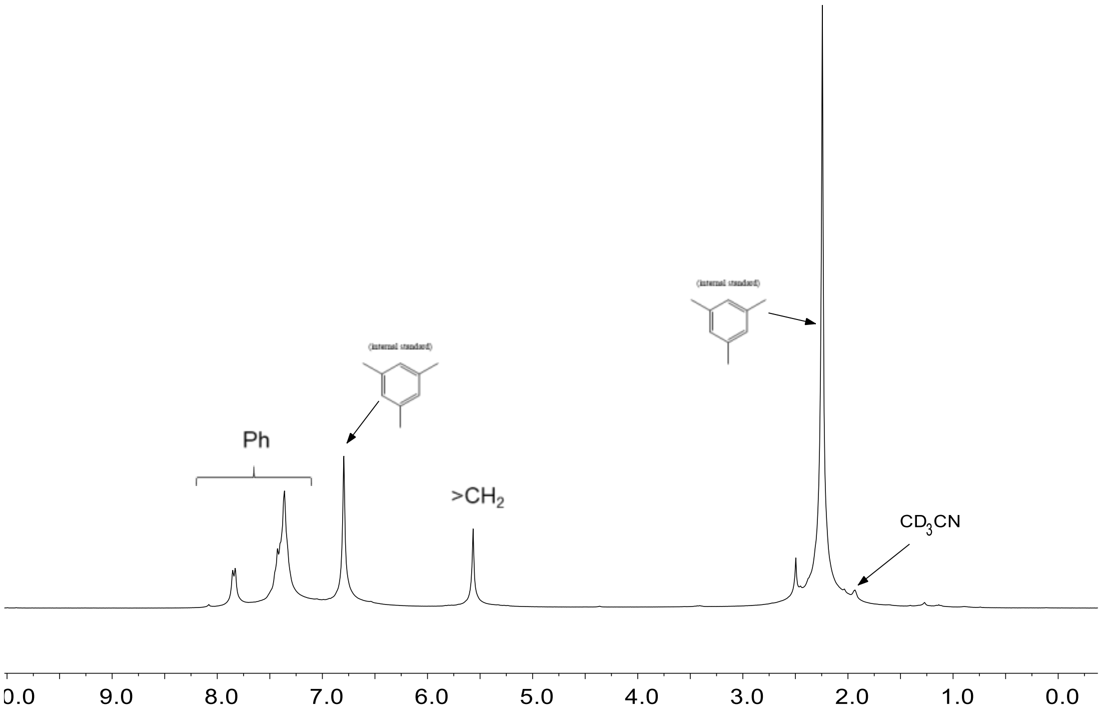

Figure S60. ^{1}H NMR spectrum of the last catalytic experiment using phenylacetylene-d_1 at 298 K.

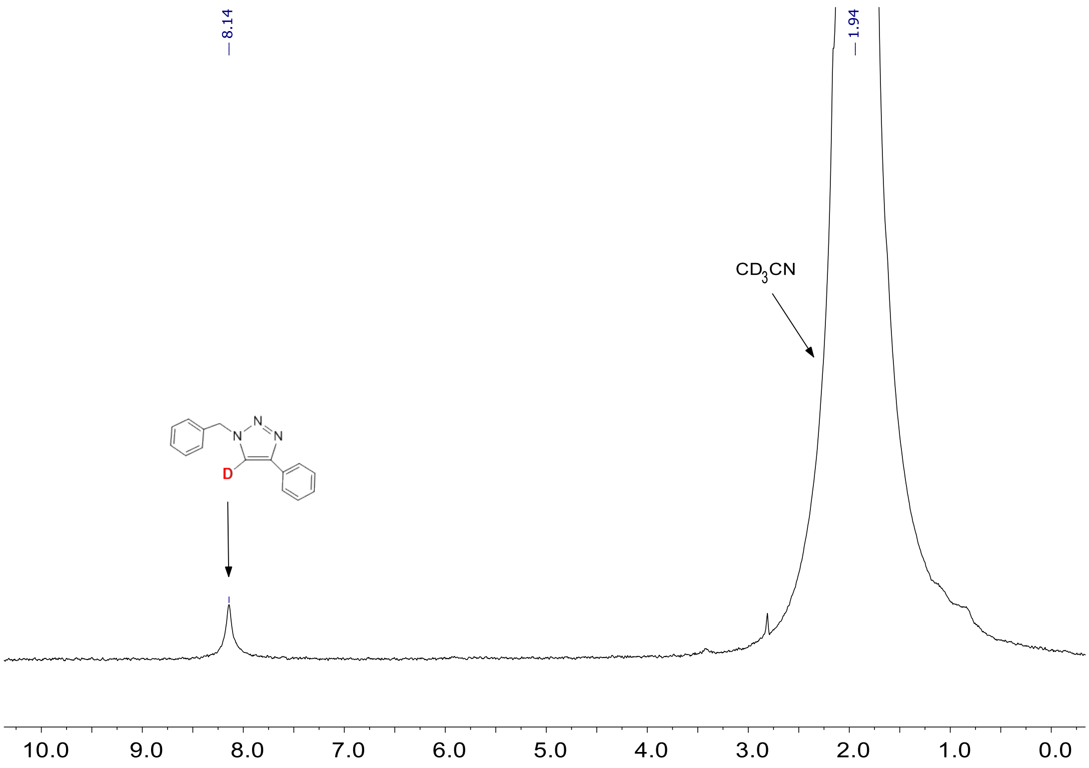

Figure S61. ^{2}H NMR spectrum of the last catalytic experiment with phenylacetylene-d_1 at 298 K.

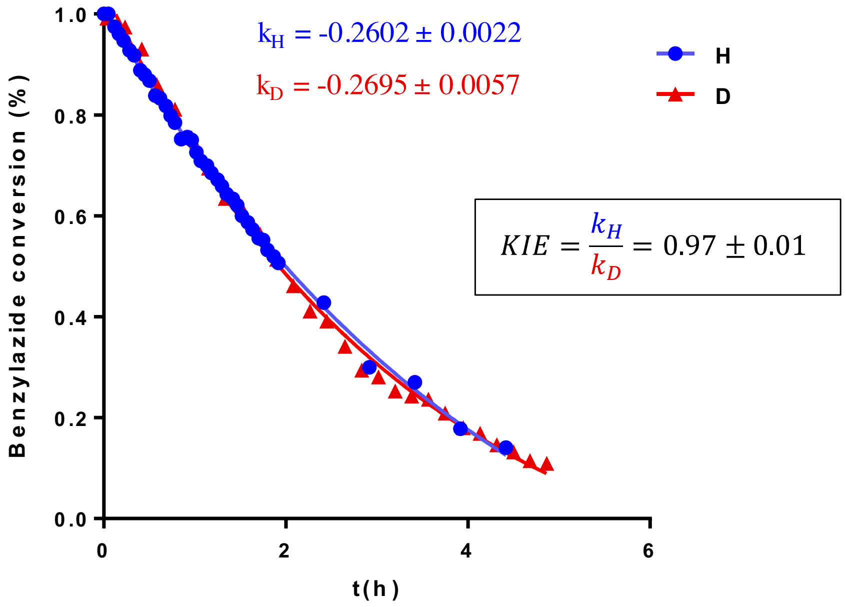

Figure S62. KIE calculation for the cycloaddition of benzyl azide and phenylacetylene catalyzed by **5** at 298 K.

7.- DFT energy data.

Table S1. Energy data for the DFT-computed intermediates and transition states. Geometrical optimizations and thermochemistry corrections with def2-SVP basis set, E(DZ), and further refinement by single point calculations with def2-TZVP, E(TZ). Corr. G(1M) corresponds to the correction to be added to E(TZ) to calculate the Gibbs energy (reference concentration of 1 mol·L^{-1}), G (1M). Absolute energies are in a.u. and relative Gibbs energies (with respect to **A**) in kcal·mol^{-1}.

	E (DZ)	Corr. G(1M)	E (TZ)	G (TZ) 1M	ΔG 1M (TZ)
**A**	-9349.9095	0.3837	-9352.0074	-9351.6237	0.0
**B**	-9658.1339	0.4811	-9660.5607	-9660.0797	1.0
**TS$_{B-C}$**	-9658.0952	0.4774	-9660.5208	-9660.0434	23.8
**C**	-9658.1488	0.4864	-9660.5797	-9660.0933	-7.5
**D**	-10093.0396	0.6079	-10095.9425	-10095.3347	-3.2
**TS$_{D-E}$**	-10093.0304	0.6109	-10095.9283	-10095.3174	7.6
**TS$_{D-E}$’**	-10093.0113	0.6095	-10095.9127	-10095.3032	16.5
**E**	-10093.0532	0.6154	-10095.9465	-10095.3311	-1.0
**TS$_{E-F}$**	-10093.0436	0.6146	-10095.9388	-10095.3242	3.4
**F**	-10093.1218	0.6170	-10096.0148	-10095.3978	-42.8
**TS$_{F-A}$**	-10093.0795	0.6090	-10095.9706	-10095.3616	-20.1
**A + P(1,4)**[a]	-9349.9095	0.3837	-9352.0074	-9351.6237	-54.5
**TS$_{F-C}$**	-10401.3006	0.7109	-10404.5298	-10403.8189	-19.9
**C + P(1,4)**[a]	-9658.1488	0.4864	-9660.5797	-9660.0933	-62.0
**Benzyl azide**	-434.8656	0.1005	-435.3487	-435.2483	0.0
**Ph-acetylene**	-308.2038	0.0823	-308.5400	-308.4576	
**TS$_{1-4}$**	-743.0450	0.1973	-743.8578	-743.6605	28.5
**TS$_{1-5}$**	-743.0477	0.2029	-743.8604	-743.6575	30.4
**P(1,4)**[a]	-743.1932	0.2062	-743.9990	-743.7928	-54.5
**P(1,5)**[b]	-743.1919	0.2096	-743.9986	-743.7889	-52.1
**4-tetranuclear**	-18503.7838	0.6002	-18507.7700	-18507.1697	
**4-dinuclear**	-9251.8711	0.2873	-9253.8721	-9253.5848	0.1
**5-tetranuclear**	-18699.8622	0.7856	-18704.0435	-18703.2578	
**5-dinuclear (A)**	-9349.9095	0.3837	-9352.0074	-9351.6237	6.6
**6-tetranuclear**	-18699.8782	0.7832	-18704.0629	-18703.2798	
**6-dinuclear**	-9349.9254	0.3824	-9352.0215	-9351.6392	0.9

a) P(1,4): 1-benzyl-4-phenyl-1H-1,2,3-triazole

b) P(1,5): 1-benzyl-5-phenyl-1H-1,2,3-triazole

8.- References.

(1) Alonso, F.; Moglie, Y.; Radivoy, G.; Yus, M. Unsupported Copper Nanoparticles in the 1,3-Dipolar Cycloaddition of Terminal Alkynes and Azides. *Eur. J. Org. Chem.* **2010**, *10*, 1875–1884.

(2) Sau, C. S.; Roy, S. H.; Sen, T. K.; Mullangi, D.; Mandal, S. K. An Abnormal N-Heterocyclic Carbene–Copper(I) Complex in Click Chemistry. *Adv. Synth. Catal.* **2013**, *355*, 2982–2991.

(3) Jia, Z.; Wang, K.; Li, T.; Tan, B.; Gu, Y. Functionalized hypercrosslinked polymers with knitted N-heterocyclic carbene–copper complexes as efficient and recyclable catalysts for organic transformations. *Catal. Sci. Technol.* **2016**, *6*, 4345–4355.

(4) Asano, K.; Matsubara, S. Effects of a Flexible Alkyl Chain on a Ligand for CuAAC Reaction, *Org. Lett.* **2010**, *12*, 4988–4991.

(5) Nakamura, T.; Terashima, T.; Ogata, K.; Fuzuzawa S.-I. Copper(I) 1,2,3-Triazol-5-ylidene Complexes as Efficient Catalysts for Click Reactions of Azides with Alkynes. *Org. Lett.* **2011**, *13*, 620–623.

(6) Yamaguchi, K.; Oishi, T.; Katayama, T.; Mizuno, N. A Supported Copper Hydroxide on Titanium Oxide as an Efficient Reusable Heterogeneous Catalyst for 1,3-Dipolar Cycloaddition of Organic Azides to Terminal Alkynes. *Chem. Eur. J.* **2009**, *15*, 10464–10472.
